# Taxonomic and functional profiling of fecal metagenomes for the early detection of colorectal cancer

**DOI:** 10.3389/fonc.2023.1218056

**Published:** 2023-08-03

**Authors:** Xudong Wu, Zhimin Tang, Rongsong Zhao, Yusi Wang, Xianshu Wang, Side Liu, Hongzhi Zou

**Affiliations:** ^1^ Creative Biosciences (Guangzhou) CO., Ltd, Guangzhou, Guangdong, China; ^2^ Department of Gastroenterology, Nanfang Hospital, Southern Medical University, Guangzhou, Guangdong, China; ^3^ Department of Colorectal Surgery, Guangdong Provincial Key Laboratory of Colorectal and Pelvic Floor Diseases, Guangdong Institute of Gastroenterology, The Sixth Affiliated Hospital, Sun Yat-sen University, Guangzhou, Guangdong, China

**Keywords:** metagenomic sequencing, fecal microbiota, bacterial biomarker, microbial taxonomy, functional pathways

## Abstract

**Objectives:**

This study aimed to identify colorectal cancer (CRC)-associated phylogenetic and functional bacterial features by a large-scale metagenomic sequencing and develop a binomial classifier to accurately distinguish between CRC patients and healthy individuals.

**Methods:**

We conducted shotgun metagenomic analyses of fecal samples from a ZhongShanMed discovery cohort of 121 CRC and 52 controls and SouthernMed validation cohort of 67 CRC and 44 controls. Taxonomic profiling and quantification were performed by direct sequence alignment against genome taxonomy database (GTDB). High-quality reads were also aligned to IGC datasets to obtain functional profiles defined by Kyoto Encyclopedia of Genes and Genomes (KEGG). A least absolute shrinkage and selection operator (LASSO) classifier was constructed to quantify risk scores of probability of disease and to discriminate CRC from normal for discovery, validation, Fudan, GloriousMed, and HongKong cohorts.

**Results:**

A diverse spectrum of bacterial and fungi species were found to be either enriched (368) or reduced (113) in CRC patients (q<0.05). Similarly, metabolic functions associated with biosynthesis and metabolism of amino acids and fatty acids were significantly altered (q<0.05). The LASSO regression analysis of significant changes in the abundance of microbial species in CRC achieved areas under the receiver operating characteristic curve (AUROCs) of 0.94 and 0.91 in the ZhongShanMed and SouthernMed cohorts, respectively. A further analysis of Fudan, GloriousMed, and HK cohorts using the same classification model also demonstrated AUROC of 0.80, 0.78, and 0.91, respectively. Moreover, major CRC-associated bacterial biomarkers identified in this study were found to be coherently enriched or depleted across 10 metagenomic sequencing studies of gut microbiota.

**Conclusion:**

A coherent signature of CRC-associated bacterial biomarkers modeled on LASSO binomial classifier maybe used accurately for early detection of CRC.

## 1 Introduction

Colorectal cancer (CRC) ranks third in incidence and second in mortality of all malignancies worldwide. It is predicted to surpass lung cancer and breast cancer in incidence around 2030 and 2040, respectively, to become the most dominant malignancy globally (1). In 2016, the number of newly diagnosed cases and deaths reached 408,000 and 196,000 in China, making CRC the second most common malignancy and the fourth deadliest cancer in the country (2). Over recent decades, CRC incidence and mortality rates have been continuously rising as living standard improves and lifestyle changes. Furthermore, over 50% of the new cases in China have been diagnosed at advanced stage, leading to poor prognosis and survival (3). Several approaches such as fecal immunochemical test (FIT), multitarget stool DNA test, stool-based DNA methylation test, and virtual colonoscopy have been adopted for CRC detection and screening (4). Early detection of CRC significantly reduces its mortality rate and hence the disease burden of the malignancy.

In recent decade, CRC-associated microbial genera and species have been identified by 16S rRNA or metagenomic sequencing to be either enriched or depleted in fecal samples or tissue specimens from CRC patients. Consistent composition changes in gut microbiota have been identified by 16S rRNA amplicon sequencing, and increased abundance of certain bacterial genera has been reported in tumor tissues compared to adjacent normal (5). Shah et al. analyzed the abundance of bacterial species in stool and identified a dozen of effective bacterial genus markers including *Porphyromonas*, *Peptostreptococcus*, *Parvinonas*, *Akkermansia*, and *Fusobacterium* for CRC screening. More importantly, a classifier constructed based on these microbial markers and clinical information could discriminate CRC and control with an area under the receiver operating characteristic curve (AUROC) value of 0.833 (6). Most recently, Yang and colleagues assessed fecal microbiota dysbiosis using 16S rRNA sequencing to observe enriched and depleted genera in old- and young-onset CRC. Random forest (RF) classifier constructed based on unique panels of microbial markers could discriminate CRC cases and normal controls on family and genus levels with AUROC values of 0.851 and 0.898, respectively (7). Yu et al. took a step further in utilizing metagenomic sequencing to quantify fecal microbiome alterations and identified a novel set of 20 bacterial genes to be used for CRC screening. The selected gene markers were able to discriminate CRC from control with AUROC values of 0.71–0.77 among different ethnic cohorts (8). Thomas et al. performed a meta-analysis of 969 fecal metagenomes across Chinese, Austrian, American, German, and French cohorts and found that seven common enriched bacterial species including *Bacteroides fragilis*, *Fusobacterium nucleatum*, *Porphyromonas asaccharolytica*, *Parvimonas micra*, *Prevotella intermedia*, and *Alistipes finegoldii*. These microbial signatures achieved accurate CRC prediction with an average AUROC of 0.84 (9). Furthermore, Coker et al. discovered that a set of 14 fungal biomarkers in stool could also be used to accurately predict CRC with higher AUROC of 0.74–0.93 in independent discovery and validation cohorts (10). The most robust predicting performance was achieved by Chen et al. using a panel of eight serum metabolites associated with metagenomic profiles of gut microbiome. The unique set of metabolomic biomarkers was selected using a least absolute shrinkage and selection operator (LASSO) algorithm and used for CRC prediction yielding AUROC of 0.98 and 0.92 for the modeling and validation cohorts (11). Taken together, these accumulating evidence suggest that metagenomic profiles and 16S rRNA amplicons can be exploited for early and accurate detection of CRC.

In the current study, we conducted a metagenomic shotgun sequencing of fecal samples from two hospital-based case–control cohorts and analyzed the difference in relative abundance of bacterial species and metabolic functions between CRC patients and healthy individuals. Based on ZhongShanMed discovery cohort, we constructed a binomial classifier that could achieve significantly higher AUROC values than two other models in both ZhongShanMed and another independent SouthernMed validation cohort. Additionally, we examined the accuracy of CRC prediction using the classifier by analyzing publicly available metagenomic sequencing data from three Chinese cohorts. Finally, CRC-associated bacterial features revealed by the current investigation were assessed by meta-analysis across 10 fecal metagenome studies.

## 2 Materials and methods

### 2.1 Study design and cohorts

All subjects underwent standard colonoscopy examinations at The Sixth Affiliated Hospital, Sun Yat-sen University (ZhongShanMed) and Nanfang Hospital (SouthernMed). The two studies were approved by Institutional Review Board of respective hospitals (2013ZSLYEC-028 and NFEC-201705-Q1). Written informed consent was obtained for all subjects. Patients with definitive diagnosis of CRC based on colonoscopy examinations and pathological reports were recruited. Individuals with negative colonoscopy outcome or carrying benign lesion of hemorrhoids were included as normal controls. There was no age restriction on the cohort, but subjects considered unsuitable for colonoscopy, such as those with pregnancy, hypertension, or heart diseases, were excluded. Clinical and pathological information for both cases and controls were recorded for further bioinformatics and statistical analyses. Patients or the public were not involved in the design, conduct, reporting, and dissemination of our research.

### 2.2 Sample preparation, microbial DNA extraction, and metagenomic sequencing

Fresh stool from each subject was collected before colonoscopy examinations at each respective hospital. For DNA extraction, the HiPure Stool DNA Kit (Magen, China) was used with some modifications. Briefly, 1 ml of stool suspension in preservation buffer was centrifuged at 15,000 rpm for 2 min. The pellet was resuspended in 4M guanidine isothiocyanate and 5% sodium N-dodecanoylsalcosinate and incubated for 1 h at 70°C with shaking at 1,800 rpm. The mixture was then centrifuged at 14,000 *g* for 1 min, and the supernatant was collected. The resulting pellet was suspended in 0.5 ml TENP and centrifuged at 14,000 *g* for 1 min. The supernatant was collected, and the pellet was resuspended in 0.5 ml TENP and centrifuged again. The supernatant was combined (approximately 1 ml) and subjected to one cycle of freezing at −80°C for 60 min followed by thawing at room temperature. The mixture was then centrifuged at 14,000 *g* for 1 min, and the supernatant was collected and subjected to purification steps using HiPure Stool DNA Kit based on manufacturer’s instructions. DNA concentration was measured using a Qubit Fluorometer (Thermo Scientific, USA) and NanoDrop spectrophotometer (Thermo Scientific, USA). The quality of isolated DNA was examined by 1% agarose gel electrophoresis. All DNA samples were stored in −20°C freezer before subsequent processing.

For library preparation, microbial DNA samples were fragmented by a Covaris M220 sonicator and size selected to 410–440 bp using DNA Clean Beads (MGI Tech Co. Ltd, China). For metagenomic sequencing, 50 ng of fragmented microbial genomic DNA was used. End-repair and A-tailing reactions and ligation of UDB adapter (MGI Tech Co. Ltd, China) were prepared with MGIEasy Universal DNA Library Prep Set (MGI Tech Co. Ltd, China) according to manufacturer’s protocol. The library was amplified with polymerase in MGIEasy Universal DNA Library Prep Set for seven cycles and cleaned up on 1× DNA Clean Beads. The sequencing library was sequenced on an MGISEQ-2000 (MGI Tech Co. Ltd, China) using MGISEQ-2000RS high-throughput set (PE150) (MGI Tech Co. Ltd, China). We obtained a total of 2,556 Gb with an average of 30M reads per sample before quality control and preprocessing.

All pair-ended reads underwent quality control to retain high-quality reads for subsequent analysis (12). The KneadData (version 0.7.4) (13) and Trimmomatic (version 0.39) (14) programs were used to remove low-quality reads and non-microbial sequences to retain high-quality microbial sequencing reads (SLIDINGWINDOW:4:20 MINLEN:50 LEADING:3 TRAILING:3). The following non-microbial genomes (hg38, mm10, rn6, susScr3, bosTau8, canFam3, felCat8, and galGal4) were retrieved from UCSC Genome Browser. The plasmid and vector sequences were collected and manually curated from Plsdb and SURPIrt. The matched reads considered potentially host associated and laboratory associated were removed as contaminants.

### 2.3 Microbial taxonomic profiling and tree construction

The custom database was reconstructed based on genome sequences annotated by the genome taxonomy database (GTDB) (15) and PathSeq (16). The high-quality reads were aligned to the microbial genomic sequences in KLM2022 using BWA (17). Taxonomic hierarchy information was retrieved from GTDB, and a taxonomy tree was constructed using GraPhlAn (version 1.1.3) (18). The species with an average abundance of 1E−5 or higher and q≤1E−4 (meta-deconfounder analysis) are shown. The phylogenetic levels including domain, phylum, family, genus, and species were used.

### 2.4 Microbial functional profiling and pathway characterization of KEGG orthologs

The 9,879,896 gene sequences of IGC database were retrieved and indexed (19). The high-quality reads were aligned against the IGC datasets, and alignments were then filtered to only retain those >95% sequence identity. Only the highest scoring alignments were kept for each read. The relative abundance of each Kyoto Encyclopedia of Genes and Genomes (KEGG) orthologous group was estimated by summing the relative abundances of genes in the same KEGG orthologous group as provided by MOCAT2 (20).

KEGG Orthology (KO) annotation information was retrieved from KEGG, and a functional hierarchy tree was constructed using GraPhlAn. Briefly, the KOs with q≤1E−4 (meta-deconfounder analysis) are shown, and we manually curated the KEGG modules listed under the categories of “Microbial metabolism in diverse environments” and “Amino acid metabolism.”

### 2.5 Construction of microbiota models and flux balance analysis

The AGORA reference reconstructions and the corresponding genome sequences were retrieved from the online website, https://www.vmh.life/, and the trimmed FASTQ files were aligned to the indexed AGORA reference sequences. Coverage profiles were obtained by SAMTools (version 1.9) (21), and qualifications for each sample were matched individually to the AGORA models on the genus and species levels. Assuming a steady state for all fluxes in the biological system, the growth rates were estimated by MICOM in each sample, and the cooperative trade-off was applied to obtain the approximate fluxes (22).

### 2.6 Confounder analysis

To evaluate and de-confound the effects of age, gender, and body mass index (BMI), multivariate association with linear model algorithm was used for multi-variable association testing between phenotypes and microbial taxa (23, 24). Potential confounders with continuous values were transformed into categorical data as quartiles.

### 2.7 Biomarker identification using three prediction models

We constructed classification models based on the species profiles using three different methods, LASSO logistic regression (25), RF (26), and support vector machine learning (SVM). For LASSO logistic regression, the models were trained by fivefold stratified cross-validation testing. The caret R package (version 6.0.90) (27) was adopted to partition the discovery cohort data (ZhongShanMed) into randomized training and testing sets in an 80%–20% manner. The binomial GLMnet models (version 4.1.3) (28, 29) were then trained by 20 iterations of fivefold cross-validation to optimize values of hyperparameter of penalty (lambda, values range from 1E−5 to 1E−1) using Cohen’s Kappa as the performance metric. For each training set, this yielded a collection of two-classes (CRC and healthy control) binomial classifiers. The accuracy of the model was then examined using area under the receiver operating characteristic curve (AUROC) values. The abundance threshold of 1E−5 and 1E8 were used as abundance threshold and pseudo-count during log10 transformation, respectively, to avoid non-finite values. The features were standardized as z-scores by centering to zero mean and dividing by the standard deviation of each feature. Feature contributions to the models were output as feature importance and computed using the percentage absolute values of the regression coefficients.

For RF prediction, abundance threshold of 1E−3 was adopted to remove markers with low overall abundance, and relative abundance was log10 transformed after padding a pseudo-count of 1E−5 to avoid non-finite values. Two steps were carried out in the RF models. In the first step, the optimal number of features was determined using the recursive feature elimination method with parameter step=1. In the second, the models were trained by fivefold stratified cross-validation testing. The training datasets were resampled for 20 times of partitions). Feature contributions to the models were output as feature importance.

For SVM prediction, same thresholds as RF were used for bacterial relative abundance and pseudo-count during transformation. Two steps were carried out in the SVM models. In the first step, the rank of feature importance was determined using the mSVM-RFE algorithm (https://github.com/johncolby/SVM-RFE) (30), and the 30 most important features were selected. In the second, the models were trained by fivefold stratified cross-validation testing. The training datasets were resampled for 20 times of partitions. Feature contributions to the models were output as feature importance.

### 2.8 POD calculation for metagenomic datasets of Fudan, GloriousMed, and HongKong cohorts

As the calculation of probability of disease (POD) was based on species with relative abundance larger than 1E−5, the 16,544 species were to be retrieved to accelerate the computation using a cutoff of the average relative abundance of 1E−8 across all 284 sequenced samples. We compiled corresponding genome sequences to construct a custom database in which the customized datasets included 15,993 bacteria, 65 archaebacteria, 144 fungi, and 341 viruses. Sequencing data from metagenomes from Fudan (7), GloriousMed (31), and HK (9) cohorts were profiled and modeled by LASSO regression analysis as follows.

For Fudan cohort, taxonomic profiling was performed on sequencing data of 200 metagenomes retrieved from the National Center for Biotechnology Information (NCBI) GEO (https://www.ncbi.nlm.nih.gov/bioproject/PRJNA763023/). The relative abundance was log-transformed and scaled by the corresponding means and standard deviation of species in model training phrase (ZhongShanMed Cohort). For each sample in the cohort, the POD risk scores were calculated from 100 trained models and averaged; then, the AUROC were estimated for the whole cohort, younger group, and elderly group, respectively.

For GloriousMed cohort, metagenomic sequencing data of 166 subjects from the EBI ENA Browser (https://www.ebi.ac.uk/ena/browser/view/PRJNA731589) were downloaded, and the taxonomic profiling was performed using the custom database. The samples with aligned reads smaller than 5M were filtered out, and the relative abundance was log transformed and scaled by the corresponding means and standard deviation of species in model training phrase (ZhongShanMed Cohort). For each sample in the cohort, the POD risk scores for 82 healthy and 76 CRCs were calculated from 100 trained models and averaged; then, the AUROCs were estimated for the whole cohort.

For the HK cohort, after the CRC samples with T2D were filtered out, sequencing data of 83 remaining metagenomes were downloaded from the EBI ENA Browser (https://www.ebi.ac.uk/ena/browser/view/PRJEB10878). The taxonomic profiling using the custom database were performed, and samples with aligned reads smaller than 5M were excluded. The relative abundance was log-transformed and scaled by the corresponding means and standard deviation of species in model training phrase (ZhongShanMed Cohort). For each sample in the cohort, the POD risk scores for 38 healthy and 43 CRCs were calculated from 100 trained models and subsequently averaged before AUROC values were estimated for the whole cohort.

### 2.9 Identification of cross-cohort microbial signatures through meta-analysis of colorectal cancer datasets

The nine publicly available and geographically diverse metagenomic studies of colorectal cancer were obtained in CuratedMetagenomicData, and the corresponding taxonomic profiling of the samples annotated by healthy or CRC were retrieved. To incorporate our metagenomic datasets into this large microbiome repository (32), the sequencing datasets were annotated by MetaPhlAn2 (version 2.7.7) (33), and the taxonomic profiling were obtained. Based on a total of 10 cohorts including ours (**Supplementary Table S8**) (8, 32, 34), the meta-analysis of standardized mean difference was performed for the identified microbial species in metafor package (version 3.0.2) using the random effects model (35).

### 2.10 Statistical analyses

A comparison between quantitative data was performed using the Mann–Whitney U-test. Receiver operating characteristic (ROC) curve was used to evaluate the performance of multi-variables that differentiate between certain groups. The FDR-corrected p-values (q-values) were obtained by de-confounder analysis using the metadeconfoundR (version 0.2.8) (https://github.com/TillBirkner/metadeconfoundR) pipeline implemented in the R package. The principal coordinate analysis (PCoA) was performed based on the Bray–Curtis dissimilarities at the species level using vegdist and adonis2 in a vegan package (version 2.5.7) (36). All p- and q-values were two-tailed, and p<0.05 or q<0.05 was considered statistically significant. All data were analyzed by R 3.6.1 software (http://www.R-project.org).

## 3 Results

### 3.1 Cohort information and study design

**Figure 1:**
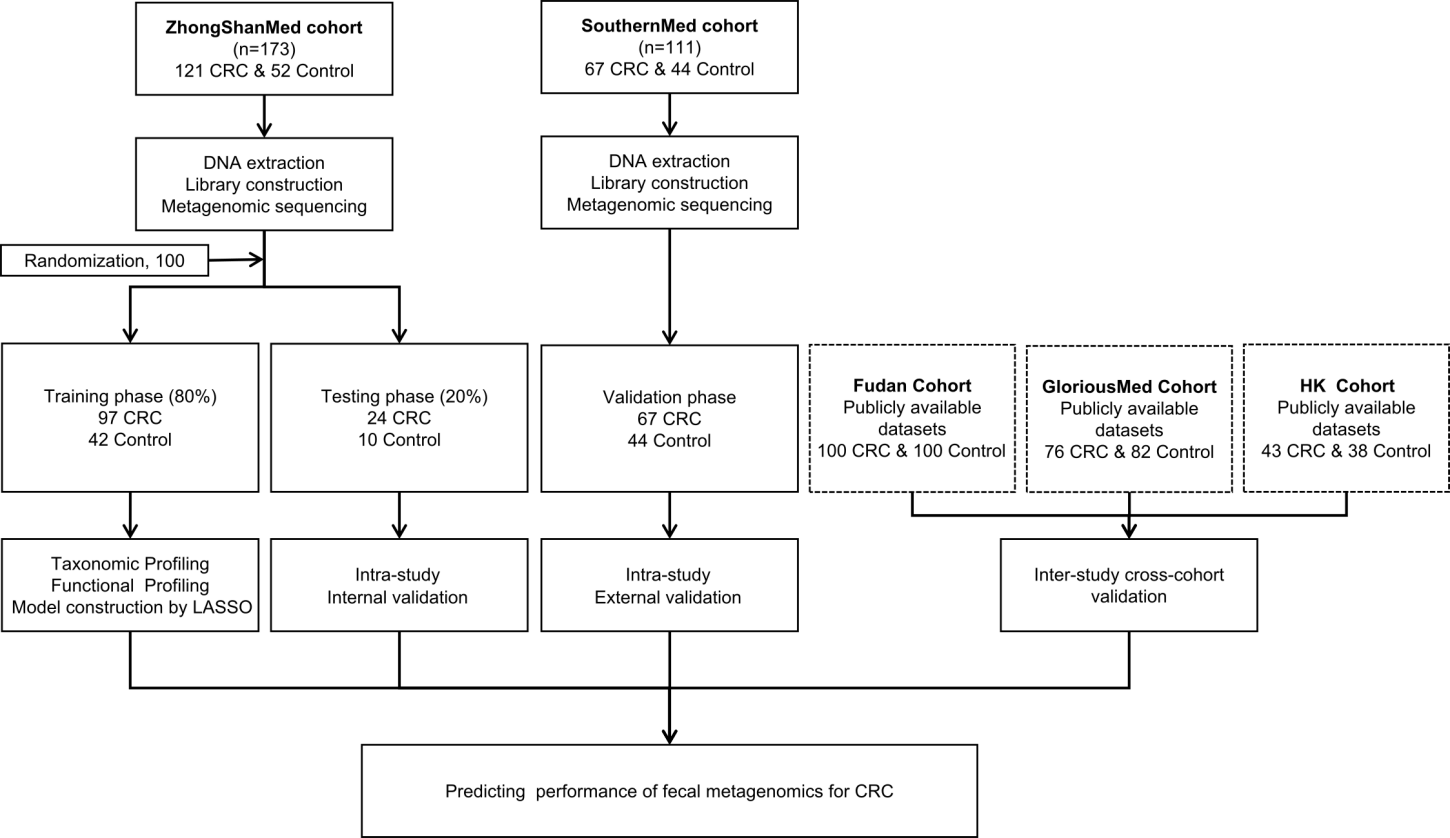
Study design and workflow diagram. A total of 284 cases and controls were included in this study. ZhongShanMed cohort was randomly divided into a training phase (accounted for 80%) and a testing phase (accounted for 20%) to identify the gut microbial markers. The strength of observed associations of microbial markers with CRC were further independently verified in SouthernMed cohort. The publicly available Fudan, GloriousMed, and HK datasets were retrieved from NCBI GEO database and EBI ENA browser and profiled for cross-platform validation of the LASSO predictive model.

In total, 188 CRCs and 96 healthy controls were recruited from two clinical sites. Clinical and pathological information for each cohort were recorded and summarized in **Supplementary Tables S1**-**S3**. The stool samples were subjected to microbial DNA isolation and subsequently sequenced by metagenomic sequencing. The ZhongShanMed subjects were randomly assigned to the training phase (80% on average: 97 CRC and 42 control subjects) and the testing phase (20% on average: 24 CRCs and 10 normal controls) in 100 runs. In the training and testing phases, we performed LASSO regression analysis to identify the microbial markers associated with CRC risk and to discriminate CRC and control. We subsequently used the classifier in the SouthernMed validation cohort with 67 CRC and 44 control subjects to assess its strength of CRC prediction. Furthermore, we obtained metagenomic sequencing data from Fudan, GloriousMed, and HK cohorts to further evaluate the predictive power of our LASSO regression model (**Figure 1**).

### 3.2 Gut microbiome shifts in CRC

We performed a stringent sequence similarity search of cleaned reads against the customized integrated microbiome database created from GTDB, PathSeq, and NCBI by BWA (**Supplementary Table S4**). An average of 99.69%, 0.07%, 0.03%, and 0.20% of total metagenomic reads were mapped against the bacteria, archaea, fungal, and virus genome sequences (**Supplementary Figure S1**), with bacterial taxa monopolizing the gut microbiota.

We subsequently investigated the phylum distribution in ZhongShanMed and SouthernMed cohorts, and the five most abundant phylum compositions included Bacteroidota (56.02%), Firmicutes (36.00%), Proteobacteria (2.98%), Verrucomicrobiota (0.84%), and Fusobacteriota (0.83%) (**Supplementary Figure 2A**). The Firmicutes/Bacteroidota (FB) ratio in CRC was not significantly different from that in control (p>0.05, **Supplementary Figure 2B**), and the alpha diversity of CRC microbiome were not significantly different from that of control (p>0.05, **Supplementary Figure 2C**).

**Figure 2:**
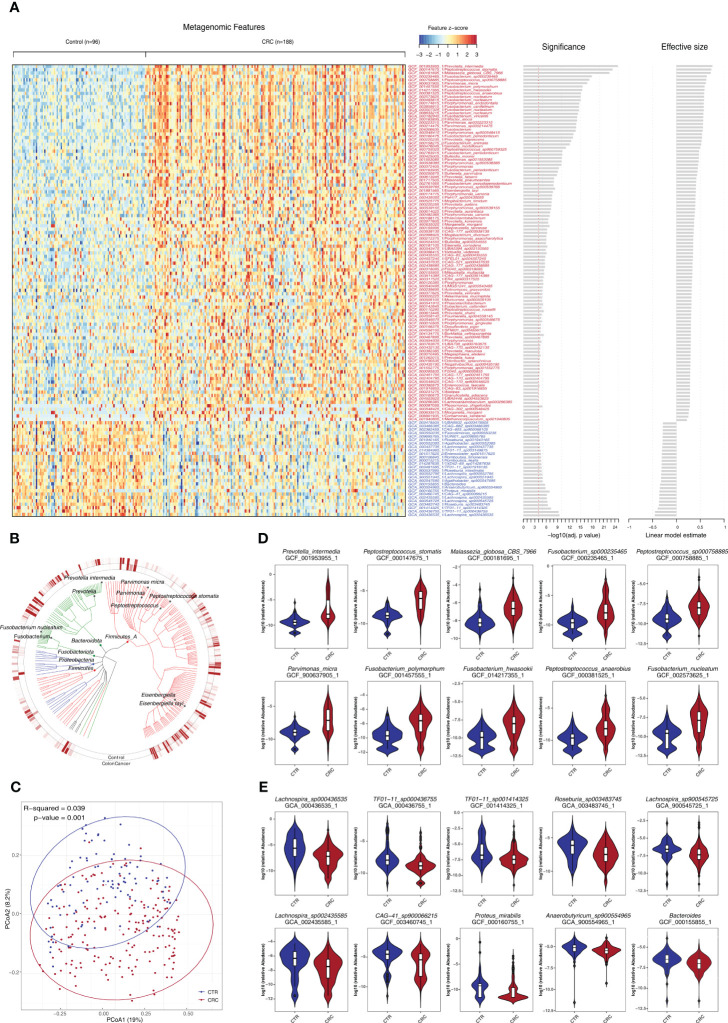
Identification of a set of gut microbes strongly associated with CRC. **(A)** Relative abundance of various species was assessed for significant elevation or depletion (two-sided Mann–Whitney U-test) in CRC compared to healthy controls (ZhongShanMed and SouthernMed combined). Levels of elevation or depletion for significant bacterial species are displayed as a heatmap (133 species shown, q<1E−4, also see **Supplementary Table S5** for details on a total of 481 species included, p<0.05). Species are ranked by effective size and direction of changes. **(B)** A total of top-ranked 227 deferentially abundant species (q<1E−3) are arranged as a phylogenetic tree and grouped according to the phyla of Firmicutes, Bacteroidota, Fusobacteriota, Proteobacteria, and Firmicutes. The colored inner and outer circles show average relative abundance of these species for healthy controls and CRC, respectively. **(C)** PCoA analysis was performed using the Bray–Curtis distances of arcsin(sqrt) transformed relative abundances for CRC (red) and controls (blue) and was assessed by adonis2. **(D, E)** Box plots of 10 most enriched **(D)** or 10 most depleted **(E)** species in CRCs. The y-axis for each box plot is logarithmic transformation of relative abundance of each species in CRC (red) and controls (blue).

We further examined the impact of potential confounding factors including age, gender, and BMI on CRC-related microbiome and performed a de-confounding analysis (**Supplementary Figures S3**, **S4**). Across the phyla of Proteobacteria, Fusobacteriota, Bacteroidota, and Firmicutes, 481 species were found significantly enriched or depleted in CRC (**Figures 2A, B**, **Supplementary Table S5**). Furthermore, the PCoA of these bacterial and fungal species supported the notion that CRC could be clearly distinguished from healthy controls (**Figure 2C**). Among them, 368 species were found to be enriched in CRC including certain well-established species such as *P. intermedia*, *Fusobacterium hwasookii*, *P. micra*, *P. asaccharolytica*, and *Enterococcus faecalis* (q<0.05, **Figure 2D**). The other 113 species were found to be depleted in CRC, most of which were not extensively studied, such as *Lachnospira* genus and *Proteus mirabilis* (q<0.05, **Figure 2E**).

We found that species exhibiting negative effect on butyrate production within the microbiome community were consistently elevated in CRCs such as *F. nucleatum*, *Fusobacterium periodonticum*, *Peptostreptococcus stomatis*, and *Megasphaera elsdenii*. In contrast, those species who are producing butyrate efficiently from carbohydrates, were consistently depleted, including *R. intestinalis*, *Roseburia hominis*, *Anaerostipes hadrus*, and *Faecalibacterium prausnitzii* (q<0.05, **Supplementary Table S5**) (37). In addition, we reported, for the first time, that several CRC-associated species, which have not been extensively studied, such as *Eisenbergiella tayiq*, *Odoribacter splanchnicus*, *Alistipes onderdonkii*, *Allisonella pneumosintes*, *CAG-83*, and *Malassezia globosa*, were significantly elevated in CRC (q<0.05). In contrast, *Romboutsia timonensis*, *Agathobacter faecis*, *R. intestinalis*, and *TF01-11* were significantly depleted (q<0.05, **Supplementary Table S5**).

### 3.3 Metabolic functional shifts in CRC

**Figure 3:**
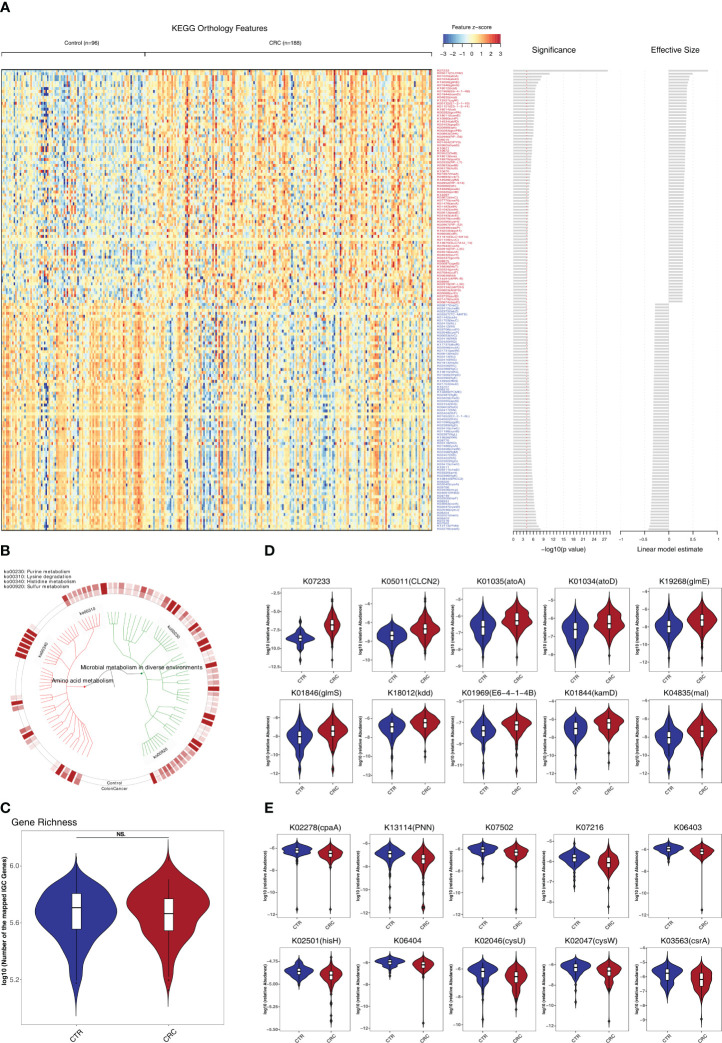
Identification of metabolomic alterations strongly associated with CRC. **(A)** Relative abundance of KEGG orthologous genes (KO genes) was assessed for significant elevation or depletion (two-sided Mann–Whitney U test) in CRC datasets compared to healthy controls (ZhongShanMed and SouthernMed combined). Significant changes in relative abundance of these KO genes are displayed as a heatmap (156 KO genes, q<1E–4, also see **Supplementary Table S6** for details on a total of 834 KO genes, p<0.05). KO genes are ranked by the significance of statistical analysis and direction of changes. **(B)** A total of 156 deferentially abundant KO genes are arranged in a functional hierarchy tree and grouped mainly as amino acid metabolism (red) and microbial metabolism in diverse environments (green). The colored inner and outer circles show average relative abundance for healthy controls and CRC, respectively. **(C)** Gene richness comparison between CRC (red) and controls (blue) was performed for both cohorts combined. **(D, E)** Box plots show the relative abundances of 10 most enriched **(D)** and 10 most depleted **(E)** KO genes in CRC. The y-axis for each box plot is logarithmic transformation of relative abundance of each KO gene.

To investigate the functional and metabolic changes in gut microbial communities, the clean reads were first aligned to the IGC to obtain KEGG modules. A total of 577 and 257 KO genes were significantly elevated and depleted, respectively, in CRC versus healthy controls (**Supplementary Table S6**; **Figures 3A, B**). In the phenylalanine metabolism, *paaE* (K02613) was significantly elevated. In the tyrosine and tryptophan biosynthesis, *hisC* (K00817), *trpD* (K00766) and *trpA* (K01695) were significantly depleted (q<0.05). Notably, the genes involved in lysine degradation were significantly elevated, such as *kamD* (K01844), *atoA* (K01035), *atoD* (K01034), *kdd* (K18012), *kamE* (K18011), *kal* (K18014), *dapD* (K00674), *kce* (K18013), and *lysA* (K01586) (q<0.05, **Figures 3D, E**). Notably, the gene richness in healthy controls was not significantly different from that of CRC based on IGC annotation (p>0.05, **Figure 3C**). We further performed the community-flux balance analysis and found that the secretion rate of butyrate acid were significantly reduced in CRC than that in control cohort (p<0.05, **Supplementary Figure S5**).

### 3.4 Predicting performance of fecal microbiome to classify CRC and control

**Figure 4:**
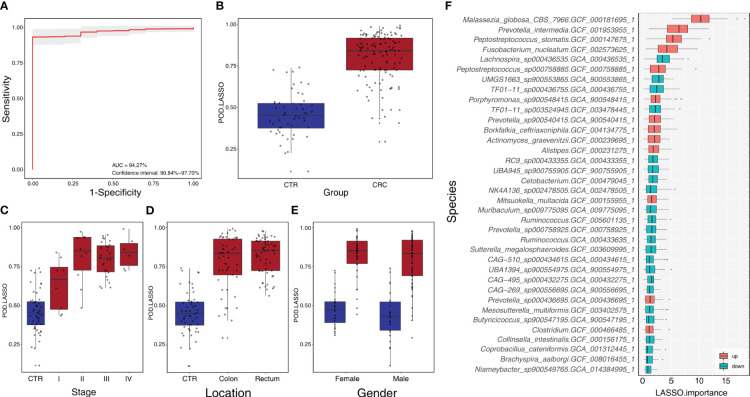
Identification of microbial markers associated with CRC by LASSO modeling in the training phase using ZhongShanMed cohort. **(A)** The POD-based AUROC value was evaluated between CRC and Control in the training set. Gray area denotes ±1 standard deviation for AUROC value. POD index was calculated using L1-regularized (LASSO) logistic regression model. **(B)** POD-score distribution across non-cancer individuals and cancer patients (CTR, n=52; CRC, n=121). The box plot denotes 25th–75th percentiles, and the central mark indicates the median; p-value is calculated by a two-sided unpaired Mann–Whitney test. **(C)** POD-score distribution across non-cancer individuals and cancer patients stratified by stage (Stage I, n=8; Stage II, n=10; Stage III, n=27; and Stage IV, n=8). **(D)** POD-score distribution across non-cancer individuals and cancer patients stratified by tumor anatomic location (CTR, n=52; CRC, colon, n=53; rectum, n=58). **(E)** POD-score distribution across non-cancer individuals and cancer patients stratified by gender (female: CTR, n=25, CRC, n=77; male: CTR, n=27, CRC, n==44). **(F)** Metagenomic markers for detecting patients with CRC from healthy controls identified from LASSO classifiers. The boxes represent 25th–75th percentiles with black lines indicating the median, whiskers extending to the maximum and minimum values within 1.5× the interquartile range, and dots denoting outliers. The boxes are marked in red for overrepresentation and in blue for underrepresentation (p<0.05) in CRC patients compared to the healthy controls.

**Figure 5:**
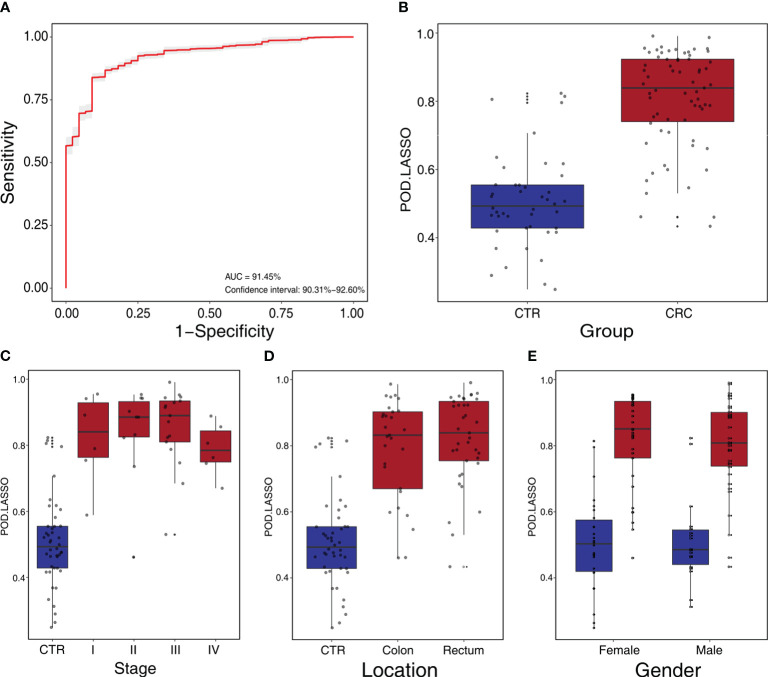
Independent validation and diagnostic performance of fecal microbial markers for CRC in SouthernMed external validation cohort. **(A)** The POD-based AUROC value between CRC and control in the validation set. Gray area denotes ±1 standard deviation for AUROC. **(B)** The POD values were compared between CRC and control in the independent validation phase (CTR, n=44; CRC, n=67). The box denotes 25th–75th percentiles, and the central mark indicates the median; p-value is calculated by two-sided unpaired Mann–Whitney test. **(C)** POD-score distribution across non-cancer individuals and cancer patients stratified by stage (CTR, n= 44; CRC, Stage I, n=6; Stage II, n=10; Stage III, n=17; and Stage IV, n=6). **(D)** POD-score distribution across non-cancer individuals and cancer patients stratified by tumor anatomic location (CTR, n= 44; CRC, colon, n=29; rectum, n=37). **(E)** POD-score distribution across non-cancer individuals and cancer patients stratified gender (female, CTR, n=26, CRC, n=33; male, CTR, n=18, CRC, n=34).

We constructed a LASSO classifier and used the AUROC to evaluate the performance of the classifier from the training set on the held-out testing set. Because the held-out testing set samples were not used for GLMnet training and tuning, the estimates represent unbiased measures of classification. Furthermore, the 100 independent training and test sets avoided the training-set bias and achieved the minimization of optimistic estimates. The control-versus-CRC binomial classifier was trained on the discovery datasets and then assessed on the independent validation cohort. The probabilities from 100 models were averaged to calculate a single score and used for AUROC estimation. The LASSO classifier achieved an AUROC of 94.27% (CI: 90.84%–97.70%) for ZhongShanMed (**Figure 4**) and an AUROC of 91.45% (CI: 90.31%–92.60%) for SouthernMed (**Figure 5**). At a POD cutoff of 0.642, the binomial classifier could detect stage I/II and III/IV CRC with respective sensitivities at 66.7% and 90.9% and a specificity at 90.4% in ZhongShanMed cohort. Sensitivities in SouthernMed cohort were significantly increased, reaching 87.5% (I/II) and 95.7% (III/IV) with a slightly lower specificity at 88.6% (**Supplementary Table S7**).

We also adopted RF and SVM methods to construct different classifiers to predict CRC, and the estimated AUROCs was slightly lower than those from the LASSO model. In summary, the RF model achieved AUROC of 84.14% (CI: 76.44%–91.84%) in ZhongShanMed and 82.09% (CI: 79.94%–84.23%) in SouthernMed (**Supplementary Figures S6**, **S7**), whereas the SVM model achieved an AUROC of 95.50% (CI: 92.38%–98.63%) in ZhongShanMed and 82.08% (CI: 80.12%–84.05%) in SouthernMed (**Supplementary Figures S8**, **S9**).

Finally, the classification power of the LASSO classifier was evaluated for 449 metagenomes from three published studies (7, 8, 31). The POD scores were calculated by the LASSO model trained on the ZhongShanMed datasets. As shown in **Supplementary Figure S10**, the AUROC values were estimated at 79.99% (CI: 78.21%–81.76%), 78.85% (CI: 77.68%–80.01%), and 82.10% (CI: 80.68%–83.52%) for the whole group, the younger group (age≤50), and the elderly group (age>50), respectively, in the Fudan cohort. In addition, the AUROC estimates were similar for GloriousMed cohort at 80.35% (CI: 78.92%–81.78%) and higher for HK cohort at 91.73% (CI: 90.61%–92.85%) (**Supplementary Figure S11**).

### 3.5 Common microbial species biomarkers across various geographical and ethnic cohorts

**Figure 6:**
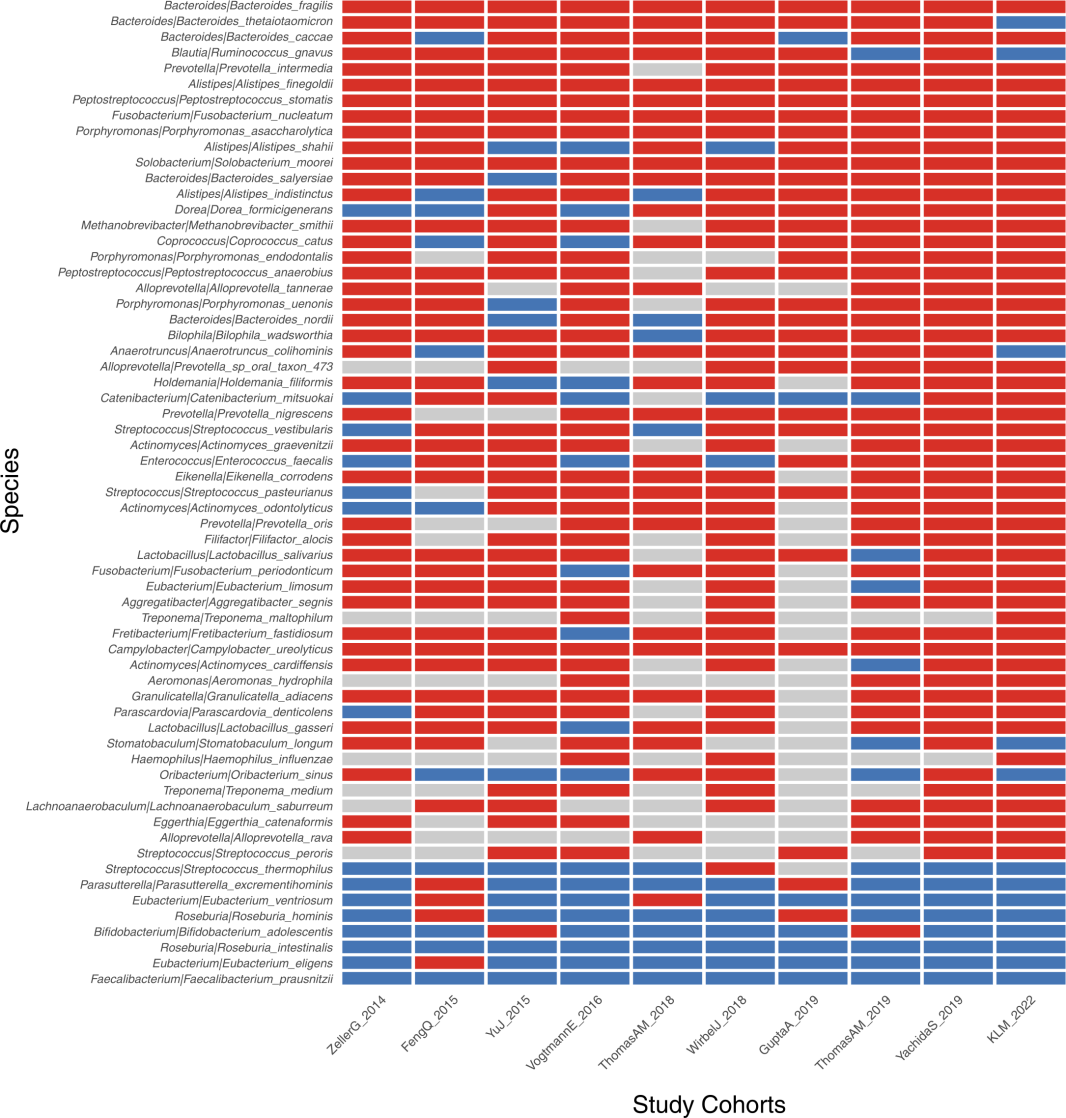
Identification of CRC-associated bacterial markers across 10 validation cohorts. The enriched, depleted, and unchanged markers are indicated by red, blue, and gray boxes, respectively. Only markers with significance of p<0.05 across all 10 cohorts are demonstrated.

We further performed the meta-analysis of a total of 10 colorectal cancer metagenomic datasets including ours to identify the cross-cohort microbial signatures. The result showed that 73 and 9 (*p*<0.05, **Supplementary Table S9**) species/strains were coherently enriched or depleted, respectively, in CRC versus control subjects, despite the heterogeneity of effective size across datasets (**Figure 6**). In particular, *Bacteroides fragilis*, *P. micra*, *Alistipes finegoldii*, *P. stomatis*, *F. nucleatum*, *P. asaccharolytica*, *Gemella morbilorum*, *Solobacterium moorei*, and *Campylobacter ureolyticus* were found to be enriched in every study analyzed. However, only two species, *R. intestinalis* and *F. prausnitzii*, were found to be depleted strictly across all metagenomic studies.

## 4 Discussion

In our current multi-center study, we conducted a large-scale next-generation sequencing of 284 metagenomes from two independent hospital-based cohorts and a comprehensive metagenome-wide association analysis to evaluate shifts in microbial compositions and metabolic functions in gut microbiota of CRC, in which the species dysbiosis may provide a microcosm favorable to oncogenesis. Our validation approach across wide-ranging geographical and ethnic cohorts including our own confirmed a series of well-recognized CRC-enriched bacterial species, such as *F. nucleatum*, *B. fragilis*, *P. micra*, *S. moorei*, and *P. stomatis* (8, 38). In addition, we have identified significant taxonomic alterations in some interesting and less-known species including *A. finegoldii*, *P. asaccharolytica*, *R. intestinalis*, and *F. prausnitzii* whose roles in CRC are worth further investigating (39). *Alistipes* is an emerging genus in the Bacteroides phylum implicated in pathogenesis of CRC and mental disorders. As a Gram-negative anaerobic rod and infectious pathogen, *P. asaccharolytica* has only recently been identified as a CRC-enriched bacterial species in gut microbiome (39). Among CRC-depleted species, *R. intestinalis* and *F. prausnitzii* are butyrate-producing bacteria associated with healthy gut symbiosis and certain metabolic diseases such as diabetes (40). Butyrate is considered a critical energy source for self-renewing healthy intestinal epithelial cells and has anti-inflammatory and anti-tumor effects, whereas its reduction may lead to cancer (41, 42). In fact, our pooled functional analysis of the metagenomic data revealed a likely scenario of competition for reduced butyrate biosynthesis, which may be partially compensated with an inefficient lysine–butyrate pathway due to increased abundance of CRC-enriched bacteria such as *F. nucleatum*. The above-mentioned CRC-associated bacterial species were also revealed by meta-analysis of multiple global cohorts across different ethnicities and geographical regions (39). Notably, *Clostridium symbiosum*, a consistent CRC-enriched species, is missing from our analysis possibly due to cohort heterogeneity caused by different ethnic background, dietary preference, and other environmental factors. Nevertheless, the panel of these universal bacterial markers can be robust across different populations in diagnosing CRC (39) and potentially used globally for the accurate screening of this malignant disease.

By modeling the changing profiles of these microbial species and subspecies, we used three classification methods to discriminate CRC metagenomes from those of healthy individuals. RF is a powerful machine learning model that has been widely attempted for distinguishing CRC from control in metagenomic studies (8, 34, 43). It has achieved AUROC values of 0.83–0.96 in discovery cohorts of Austrians, Chinese, and Japanese with fixed panels of microbial markers, but the accuracy has not been validated within independent cohorts using the same RF classifier. Our RF regression analysis achieved a similar prediction accuracy of AUROC values of 84.14% and 82.09% in our ZhongShanMed and SouthernMed cohorts, respectively. The CRC-prediction panel consists of 22 bacterial species including some major CRC-associated bacteria such as *B. fragilis*, *R. intestinalis*, and *Clostridium*. However, *F. nucleatum* was not on the panel. In contrast, SVM achieved a much higher AUROC value of 95.50% in ZhongShanMed cohort with a unique panel of 28 bacterial species. However, AUROC value was drastically reduced to 82.08% in SouthernMed cohort as the POD scores for various metagenomes were significantly spread out in both CRC and control groups. In our study, LASSO achieved robust predicting performance in both ZhongShanMed and SouthernMed cohorts originated in the same city of Guangzhou in the southern part of China. The classifier also achieved a good performance in Fudan (AUROC, 79.99%) and GloriousMed (AUROC, 80.35%) cohorts whose samples were collected mainly from the eastern region of China. Furthermore, the classifier achieved a high accuracy of CRC prediction in HK cohort (AUROC, 91.73%), as Hong Kong is a city in the vicinity of Guangzhou. Certain bacterial species with the top-ranked LASSO importance on the 32-species panel included *Malassezia globosa*, also one of the fungal markers used for CRC detection (10), and *P. intermedia*, *P. stomatis*, and *F. nucleatum*, three notoriously enriched bacterial species often used to predict CRC. Higher AUROC values achieved in the current study by LASSO binomial classifier help increase the overall sensitivity of CRC detection to 87% (78/90) with specificity at 90% (86/96) in the combined cohorts of ZhonShanMed and SouthernMed. In particular, the classifier can accurately discriminate patients with early-stage cancers (I/II) from healthy individuals at sensitivity of 76% (26/34), higher than the value from a serum-based mSEPT9 test at 45.7%–64.3% (44), comparable to a reported value for FIT at 81.6% (45) and a multitarget stool DNA test at 80%–84% (46), but lower than 87% achieved by a single-target fecal mSDC2 test (47). These findings suggested that fecal microbiota-based biomarkers were potentially feasible in predicting CRC risks with high accuracy in populations from proximal geographical regions. The gut CRC-related microbiome signatures can be delineated by appropriate modeling and may be used as good risk assessment and personalized screening strategies to improve the detection capability of at-risk populations and to aid diagnosis by colonoscopy screening.

It is increasingly evident that dysbiosis of the intestinal microbiota is associated with higher risk of CRC onset. A large number of clinical trials have been developed to apply our recent research findings for the microbiome-based early detection and screening of CRC. CRCbiome is a large-scale ongoing prospective sub-study within a randomized CRC screening trial in Norway expected to be completed in 2030 (https://ClinicalTrials.gov; NCT01538550) (48). By analyzing metagenomic sequencing data of fecal samples from participants who have been tested positive by FIT, the trial aims to identify microbiome markers and develop CRC classifier for early detection of advanced colorectal lesions. Another interesting prospective cross-sectional study in Hong Kong, which is actively recruiting trial participants with average risk of CRC, employs a panel of four bacterial gene markers called “M3” to evaluate the diagnostic accuracy for precancerous lesions and advanced neoplasia using quantitative PCR method (NCT05405673) (49). Notably, the panel will be tested head to head with FIT to compare their overall sensitivity for the detection of CRC and adenomas. In addition to the evaluation of microbiome by metagenomic sequencing and qPCR, 16S rRNA amplicons sequencing has also been utilized in an active clinical trial to assess the relationship of gut microbiota and specific polyp types including serrated, hyperplastic, and cancerous polyps (NCT03297996). Furthermore, as an extension of recent published research work by Chen and colleagues (11), a clinical trial started most recently in April will use liquid chromatography-mass spectrometry (LC-MS) to test a panel of gut-microbiome-related serum metabolites for screening of advanced adenomas and CRC. Evidently, these trial outcomes have critical implications in microbiome-based early detection and risk prediction of CRC in the future. On the one hand, microbiome or microbiome-related biomarkers, combined with methylation markers, FIT, and other methods, have the potential to improve the sensitivity, specificity, and accuracy of CRC detection and screening. On the other hand, the microbiome composition can inform risk prediction model of CRC to help determine which patients are at highest risk of having adenomas or cancer (NCT04185779).

## 5 Limitations

Certain limitations are associated with our present investigation. First, we employed MGISEQ-2000 sequencing platform, which is different from Illumina HiSeq or NextSeq used in most other studies and validation cohorts from Fudan, GloriousMed, and HK. Although the former has been reported to exhibit cross-platform consistency, accuracy, and reproducibility compared with the latter, approximately 2.02%–3.25% of genes were found to have significant differences in relative abundance between the two platforms (50). Hence, it is possible that the different platforms affect the outcome of relative abundance of a low proportion of genes and CRC-associated taxonomic profiling. However, since the identities of these genes were not revealed, the definitive effects on the relative abundance of fecal metagenomics were difficult to evaluate and quantify. Nevertheless, our study of CRC-associated species common across all 10 studies indeed support the notion that MGI platform is applicable to metagenomic studies. Second, shifts of POD scores in control subjects towards those of CRC were observed in Fudan and GloriousMed cohorts, lowering AUROC values for these two cohorts. We speculated that the relative lower AUROC values of public validation datasets were mainly caused by the fluctuation of microbial community structure attributed from different life and dietary habits but not the slightly platform-based differences in relative abundance. A reasonable explanation is that the initial LASSO modeling was not able to capture the exact and optimal bacterial features in healthy individuals, indicating that additional control subjects in the discovery cohort may be needed for metagenomic sequencing and regression analysis to obtain an improved outcome for CRC prediction for cohorts residing in wider geographical regions. However, it is indeed difficult to define what is the canonical microbial community structure of large populations. A recent Dutch study recruited 2,937 healthy subjects for metagenomic sequencing to characterize the landscape of gut microbes shaped up by a host of factors in genetics, exposome, lifestyle, and diet (51). Third, a follow-up validation in a prospective case–control cohort either by metagenomic sequencing to test the performance of LASSO binomial classifier or quantitative PCR method by selecting a panel of bacterial markers is still lacking in the current study.

## 6 Conclusions

Overall, our multi-center study of fecal metagenomes reveals in detail a common state of gut microbial dysbiosis in CRC populations across different ethnicities. We identified fecal microbial markers for distinguishing CRC from healthy controls and validated the strength of observed associations based on LASSO/RF/SVM classification models. We demonstrate that panels of 20–40 fecal microbiota-based microbial biomarkers have a robust sensitivity in detecting CRC with a suitable machine learning model at work. Although additional clinical validations and explicit experimental evidence underlying microbiome dysbiosis are much needed, our present study may aid the clinical transformation of microbiota-based strategies into precision screening and diagnosis in real-world practice.

## Data availability statement

All relevant data is contained within the article. The original contributions presented in the study are included in the article/**Supplementary Material**, further inquires can be directed to the corresponding author.

## Ethics statement

This study was performed in accordance with the principles of the Helsinki Declaration and approved by the Institutional Review Board of the Sixth Affiliated Hospital of Sun Yat-sen University and Nanfang Hospital. All participants have acknowledged and signed informed consent.

## Author contributions

XSW and HZ performed conceptual design, obtained funding, and supervised the study. SL and YW collected samples and gathered clinical information. RZ, ZT, and XSW performed metagenomic sequencing experiments and acquired data. XDW performed sequencing data modeling and statistical analyses all at Creative Biosciences. XDW, ZT, and XSW drafted and revised the manuscript. HZ and SL performed critical review of the manuscript. RZ provided administrative, technical, and material support. All authors contributed to the article and approved the submitted version.
