# Supplementary material for: Taxonomic and functional profiling of fecal metagenomes for the early detection of colorectal cancer
Source: Front Oncol. 2023 Aug 3;13:1218056. doi: 10.3389/fonc.2023.1218056 (PMC10436198; doi:10.3389/fonc.2023.1218056)
Supplement: Supplementary file 1 [file Image_1.pdf]

Supplementary Materials

Supplementary Figures and Figure Legends

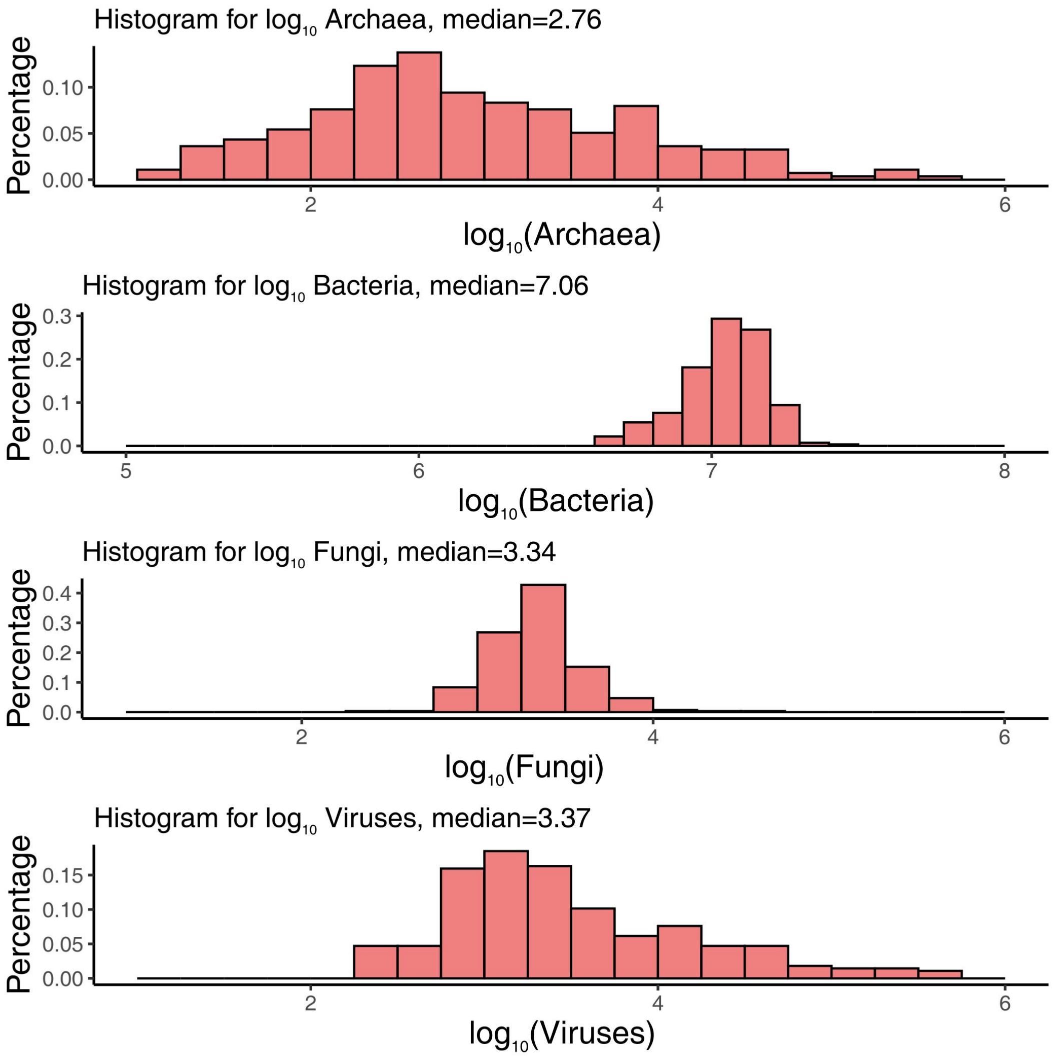

Supplementary Figure 1. Sequence read counts of archaea (A), bacteria (B), fungi (C) and viruses (D) in both ZhongShanMed and SouthernMed cohorts combined.

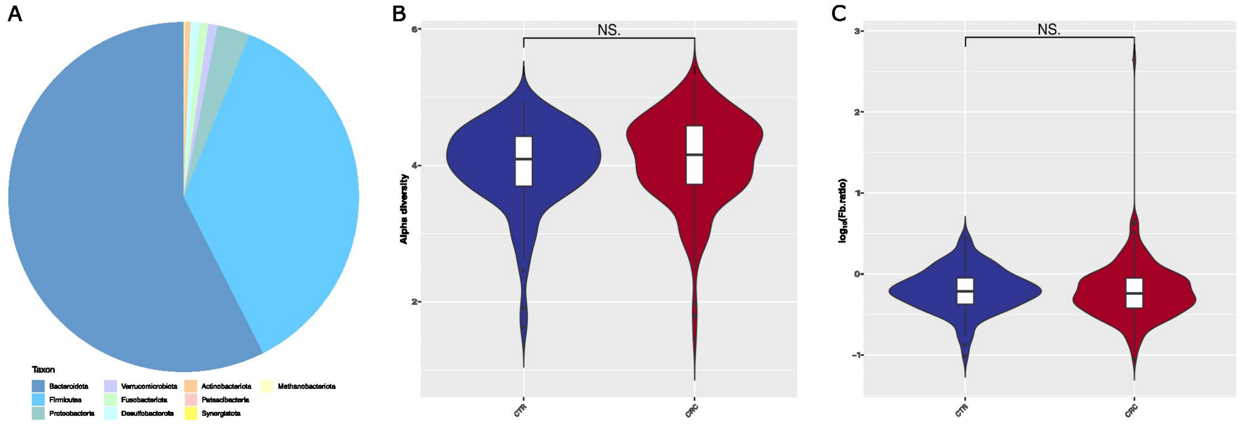

Supplementary Figure 2. The phylogenic distribution of fecal microbiota in CRCs and controls. (A) Phyla distribution in ZhongShanMed and SouthernMed cohorts combined. (B) Comparison of alpha diversity of fecal microbiota between CRC and control in two cohorts combined. (C) F/B ratio of fecal microbiota in between CRC and control in two cohorts combined.

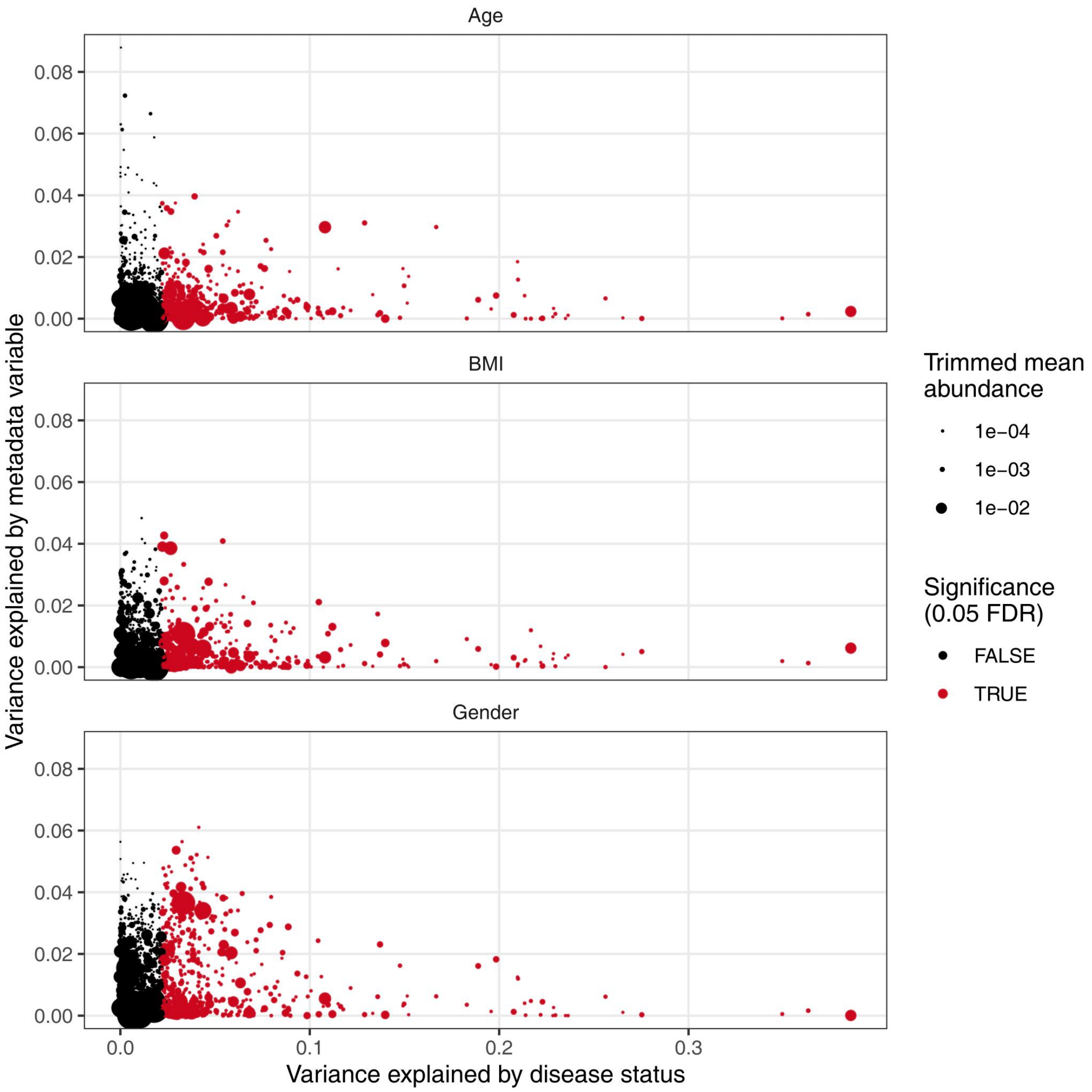

Supplementary Figure 3. De-confounding analysis of CRC-association of individual species regarding three potential confounding factors including age (top panel), BMI (middle panel), and gender (bottom panel). Variance explained by disease stage (CRC or control) was plotted against variance explained by different putative confounding factors for individual species. Each significant species is represented by a red dot proportional in size to its relative abundance.

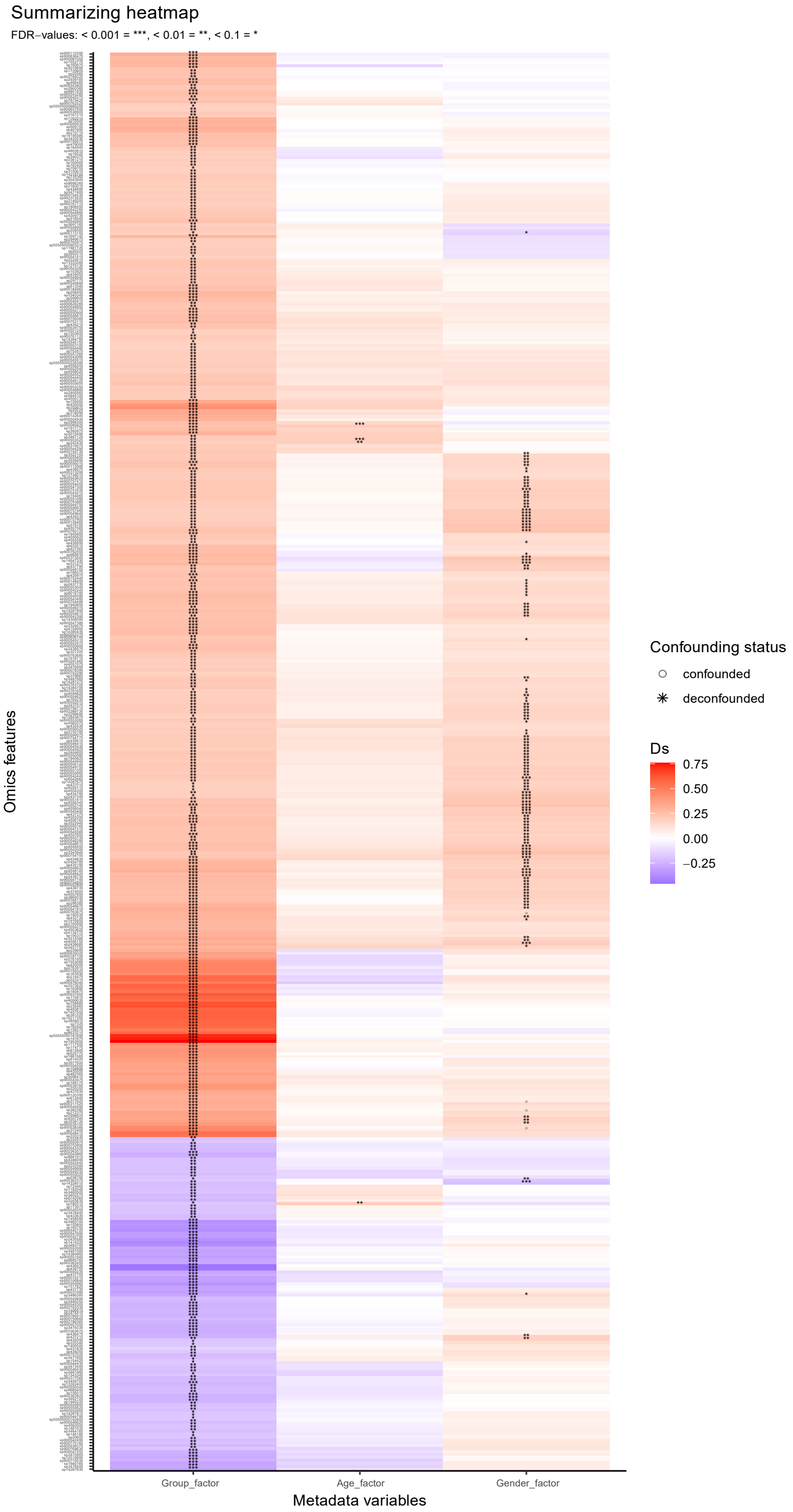

Supplementary Figure 4. Heatmap of individual CRC-associated species significantly corrected after de-confounding analysis of putative confounder factors of group (left panel), age (middle panel), and gender (right panel).

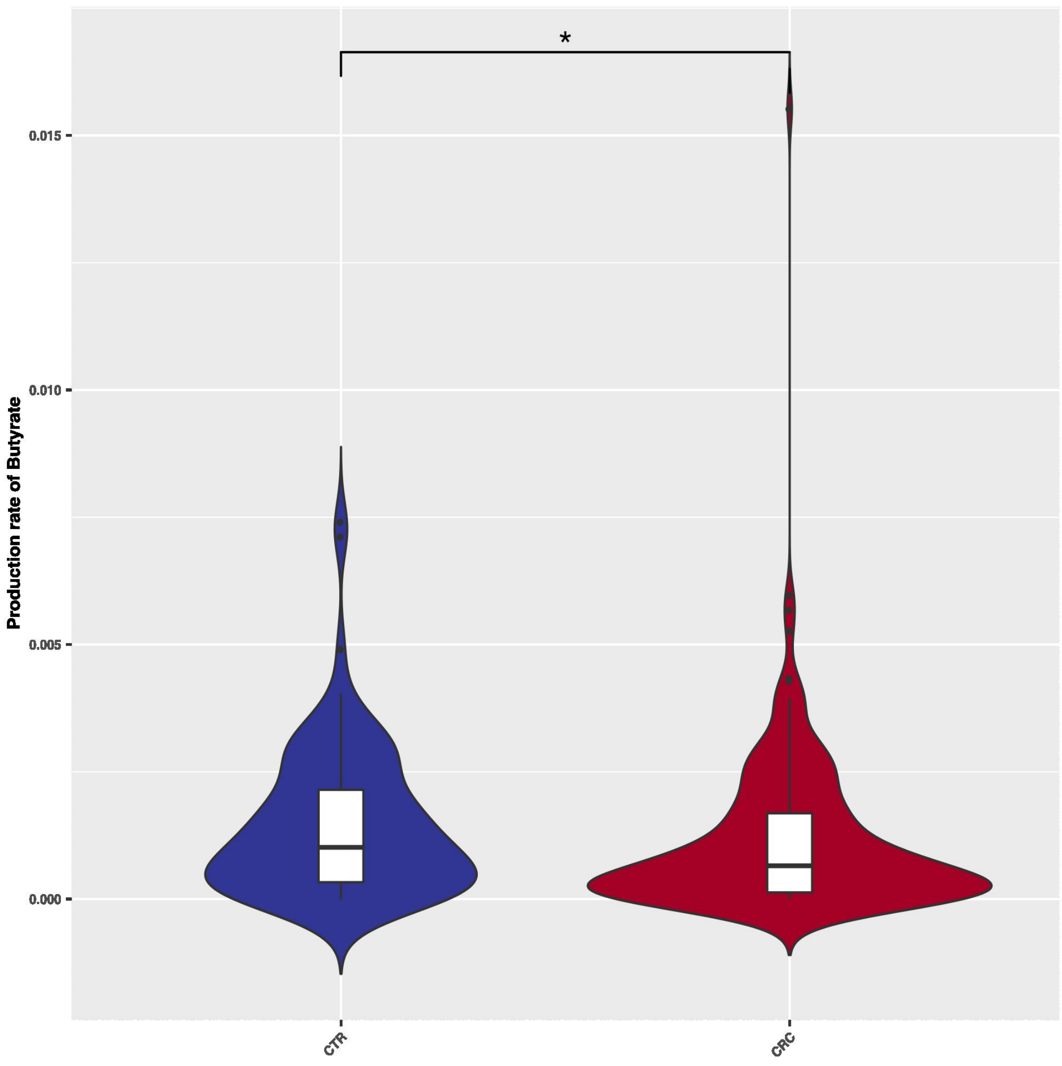

Supplementary Figure 5. Comparison of secretion rate of butyrate acid between CRC and control in two cohorts combined estimated based on the community-flux balance analysis.

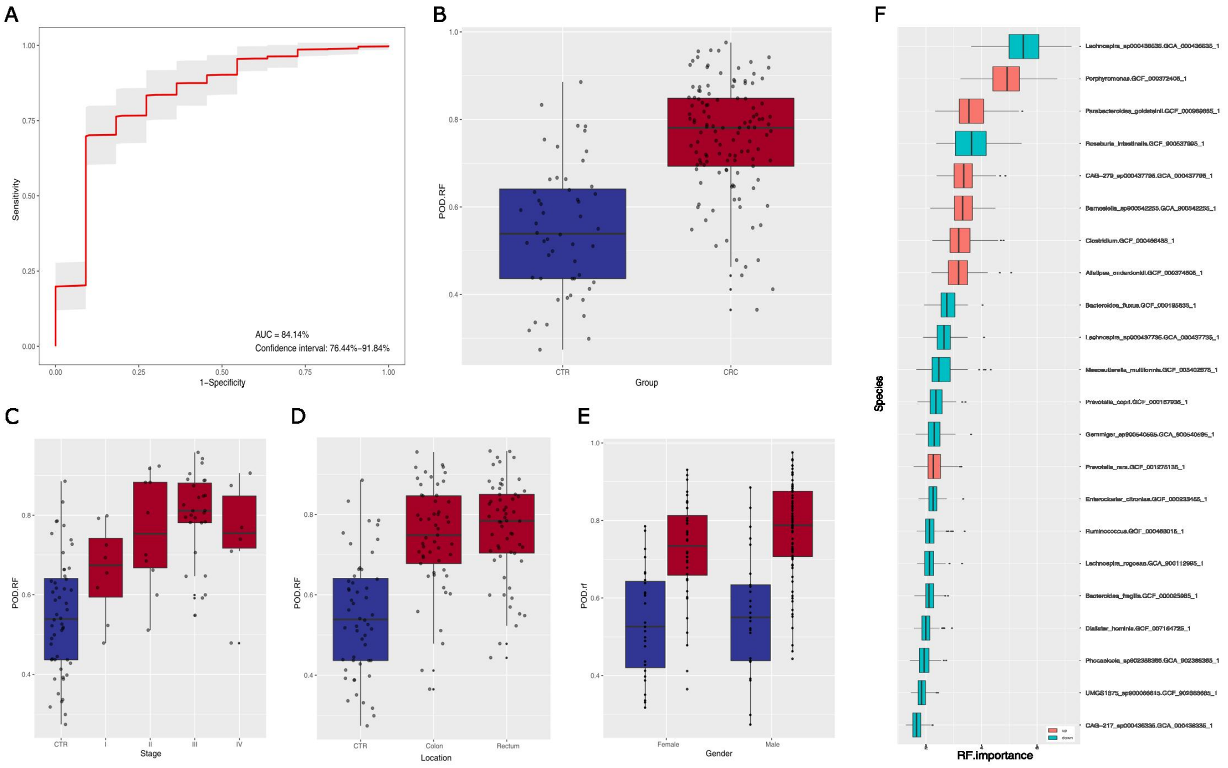

Supplementary Figure 6. Identification of microbial markers associated with CRC by RF modeling in the training phase using ZhongShanMed cohort. (A) The POD-based AUROC value between CRC and Control in the training set. Grey area denotes ±1 standard deviation for AUROC value. The probability of disease (POD) index was calculated using RF model. (B) POD-score distribution across non-cancer individuals and cancer patients (CTR, n=52; CRC, n=121). The box plot denotes 25th-75th percentiles, and the central mark indicates the median; p value is calculated by two-sided unpaired Mann-Whitney test. (C) POD-score distribution across non-cancer individuals and cancer patients stratified by stage (CTR, n=52; CRC, Stage I, n=8; Stage II, n=10; Stage III, n=27; Stage IV, n=8). (D) POD-score distribution across non-cancer individuals and cancer patients stratified by the anatomic locations (CTR, n=52; CRC, colon, n=53; rectum, n=59). (E) POD-score distribution across non-cancer individuals and cancer patients stratified patient gender (female, CTR, n=25, CRC, n=77; male, CTR, n=27, CRC, n=44). (F) Metagenomic markers for detecting patients with CRC from healthy controls identified from RF classifiers. The boxes represent 25th-75th percentiles, black lines indicate the median, whiskers extend to the maximum and minimum values within 1.5X the interquartile range and dots indicate outliers. The boxes are marked in red for overrepresentation, in blue for underrepresentation ($p<0.05$, Mann-Whitney U test) in CRC patients compared to the healthy controls.

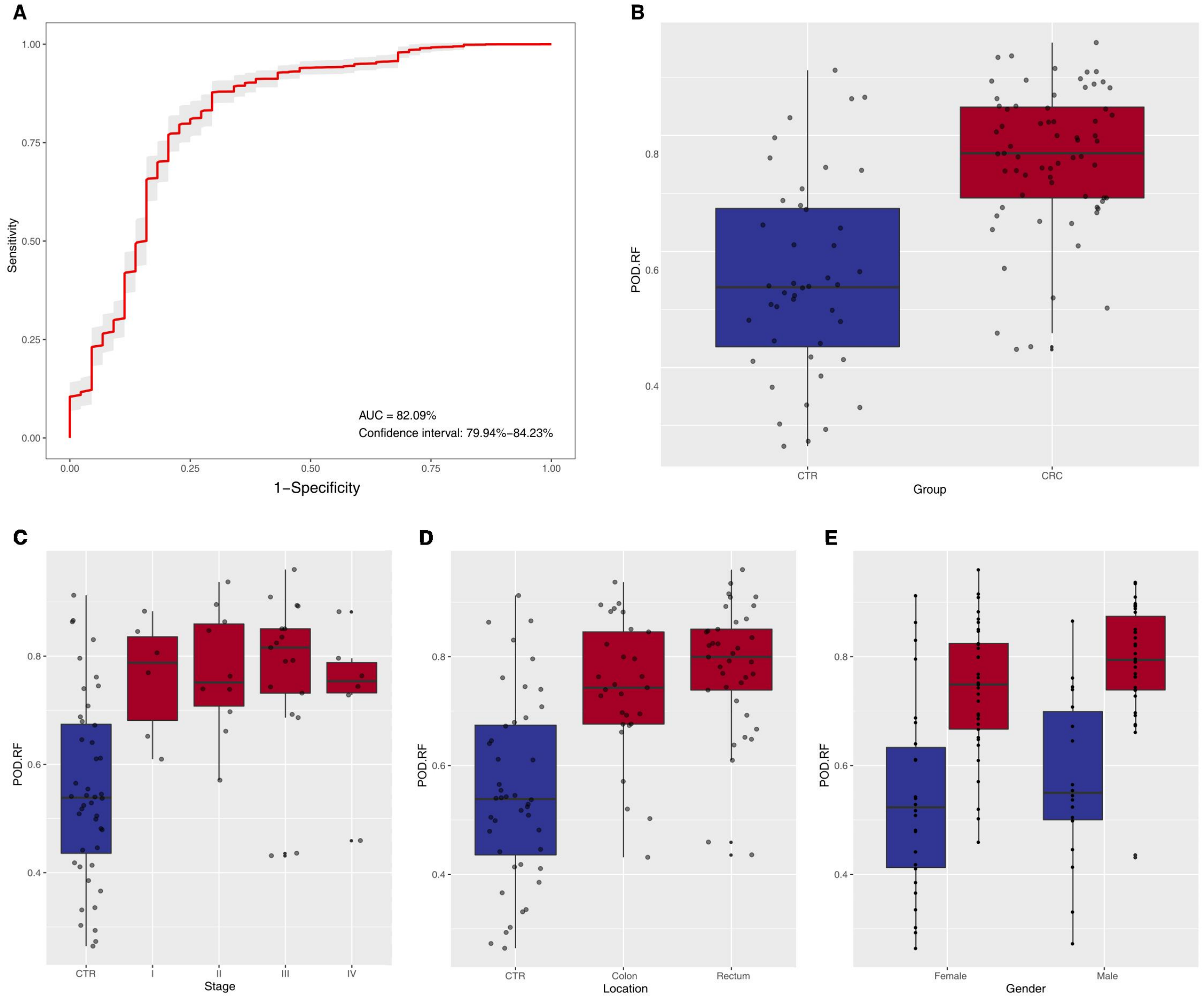

Supplementary Figure 7. Independent validation and diagnostic performance by RF modeling for CRC in external SouthernMed cohort. (A) The POD-based AUROC value between CRC and Control in the validation set, grey area denote ±1 standard deviation for AUROC value. (B) POD-score distribution across non-cancer individuals and cancer patients (CTR, n=44; CRC, n=67) in the independent validation phase. The box denotes 25th-75th percentiles, and the central mark indicates the median; p value is calculated by two-sided unpaired Mann-Whitney test. (C) POD-score distribution across non-cancer individuals and cancer patients stratified by stage (CTR, n=44; CRC, Stage I, n=6; Stage II, n=10; Stage III, n=17; Stage IV, n=6). (D) POD-score distribution across non-cancer individuals and cancer patients stratified by the anatomic locations (CTR, n=44; CRC, colon, n=29; rectum, n=37). (E) POD-score distribution across non-cancer individuals and cancer patients stratified by gender (female, CTR, n=26, CRC, n=33; male, CTR, n= 18, CRC, n=34).

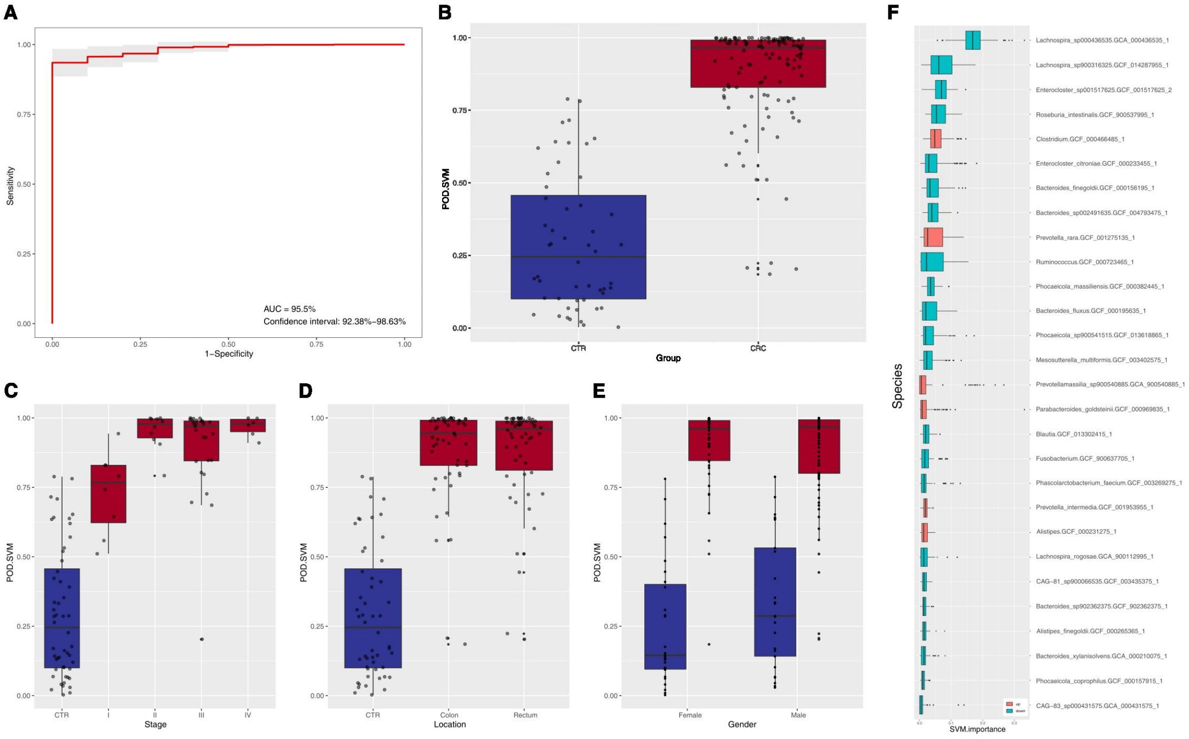

Supplementary Figure 8. Identification of microbial markers associated with CRC by SVM in the training phase using ZhongShanMed cohort. (A) The POD-based AUROC value between CRC and Control in the training set. Grey area denotes ±1 standard deviation for AUROC value. POD index was calculated using SVM model. (B) POD-score distribution across non-cancer individuals and cancer patients (CTR, n=52; CRC, n=121). The box plot denotes 25th-75th percentiles, and the central mark indicates the median; p value is calculated by two-sided unpaired Mann-Whitney test. (C) POD-score distribution across non-cancer individuals and cancer patients stratified by stage (CTR, n=52; CRC, Stage I, n=8; Stage II, n=10; Stage III, n=27; Stage IV, n=8). (D) POD-score distribution across non-cancer individuals and cancer patients stratified by the anatomic locations (CTR, n=52; CRC, colon, n=53; rectum, n=59). (E) POD-score distribution across non-cancer individuals and cancer patients stratified patient gender (female, CTR, n=25, CRC, n=77; male, CTR, n=27, CRC, n=44). (F) Metagenomic markers for detecting patients with CRC from healthy controls identified from SVM classifiers. The boxes represent 25th-75th percentiles, black lines indicate the median, whiskers extend to the maximum and minimum values within 1.5X the interquartile range and dots indicate outliers. The boxes are marked in red for overrepresentation, in blue for underrepresentation ($p<0.05$, Mann-Whitney U test) in CRC patients compared to the healthy controls.

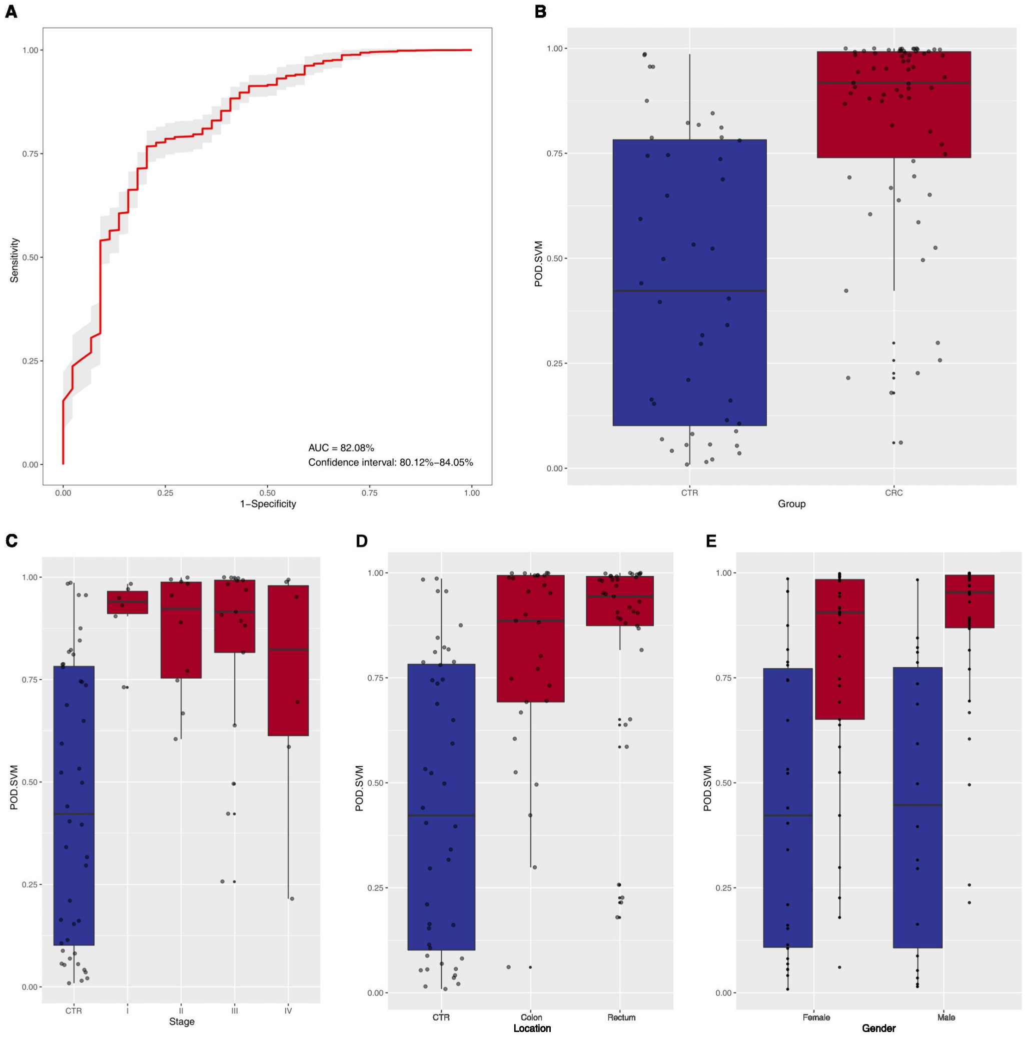

Supplementary Figure 9. Independent validation and diagnostic performance by SVM for CRC in external SouthernMed cohort. (A) The POD-based AUROC value between CRC and Control in the validation set, Grey area denote ±1 standard deviation for AUROC value. (B) POD-score distribution across non-cancer individuals and cancer patients (CTR, n=44; CRC, n=67) in the independent validation phase. The box denotes 25th-75th percentiles, and the central mark indicates the median; p value is calculated by two-sided unpaired Mann-Whitney test. (C) POD-score distribution across non-cancer individuals and cancer patients stratified by stage (CTR, n=44; CRC, Stage I, n=6; Stage II, n=10; Stage III, n=17; Stage IV, n=6). (D) POD-score distribution across non-cancer individuals and cancer patients stratified by anatomic locations (CTR, n=44; CRC, colon, n=29; rectum, n=37). (E) POD-score distribution across non-cancer individuals and cancer patients stratified by gender (female, CTR, n=26, CRC, n=33; male, CTR, n=18, CRC, n=34).

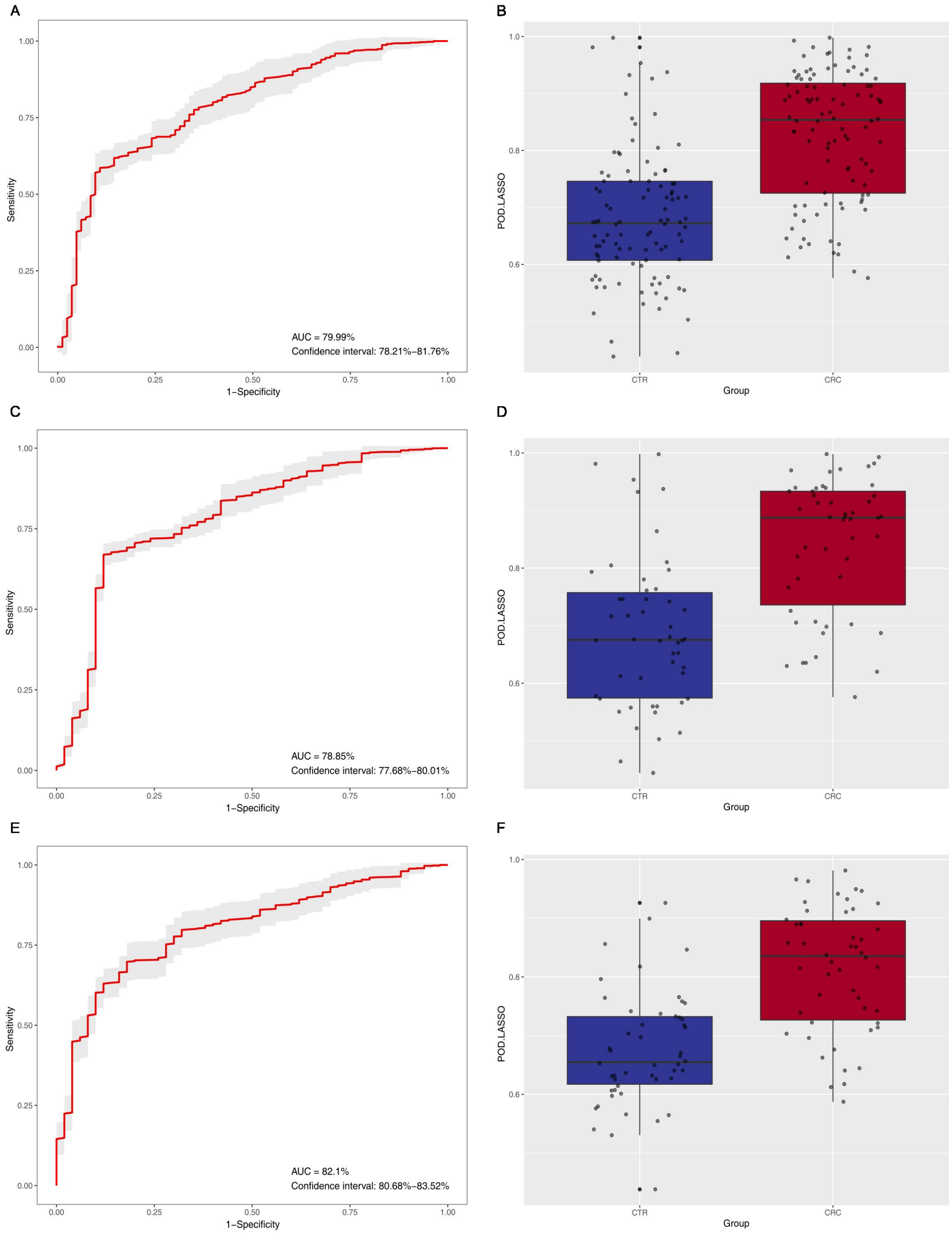

Supplementary Figure 10. Independent validation and diagnostic performance by LASSO classifier for CRC in external Fudan cohort. (A) The POD-based AUROC value between CRC and Control in the validation set, grey area denotes ± 1 standard deviation for AUROC value. (B) POD-score distribution across non-cancer individuals and cancer patients for the whole group (CTR, n=100; CRC, n=100) the independent validation phase. The box denotes 25th-75th percentiles, and the

central mark indicates the median; p value is calculated by two-sided unpaired Mann-Whitney test. (C) The POD-based AUROC value between CRC and Control for younger group, grey area denotes ±1 standard deviation for AUROC value. (D) POD-score distribution across non-cancer individuals and cancer patients for younger group (CTR, n=50; CRC, n=50). The box denotes 25th-75th percentiles, and the central mark indicates the median; p value is calculated by two-sided unpaired Mann-Whitney test. (E) The POD-based AUROC value between CRC and Control for elderly group, grey area denotes ±1 standard deviation for AUROC value. (F) POD-score distribution across non-cancer individuals and cancer patients for elderly group (CTR, n=50; CRC, n=50). The box denotes 25th-75th percentiles, and the central mark indicates the median; p value is calculated by two-sided unpaired Mann-Whitney test.

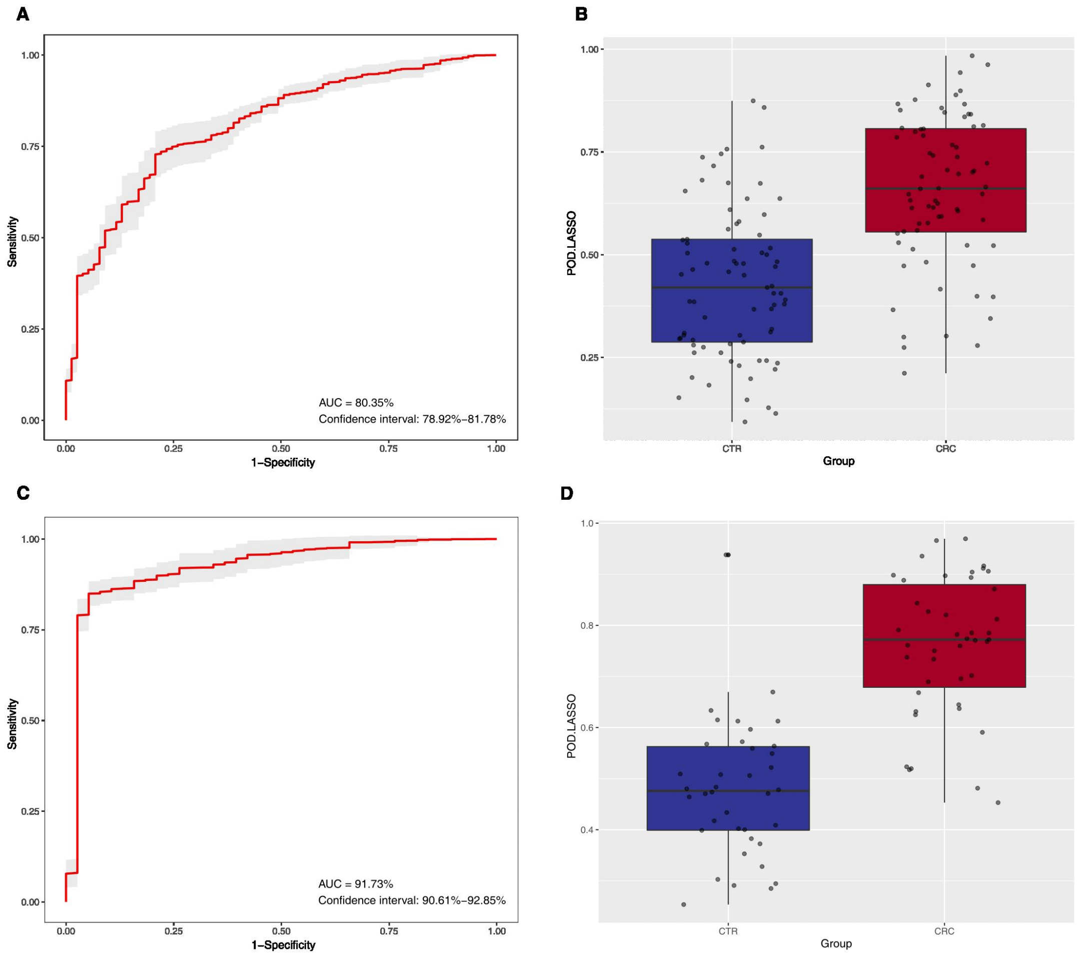

Supplementary Figure 11. Independent validation and diagnostic performance by LASSO classifier for CRC in external GloriousMed (A, B) and HK (C, D) cohorts. (A) The POD-based AUROC value between CRC and Control in the validation set, grey area denotes ± 1 standard deviation. (B) POD-score distribution across non-cancer individuals and cancer patients (CTR, n=76; CRC, n=82) in the independent validation phase. The box denotes 25th-75th percentiles, and the central mark indicates the median; p value is calculated by two-sided unpaired Mann-Whitney test. (C) The POD-based AUROC value between CRC and Control in the validation set, grey area denotes ± 1 standard deviation. (D) POD-score distribution across non-cancer individuals and cancer patients (CTR, n=38; CRC, n=43) in the independent validation phase. The box denotes 25th-75th percentiles, and the central mark indicates the median; p value is calculated by two-sided unpaired Mann-Whitney test.
