# Supplementary material for: Taxonomic and functional profiling of fecal metagenomes for the early detection of colorectal cancer
Source: Front Oncol. 2023 Aug 3;13:1218056. doi: 10.3389/fonc.2023.1218056 (PMC10436198; doi:10.3389/fonc.2023.1218056)
Supplement: Supplementary file 2 [file Table_1.pdf]

Supplementary Materials

Supplementary Tables

Supplementary Table S1. Clinical features of the study cohorts including age, gender, TNM stage, BMI, and cancer location.

Categories		Discovery		Validation	
		Healthy	CRC	Healthy	CRC
**Number**		52	121	44	67
**Age**		63	60	52	60
**Gender**	**Female**	27	77	26	33
	**Male**	25	44	18	34
**Overall Clinical Stage, n**	**Stage I**	0	8	0	6
	**Stage II**	0	10	0	10
	**Stage III**	0	27	0	17
	**Stage IV**	0	8	0	6
	**Missing/Not staged**	0	68	0	28
**Body Mass Index (BMI)**	**Underweight**	4	6	0	7
	**Normal**	33	61	11	37
	**Overweight**	13	42	15	13
	**Obese**	2	12	18	10
**Location**	**Colon**	0	53	0	29
	**Rectum**	0	59	0	37
	**Others**	0	9	0	1

Supplementary Table S2. Clinical data for the subjects in the discovery cohort.

Subject_ID	BMI	Group	Age	Gender	Tumor_location	Stage
F300011223_L01_13	24.2	CRC	60	M	rectum	I
F300011223_L01_14	19.9	CRC	42	F	colon	IV
F300011223_L01_81	22.3	CRC	72	M	rectum	NA
F300011223_L01_85	23.4	CRC	44	M	rectum	III
F300011223_L01_86	17.7	CRC	59	F	colon	III
F300011223_L01_89	20.4	CRC	60	F	other	IV
F300011223_L01_91	21.4	CRC	59	M	colon	NA
F300011223_L01_93	28.7	CRC	70	M	colon	NA
F300011223_L01_94	19.3	CRC	54	M	rectum	III
F300011223_L01_96	17.5	CRC	59	F	rectum	I
F300011227_L01_57	23.5	CRC	70	F	colon	II
F300011227_L01_60	24.5	CRC	50	M	rectum	III
F300011227_L01_61	26.4	CRC	76	M	Sigmoid take-off	IV

Subject_ID	BMI	Group	Age	Gender	Tumor_location	Stage
F300011227_L01_73	26.6	CRC	61	M	colon	III
F300011227_L01_74	22.5	CRC	50	M	rectum	III
F300011227_L01_76	28.2	CRC	47	M	colon	II
F300011227_L01_78	26.2	CRC	72	M	colon	I
F300011227_L01_79	18.2	CRC	64	F	colon	III
F300011239_L01_1	25.6	CRC	62	F	rectum	IV
F300011239_L01_2	20.8	CRC	53	F	colon	II
F300011239_L01_3	25.1	CRC	51	M	rectum	III
F300011239_L01_41	23.9	CRC	79	M	rectum	IV
F300011239_L01_43	19.6	CRC	66	F	rectum	NA
F300011239_L01_44	24.2	CRC	62	M	rectum	NA
F300011239_L01_45	17.4	CRC	67	F	colon	NA
F300011239_L01_46	20.0	CRC	51	F	colon	NA
F300011239_L01_48	21.1	CRC	75	F	rectum	III
F300011239_L01_58	24.4	CRC	61	M	colon	II
F300011239_L01_59	21.9	CRC	64	F	colon	NA
F300011239_L01_62	22.8	CRC	72	M	rectum	III
F300011246_L01_13	22.6	CRC	50	M	rectum	NA
F300011246_L01_14	24.1	CRC	66	M	colon	NA
F300011246_L01_16	25.1	CRC	63	M	rectum, colon	NA
F300011246_L01_65	22.3	CRC	49	M	rectum	NA
F300011246_L01_66	27.6	CRC	72	M	rectum	NA
F300011246_L01_67	25.8	CRC	62	M	rectum	NA
F300011246_L01_68	19.0	CRC	74	M	colon	NA
F300011246_L01_69	24.2	CRC	54	M	colon	NA
F300011246_L01_70	20.2	CRC	61	M	rectum	NA
F300011246_L01_73	22.1	CRC	52	F	colon	IV
F300011246_L01_76	18.8	CRC	57	F	colon	NA
F300011246_L01_78	24.7	CRC	62	F	rectum	III
F300011246_L01_79	18.6	CRC	70	M	colon	NA
F300011362_L01_66	28.3	CRC	65	F	colon	III
F300011362_L01_68	22.6	CRC	47	F	Sigmoid take-off	IV
F300011362_L01_69	23.3	CRC	52	M	rectum	III
F300011362_L01_72	24.9	CRC	67	M	rectum	I
F300011362_L02_1	20.3	Healthy	71	F	NA	NA
F300011362_L02_3	19.2	Healthy	79	F	NA	NA
F300011362_L02_80	24.7	Healthy	67	F	NA	NA
F300011368_L01_100	26.6	CRC	69	M	rectum, colon	III
F300011368_L01_101	20.4	CRC	60	F	rectum	II
F300011368_L01_104	21.8	CRC	53	M	colon	I
F300011368_L01_13	19.6	CRC	62	M	rectum	III
F300011368_L01_14	27.0	CRC	53	F	colon	II
F300011368_L01_15	22.0	CRC	43	M	rectum	III

Subject_ID	BMI	Group	Age	Gender	Tumor_location	Stage
F300011368_L01_91	24.8	CRC	76	M	colon	I
F300011368_L01_92	22.7	CRC	58	M	rectum	NA
F300011368_L01_93	26.1	CRC	59	M	colon	II
F300011368_L01_94	23.3	CRC	65	M	rectum	III
F300011368_L01_95	25.3	CRC	61	M	rectum	III
F300011368_L01_97	25.2	CRC	75	M	colon	III
F300011368_L01_99	23.7	CRC	63	M	rectum	III
F300011370_L01_2	25.0	CRC	66	M	colon	II
F300011370_L01_3	30.8	CRC	48	M	rectum	III
F300011370_L01_4	24.3	CRC	66	F	colon	II
F300011370_L01_46	21.1	CRC	52	M	rectum	III
F300011370_L01_81	25.6	CRC	52	F	rectum	III
F300011370_L01_82	27.9	CRC	64	F	colon	III
F300011370_L01_83	25.1	CRC	52	M	rectum	III
F300011370_L01_86	21.7	CRC	53	F	colon	I
F300011370_L01_87	21.5	CRC	59	M	rectum	IV
F300011370_L01_88	19.5	CRC	75	F	colon	III
F300011379_L01_42	24.1	CRC	68	M	rectum	III
F300011379_L02_57	21.5	Healthy	62	F	NA	NA
F300011379_L02_58	22.6	Healthy	70	M	NA	NA
F300011379_L02_59	29.6	Healthy	67	F	NA	NA
F300011379_L02_60	25.9	Healthy	75	M	NA	NA
F300011379_L02_61	20.4	Healthy	66	F	NA	NA
F300011379_L02_62	20.8	Healthy	70	F	NA	NA
F300011384_L01_81	16.7	Healthy	49	F	NA	NA
F300011384_L01_82	22.0	Healthy	45	M	NA	NA
F300011384_L01_84	20.0	Healthy	57	F	NA	NA
F300011384_L01_87	24.6	Healthy	68	M	NA	NA
F300011384_L01_91	17.3	Healthy	62	F	NA	NA
F300011384_L01_93	20.0	Healthy	57	F	NA	NA
F300011384_L01_94	23.4	Healthy	53	M	NA	NA
V350088889_L04_114	23.0	CRC	58	M	rectum	NA
V350088889_L04_115	37.9	CRC	68	F	colon	NA
V350088889_L04_116	22.8	CRC	72	F	colon	NA
V350088889_L04_117	18.7	CRC	62	M	rectum	II
V350088889_L04_13	27.8	CRC	67	F	colon	NA
V350088889_L04_14	21.8	CRC	71	M	rectum	NA
V350088889_L04_15	20.9	CRC	73	M	rectum	NA
V350088889_L04_16	16.8	CRC	59	M	rectum	NA
V350088889_L04_26	25.3	CRC	55	F	colon	I
V350088889_L04_29	23.2	CRC	68	M	colon	NA
V350088889_L04_30	24.5	CRC	55	F	colon	NA
V350088889_L04_32	24.9	CRC	66	M	colon	NA

Subject_ID	BMI	Group	Age	Gender	Tumor_location	Stage
V350088889_L04_33	26.4	CRC	46	M	colon	NA
V350088889_L04_34	25.6	CRC	68	M	rectum	NA
V350088889_L04_35	22.6	CRC	60	F	rectum	NA
V350088889_L04_38	18.7	CRC	61	F	other	NA
V350088889_L04_39	22.3	CRC	73	M	rectum	NA
V350088889_L04_41	21.3	CRC	61	M	colon	NA
V350088889_L04_42	21.7	CRC	65	M	rectum	NA
V350088889_L04_43	22.5	CRC	48	F	rectum	NA
V350088889_L04_44	28.6	CRC	60	M	rectum	NA
V350088889_L04_45	24.2	CRC	49	M	rectum	NA
V350088889_L04_46	21.9	CRC	71	M	colon	NA
V350088889_L04_48	29.4	CRC	55	M	colon	NA
V350088889_L04_49	19.8	CRC	45	F	other	NA
V350088889_L04_50	19.8	CRC	56	M	rectum	NA
V350088889_L04_51	25.1	CRC	62	M	rectum	NA
V350088889_L04_53	24.0	CRC	52	F	colon	NA
V350088889_L04_55	25.3	CRC	47	M	other	NA
V350088889_L04_56	24.2	CRC	65	M	rectum	NA
V350088889_L04_57	28.1	CRC	56	M	rectum	NA
V350088889_L04_58	24.1	CRC	56	F	colon	NA
V350088889_L04_59	25.2	CRC	59	M	rectum	NA
V350088889_L04_60	20.4	CRC	60	M	rectum	NA
V350088889_L04_61	26.1	CRC	57	M	colon	NA
V350088889_L04_62	20.7	CRC	66	M	rectum	NA
V350088889_L04_63	23.7	CRC	56	F	colon	NA
V350088889_L04_64	19.5	CRC	75	F	colon	NA
V350088889_L04_65	22.8	CRC	62	M	Sigmoid take-off	NA
V350088889_L04_66	18.7	CRC	55	M	rectum	NA
V350088889_L04_67	19.5	CRC	65	F	colon	NA
V350088889_L04_68	21.5	CRC	56	M	colon	NA
V350088889_L04_69	21.0	CRC	61	M	colon	NA
V350088889_L04_70	23.5	CRC	46	F	colon	NA
V350088889_L04_71	24.3	CRC	61	M	colon	NA
V350088889_L04_72	18.2	CRC	62	F	rectum	NA
V350088889_L04_73	21.5	CRC	48	F	rectum	NA
V350088889_L04_76	25.0	CRC	60	F	rectum	NA
V350088889_L04_78	26.0	CRC	67	F	rectum	NA
V350088889_L04_79	26.3	CRC	57	M	colon	NA
V350089121_L04_100	22.0	Healthy	48	M	NA	NA
V350089121_L04_103	20.4	Healthy	51	M	NA	NA
V350089121_L04_104	21.8	Healthy	67	F	NA	NA
V350089121_L04_114	18.9	Healthy	67	F	NA	NA
V350089121_L04_121	21.1	Healthy	68	F	NA	NA

Subject_ID	BMI	Group	Age	Gender	Tumor_location	Stage
V350089121_L04_123	28.7	Healthy	64	F	NA	NA
V350089121_L04_124	14.5	Healthy	75	F	NA	NA
V350089121_L04_125	19.7	Healthy	73	F	NA	NA
V350089121_L04_126	23.4	Healthy	75	M	NA	NA
V350089121_L04_15	24.8	Healthy	67	F	NA	NA
V350089121_L04_29	24.8	Healthy	57	M	NA	NA
V350089121_L04_30	22.0	Healthy	50	F	NA	NA
V350089121_L04_33	25.0	Healthy	44	M	NA	NA
V350089121_L04_35	19.5	Healthy	65	M	NA	NA
V350089121_L04_37	20.2	Healthy	66	M	NA	NA
V350089121_L04_38	22.0	Healthy	58	M	NA	NA
V350089121_L04_51	20.8	Healthy	45	M	NA	NA
V350089121_L04_52	20.3	Healthy	52	F	NA	NA
V350089121_L04_53	20.3	Healthy	48	M	NA	NA
V350089121_L04_81	21.3	Healthy	67	M	NA	NA
V350089121_L04_82	21.3	Healthy	56	F	NA	NA
V350089121_L04_83	22.2	Healthy	71	F	NA	NA
V350089121_L04_84	23.1	Healthy	67	M	NA	NA
V350089121_L04_85	26.4	Healthy	69	M	NA	NA
V350089121_L04_86	24.7	Healthy	69	F	NA	NA
V350089121_L04_87	21.9	Healthy	65	M	NA	NA
V350089121_L04_88	22.0	Healthy	70	M	NA	NA
V350089121_L04_89	26.3	Healthy	70	F	NA	NA
V350089121_L04_90	19.5	Healthy	63	F	NA	NA
V350089121_L04_91	22.5	Healthy	70	M	NA	NA
V350089121_L04_92	24.9	Healthy	59	M	NA	NA
V350089121_L04_95	26.8	Healthy	73	F	NA	NA
V350089121_L04_96	25.4	Healthy	67	M	NA	NA
V350089121_L04_97	17.6	Healthy	57	F	NA	NA
V350089121_L04_98	26.3	Healthy	66	M	NA	NA
V350089121_L04_99	21.3	Healthy	71	M	NA	NA

Supplementary Table S3. Clinical data for the subjects in the validation cohort.

Subject_ID	BMI	Group	Age	Gender	Tumor_location	Stage
F300011223_L01_15	28.4	CRC	45	M	colon	II
F300011223_L01_16	24.6	CRC	57	M	colon	II
F300011223_L01_82	21.6	CRC	65	M	rectum	NA
F300011223_L01_83	25.5	CRC	69	F	colon	NA
F300011223_L01_84	20.9	CRC	51	F	rectum	I
F300011223_L01_87	19.0	CRC	47	F	rectum	III

Subject_ID	BMI	Group	Age	Gender	Tumor_location	Stage
F300011223_L01_88	22.2	CRC	69	F	rectum	IV
F300011223_L01_90	19.3	CRC	73	M	colon	II
F300011223_L01_92	20.3	CRC	74	F	colon	I
F300011223_L01_95	20.1	CRC	64	M	colon	II
F300011227_L01_58	28.0	CRC	75	F	rectum	NA
F300011227_L01_59	23.1	CRC	76	F	rectum	NA
F300011227_L01_75	24.7	CRC	50	M	colon	IV
F300011227_L01_77	20.2	CRC	66	M	colon	III
F300011227_L01_80	20.8	CRC	51	M	colon	IV
F300011239_L01_4	20.6	CRC	71	M	colon	III
F300011239_L01_42	22.4	CRC	65	M	colon	I
F300011239_L01_47	19.6	CRC	64	M	colon	NA
F300011239_L01_57	22.2	CRC	57	F	rectum	III
F300011239_L01_60	29.4	CRC	62	F	colon	NA
F300011239_L01_61	23.2	CRC	67	F	rectum	I
F300011239_L01_63	21.9	CRC	63	F	rectum	III
F300011239_L01_64	25.8	CRC	62	M	rectum	NA
F300011246_L01_15	28.1	CRC	70	M	rectum	NA
F300011246_L01_71	18.2	CRC	71	F	rectum	NA
F300011246_L01_72	20.8	CRC	65	M	rectum	III
F300011246_L01_74	23.9	CRC	69	F	rectum	II
F300011246_L01_75	24.8	CRC	62	F	rectum	NA
F300011246_L01_77	20.0	CRC	45	F	rectum	III
F300011246_L01_80	23.4	CRC	61	M	colon	IV
F300011362_L01_1	17.1	CRC	51	M	colon	II
F300011362_L01_3	25.2	CRC	68	M	colon	IV
F300011362_L01_65	19.3	CRC	41	M	rectum	I
F300011362_L01_67	21.3	CRC	68	F	rectum	II
F300011362_L01_70	21.0	CRC	61	F	other	III
F300011362_L01_71	27.8	CRC	47	M	colon	II
F300011362_L02_73	28.8	Healthy	54	F	NA	NA
F300011362_L02_74	24.8	Healthy	49	F	NA	NA
F300011362_L02_75	26.9	Healthy	47	M	NA	NA
F300011362_L02_76	28.7	Healthy	48	F	NA	NA
F300011362_L02_77	24.8	Healthy	43	F	NA	NA
F300011362_L02_78	28.0	Healthy	67	M	NA	NA
F300011362_L02_79	21.9	Healthy	45	F	NA	NA
F300011368_L01_102	21.5	CRC	60	M	rectum	IV
F300011368_L01_103	20.8	CRC	59	F	colon	II
F300011368_L01_16	27.2	CRC	57	M	rectum	III
F300011368_L01_89	18.0	CRC	78	M	rectum	I
F300011368_L01_90	18.2	CRC	66	F	colon	NA
F300011368_L01_96	27.4	CRC	47	M	rectum	NA

Subject_ID	BMI	Group	Age	Gender	Tumor_location	Stage
F300011368_L01_98	23.7	CRC	62	F	colon	III
F300011370_L01_1	23.6	CRC	55	F	colon	NA
F300011370_L01_41	18.6	CRC	70	F	colon	NA
F300011370_L01_42	25.2	CRC	53	M	colon	NA
F300011370_L01_43	19.1	CRC	71	F	colon	NA
F300011370_L01_44	26.4	CRC	51	M	colon	NA
F300011370_L01_45	19.2	CRC	62	F	colon	NA
F300011370_L01_47	23.5	CRC	51	F	rectum	III
F300011370_L01_48	21.0	CRC	61	M	rectum	III
F300011370_L01_84	27.3	CRC	76	M	colon	NA
F300011370_L01_85	23.9	CRC	67	M	rectum	II
F300011379_L01_2	17.9	CRC	58	F	rectum	NA
F300011379_L01_4	25.3	Healthy	49	F	NA	NA
F300011379_L01_41	27.3	CRC	51	M	rectum	III
F300011379_L01_44	24.5	CRC	66	F	colon	III
F300011379_L01_46	19.6	CRC	52	M	rectum	III
F300011379_L02_63	22.2	Healthy	70	F	NA	NA
F300011379_L02_64	25.9	Healthy	60	M	NA	NA
F300011384_L01_83	28.6	Healthy	45	M	NA	NA
F300011384_L01_85	24.9	Healthy	56	F	NA	NA
F300011384_L01_86	25.9	Healthy	48	F	NA	NA
F300011384_L01_88	29.3	Healthy	45	M	NA	NA
F300011384_L01_89	24.5	Healthy	56	F	NA	NA
F300011384_L01_90	28.0	Healthy	42	M	NA	NA
F300011384_L01_92	26.7	Healthy	60	M	NA	NA
F300011384_L01_95	27.3	Healthy	47	M	NA	NA
V350088889_L04_25	17.3	CRC	65	M	rectum	III
V350088889_L04_28	18.9	CRC	62	F	rectum	NA
V350088889_L04_36	20.1	CRC	64	F	colon	NA
V350088889_L04_37	21.6	CRC	57	F	rectum	NA
V350088889_L04_47	27.6	CRC	63	F	rectum	NA
V350088889_L04_52	25.0	CRC	52	M	rectum	NA
V350088889_L04_74	24.3	CRC	54	M	rectum	NA
V350088889_L04_75	19.2	CRC	51	F	rectum	NA
V350088889_L04_77	24.0	CRC	52	F	rectum	NA
V350088889_L04_80	17.0	CRC	57	M	rectum	III
V350089121_L04_1	20.6	Healthy	49	F	NA	NA
V350089121_L04_101	26.4	Healthy	47	F	NA	NA
V350089121_L04_102	27.4	Healthy	52	F	NA	NA
V350089121_L04_115	24.0	Healthy	52	F	NA	NA
V350089121_L04_116	23.1	Healthy	42	F	NA	NA
V350089121_L04_117	21.1	Healthy	63	F	NA	NA
V350089121_L04_122	29.4	Healthy	62	F	NA	NA

Subject_ID	BMI	Group	Age	Gender	Tumor_location	Stage
V350089121_L04_127	27.6	Healthy	56	F	NA	NA
V350089121_L04_128	30.4	Healthy	54	M	NA	NA
V350089121_L04_13	19.5	Healthy	49	F	NA	NA
V350089121_L04_2	26.4	Healthy	49	F	NA	NA
V350089121_L04_25	20.0	Healthy	52	F	NA	NA
V350089121_L04_26	27.4	Healthy	54	M	NA	NA
V350089121_L04_28	22.3	Healthy	50	F	NA	NA
V350089121_L04_3	27.6	Healthy	68	M	NA	NA
V350089121_L04_32	26.8	Healthy	52	M	NA	NA
V350089121_L04_34	22.3	Healthy	58	F	NA	NA
V350089121_L04_36	29.7	Healthy	45	M	NA	NA
V350089121_L04_39	28.2	Healthy	40	M	NA	NA
V350089121_L04_4	29.4	Healthy	51	M	NA	NA
V350089121_L04_49	26.0	Healthy	53	F	NA	NA
V350089121_L04_50	29.0	Healthy	50	M	NA	NA
V350089121_L04_55	22.8	Healthy	46	F	NA	NA
V350089121_L04_56	24.9	Healthy	63	M	NA	NA
V350089121_L04_93	18.9	Healthy	66	F	NA	NA
V350089121_L04_94	29.3	Healthy	56	M	NA	NA

Supplementary Table S4. Sequencing reads information aligned to GTDB and IGC for the subjects enrolled in the two cohorts (284 metagenomes).

Subject_ID	Total Reads	Clean Reads	Unique Mapped Reads (GTDB Species)	Mapped Gene (IGC)
F300011223_L01_13	31906094	29732462	15564323	622436
F300011223_L01_14	31103820	11434654	5619421	390213
F300011223_L01_15	39603376	37639492	19798394	628979
F300011223_L01_16	29466765	28157233	12204330	557138
F300011223_L01_81	26771894	24142738	10842834	595425
F300011223_L01_82	29176450	26630612	12010813	340827
F300011223_L01_83	30150233	26770835	14618903	758035
F300011223_L01_84	28222585	25398811	10145616	246555
F300011223_L01_85	28767600	27609719	14413271	803680
F300011223_L01_86	28219570	25049255	8119888	334715
F300011223_L01_87	25995428	22153603	11274435	335939
F300011223_L01_88	28986280	27253751	12093493	432241
F300011223_L01_89	30032822	27996928	14378034	199438
F300011223_L01_90	25713445	23298403	10098624	702307
F300011223_L01_91	30410916	28147193	17531267	456920
F300011223_L01_92	29761605	27140983	14223960	564962

Subject_ID	Total Reads	Clean Reads	Unique Mapped Reads (GTDB Species)	Mapped Gene (IGC)
F300011223_L01_93	28889505	27276319	9593231	624224
F300011223_L01_94	28320425	26647345	13589074	633459
F300011223_L01_95	23802054	22096505	10938972	626831
F300011223_L01_96	31511646	29129839	10239646	284383
F300011227_L01_57	27825750	26513792	15630921	730125
F300011227_L01_58	28896549	27119947	15114889	718093
F300011227_L01_59	30611072	24866721	11745385	589417
F300011227_L01_60	26066414	24037328	8449443	541272
F300011227_L01_61	22745751	20680286	10255965	573272
F300011227_L01_73	32087661	30012632	14713976	807518
F300011227_L01_74	30121619	28130015	14925462	847139
F300011227_L01_75	27004660	25272337	14496719	471918
F300011227_L01_76	26307040	24011088	9764901	346808
F300011227_L01_77	28203082	24951076	11508062	324834
F300011227_L01_78	26013942	24671818	13557440	738560
F300011227_L01_79	30141488	28120424	11427095	390837
F300011227_L01_80	26432679	25084653	10695334	555180
F300011239_L01_1	28613168	24116923	6857823	303078
F300011239_L01_2	27697479	24384936	10268230	416116
F300011239_L01_3	35597835	33655370	16680018	833134
F300011239_L01_4	28335874	23539344	9115646	584346
F300011239_L01_41	16460179	14132698	6987473	719815
F300011239_L01_42	29092897	27485117	13755858	693005
F300011239_L01_43	30830977	28020283	15083740	326320
F300011239_L01_44	31145579	29271263	12783732	668427
F300011239_L01_45	33748184	32206253	13076115	463062
F300011239_L01_46	29655270	27631249	13067080	326418
F300011239_L01_47	29446868	22040146	12921440	542054
F300011239_L01_48	32310437	25637502	12916089	661199
F300011239_L01_57	29077013	18339441	9056343	545957
F300011239_L01_58	31790157	30422716	17950004	798635
F300011239_L01_59	31515198	30121735	11717460	429707
F300011239_L01_60	30425414	27731646	9800377	293841
F300011239_L01_61	15292375	14176109	7746248	620392
F300011239_L01_62	29510668	23493654	13220776	921709
F300011239_L01_63	33566740	30883315	17121604	517562
F300011239_L01_64	32978928	30997475	13987876	553110
F300011246_L01_13	28941635	26906648	14701671	801802
F300011246_L01_14	28937071	26689568	14877629	272649
F300011246_L01_15	28056723	22772690	8812409	538402
F300011246_L01_16	28651391	27154111	14325327	727639
F300011246_L01_65	27098159	22905578	10278765	456283
F300011246_L01_66	26998868	25108241	8991762	568415

Subject_ID	Total Reads	Clean Reads	Unique Mapped Reads (GTDB Species)	Mapped Gene (IGC)
F300011246_L01_67	27866785	25619007	13271191	929780
F300011246_L01_68	29234672	23047868	8227889	359164
F300011246_L01_69	26460071	23129994	9730539	671693
F300011246_L01_70	24662852	23378037	9735931	550692
F300011246_L01_71	26953959	24367040	11384507	235987
F300011246_L01_72	28680058	10313036	5594490	429650
F300011246_L01_73	31575877	29576252	12300551	580366
F300011246_L01_74	33228416	29386850	16784790	686214
F300011246_L01_75	23993150	22072205	12202655	511311
F300011246_L01_76	27754444	26075354	11273466	591863
F300011246_L01_77	27683039	21890465	9123495	359559
F300011246_L01_78	28722940	27026577	12841967	339353
F300011246_L01_79	29788271	28427276	14862013	372495
F300011246_L01_80	28597816	26368316	12678093	424837
F300011362_L01_1	14772789	13463482	4615538	305409
F300011362_L01_3	19171678	18500772	10016480	659815
F300011362_L01_65	35557259	32941472	13812534	366914
F300011362_L01_66	32938851	31069656	9295175	385587
F300011362_L01_67	33554816	30422719	18396550	508735
F300011362_L01_68	29292453	25919602	9355282	523345
F300011362_L01_69	34374713	32399138	11889948	643922
F300011362_L01_70	30803936	28011035	15568240	240707
F300011362_L01_71	32954472	30551334	11494075	340811
F300011362_L01_72	28592863	25292731	9878071	164220
F300011362_L02_1	16650820	14812172	11104206	489282
F300011362_L02_3	17488120	16440092	11219580	349633
F300011362_L02_73	31765407	30266886	11869494	395041
F300011362_L02_74	31320378	30192518	14684465	496022
F300011362_L02_75	31780269	30209384	12548699	475528
F300011362_L02_76	33220776	31827924	13017767	515258
F300011362_L02_77	33542057	32431499	14194055	522902
F300011362_L02_78	35776213	34020194	16773578	681890
F300011362_L02_79	35785662	34538241	19928579	521092
F300011362_L02_80	31495230	28924219	12100313	418406
F300011368_L01_100	16092597	15052635	6462756	522666
F300011368_L01_101	15034587	14122330	5098110	300438
F300011368_L01_102	14906342	14245746	6683424	267617
F300011368_L01_103	14306821	12763832	6071514	434174
F300011368_L01_104	16588777	15791792	7304399	671963
F300011368_L01_13	15314557	13893949	4763835	176348
F300011368_L01_14	15977578	14892367	6643547	687533
F300011368_L01_15	15498763	14363332	6594990	536759
F300011368_L01_16	14342504	13485003	4656538	389189

Subject_ID	Total Reads	Clean Reads	Unique Mapped Reads (GTDB Species)	Mapped Gene (IGC)
F300011368_L01_89	14930404	13953015	4632721	181468
F300011368_L01_90	15026285	14260665	7362658	155845
F300011368_L01_91	16082478	13789110	5278448	479399
F300011368_L01_92	14714678	13940243	6357513	713838
F300011368_L01_93	15772987	14878429	7681153	874219
F300011368_L01_94	14880751	12794233	7304378	652959
F300011368_L01_95	15566243	14803661	7252728	478401
F300011368_L01_96	16198347	14680810	7501734	434654
F300011368_L01_97	15509424	14766270	7147536	465515
F300011368_L01_98	14813451	13447109	4298666	284639
F300011368_L01_99	14984926	13045463	5098191	558175
F300011370_L01_1	16138282	11970923	6315592	386873
F300011370_L01_2	17707230	14468975	5869023	456008
F300011370_L01_3	14982868	13557455	7306629	486767
F300011370_L01_4	15547331	14560514	8289006	718487
F300011370_L01_41	15364935	14455507	8070170	221290
F300011370_L01_42	16330309	15414970	5521450	444952
F300011370_L01_43	13746907	12846413	5706846	423764
F300011370_L01_44	16774259	16083482	8442956	746368
F300011370_L01_45	16297197	15585012	7019443	224090
F300011370_L01_46	17301281	16382317	8398933	924813
F300011370_L01_47	17970156	16821347	8443506	724075
F300011370_L01_48	17667537	16955612	9044894	1048765
F300011370_L01_81	14668832	13716222	6214863	579650
F300011370_L01_82	15852576	15038564	4577606	340737
F300011370_L01_83	15480238	14412749	7181794	371322
F300011370_L01_84	18204780	16886629	8392942	640338
F300011370_L01_85	15519321	13039451	5857132	291446
F300011370_L01_86	16005126	15282505	6244847	543591
F300011370_L01_87	15074086	13781696	7361667	508910
F300011370_L01_88	15858089	15091650	5873121	603575
F300011379_L01_2	24735199	22944963	10051139	338234
F300011379_L01_4	23549983	22230221	9888200	349559
F300011379_L01_41	23480722	22054376	10214311	598478
F300011379_L01_42	26639120	24934627	13388999	518433
F300011379_L01_44	24524624	23022915	12320442	501539
F300011379_L01_46	23474046	22371158	12351000	926946
F300011379_L02_57	40368086	37285458	18378278	583453
F300011379_L02_58	38876468	37445716	21155486	609940
F300011379_L02_59	38998763	33597513	14167237	339832
F300011379_L02_60	38151939	36328240	19441750	807974
F300011379_L02_61	35346862	31469224	14535175	652741
F300011379_L02_62	36199455	34863609	13758954	326512

Subject_ID	Total Reads	Clean Reads	Unique Mapped Reads (GTDB Species)	Mapped Gene (IGC)
F300011379_L02_63	41877390	39829216	17356070	773649
F300011379_L02_64	32518596	30660316	15179938	467981
F300011384_L01_81	22091907	20907893	8626991	318011
F300011384_L01_82	28497461	27102580	14895555	715482
F300011384_L01_83	38473670	36387657	16744993	498193
F300011384_L01_84	20466899	19060950	6669305	350944
F300011384_L01_85	22396227	20830236	9077259	387063
F300011384_L01_86	21849940	20722820	7117859	553309
F300011384_L01_87	23402482	22114826	9506537	673425
F300011384_L01_88	22770050	21104870	13653546	241689
F300011384_L01_89	28175063	26993771	12548912	609420
F300011384_L01_90	26153182	24584197	11978341	582701
F300011384_L01_91	25415555	23531882	11071750	375900
F300011384_L01_92	31713063	30138357	9258942	434271
F300011384_L01_93	28198389	26682544	9855408	563443
F300011384_L01_94	29828690	28593422	14662629	703813
F300011384_L01_95	25025542	23613748	9680071	519161
V350088889_L04_114	31303830	28315833	14234847	287816
V350088889_L04_115	31025784	28363619	11900869	527453
V350088889_L04_116	30324727	25905046	14359534	628238
V350088889_L04_117	35585261	31160190	18611196	694618
V350088889_L04_13	31865896	29911401	14826755	179374
V350088889_L04_14	30470514	29007347	16023239	803460
V350088889_L04_15	29728572	27190136	12554721	494942
V350088889_L04_16	28795980	25189312	12442395	788762
V350088889_L04_25	27749566	24915023	10427406	668187
V350088889_L04_26	30727521	25992501	14172288	526373
V350088889_L04_28	35284040	31864001	15507712	525244
V350088889_L04_29	28421517	25461347	11172422	509720
V350088889_L04_30	27649660	26145268	13512945	932999
V350088889_L04_32	27619178	25843199	10604512	268546
V350088889_L04_33	31842681	22486029	8011592	365056
V350088889_L04_34	27378516	25669941	12298045	431596
V350088889_L04_35	36043036	31989811	19185170	728381
V350088889_L04_36	31092662	29727762	11840975	341666
V350088889_L04_37	30725636	28059094	10755978	393637
V350088889_L04_38	29750210	27722305	10211042	364106
V350088889_L04_39	33586265	31906905	15889822	749829
V350088889_L04_41	26727476	23832114	10956592	464561
V350088889_L04_42	29731311	26022459	9801536	180367
V350088889_L04_43	28619004	27204186	8216474	237461
V350088889_L04_44	29668127	26965475	15689889	338979
V350088889_L04_45	31153932	29051425	14348380	319007

Subject_ID	Total Reads	Clean Reads	Unique Mapped Reads (GTDB Species)	Mapped Gene (IGC)
V350088889_L04_46	33412087	29479384	10471379	386149
V350088889_L04_47	30390246	28498794	11063516	331745
V350088889_L04_48	32195782	28460225	11614961	334589
V350088889_L04_49	27774295	26078227	10969351	732899
V350088889_L04_50	31506189	27019336	11748944	489372
V350088889_L04_51	29368124	26415713	11758443	487810
V350088889_L04_52	32376464	30536062	18591652	615334
V350088889_L04_53	35883440	24236952	11273373	150271
V350088889_L04_55	33697924	28663611	13001065	242372
V350088889_L04_56	32994457	31106539	15510107	485090
V350088889_L04_57	26045485	24305369	11532649	605102
V350088889_L04_58	31636680	28394863	15712362	528989
V350088889_L04_59	28822346	27214257	11456527	530830
V350088889_L04_60	33138365	30590687	14063503	513642
V350088889_L04_61	27440263	25083982	11180751	366473
V350088889_L04_62	32254859	30608672	14286875	715362
V350088889_L04_63	29452739	27281973	12712929	327281
V350088889_L04_64	30985189	29561684	15538555	656416
V350088889_L04_65	27730107	24940479	13146659	430809
V350088889_L04_66	32108925	29503030	12409888	640130
V350088889_L04_67	34364099	32665750	15876123	406592
V350088889_L04_68	28080093	24397479	9124671	522800
V350088889_L04_69	30012833	27924051	13501949	478937
V350088889_L04_70	29585700	21854000	11525541	643722
V350088889_L04_71	38633862	34324792	15873289	566914
V350088889_L04_72	33075509	31603699	13640965	463899
V350088889_L04_73	33270398	31043400	15654138	858877
V350088889_L04_74	35727380	28288251	13629812	698924
V350088889_L04_75	30591219	28201638	11683417	304137
V350088889_L04_76	30232360	28415687	10952676	236118
V350088889_L04_77	28723852	25964548	12594578	490160
V350088889_L04_78	29714429	25796319	9300188	404764
V350088889_L04_79	28464869	23522828	8573460	280278
V350088889_L04_80	27136141	24719351	12655812	490139
V350089121_L04_1	30063744	28391435	11340920	503783
V350089121_L04_100	26332164	25170444	12460373	381817
V350089121_L04_101	29375002	27817047	9488560	389246
V350089121_L04_102	36657120	34567222	17456057	756083
V350089121_L04_103	30462027	28779362	12481536	417511
V350089121_L04_104	31321854	29650349	15302087	712036
V350089121_L04_114	36742973	35253595	19760630	680408
V350089121_L04_115	28869544	27380543	15462842	384820
V350089121_L04_116	27551427	26407551	9537360	373081

Subject_ID	Total Reads	Clean Reads	Unique Mapped Reads (GTDB Species)	Mapped Gene (IGC)
V350089121_L04_117	36704532	35760776	15646886	333858
V350089121_L04_121	29166320	27648043	9895048	352159
V350089121_L04_122	33688524	31871401	12057436	192373
V350089121_L04_123	30333434	29033911	11218262	613634
V350089121_L04_124	27604078	23091787	11343484	444583
V350089121_L04_125	31370211	29497846	9995882	176294
V350089121_L04_126	25973204	21621221	8236850	493460
V350089121_L04_127	29601199	28073707	14103542	705422
V350089121_L04_128	26117008	24164486	8741625	247791
V350089121_L04_13	19131362	15523780	10258048	338619
V350089121_L04_15	19127623	18079216	13545416	530374
V350089121_L04_2	38635622	34032852	11630757	308080
V350089121_L04_25	26905075	25851912	8368757	305333
V350089121_L04_26	28134456	26697782	10631076	430421
V350089121_L04_28	28324838	26425853	11470915	503935
V350089121_L04_29	30191585	27476646	11400029	439705
V350089121_L04_3	36189028	34362621	17504317	465983
V350089121_L04_30	29351252	25252827	11039746	388871
V350089121_L04_32	31192782	30003199	12910693	388949
V350089121_L04_33	29729911	27843699	9733085	226898
V350089121_L04_34	25068742	23823507	11139118	487591
V350089121_L04_35	28821565	27509825	12554922	683535
V350089121_L04_36	38808642	37739617	26138994	176773
V350089121_L04_37	30677420	27918563	9146455	157385
V350089121_L04_38	30845787	28595122	15843907	133507
V350089121_L04_39	31157285	29218186	9958374	275400
V350089121_L04_4	34811460	31212146	9707923	166212
V350089121_L04_49	30069384	28821014	14539327	250372
V350089121_L04_50	26243625	24126293	8021798	261215
V350089121_L04_51	28884251	27646260	10597074	438970
V350089121_L04_52	29528608	28399577	10682477	461873
V350089121_L04_53	31662145	30053756	13184575	602406
V350089121_L04_55	34024643	32455154	15127350	608867
V350089121_L04_56	27890409	25978269	12511498	628123
V350089121_L04_81	38312040	36608411	17822099	568125
V350089121_L04_82	28640777	27293001	5767549	567724
V350089121_L04_83	35107278	33592426	16231408	534299
V350089121_L04_84	32736388	30066431	12294112	286151
V350089121_L04_85	33172923	31872532	14912723	462854
V350089121_L04_86	35631666	33895742	16706450	798442
V350089121_L04_87	33024552	31751797	11776315	454199
V350089121_L04_88	28976161	27499828	13416922	714259
V350089121_L04_89	32404931	31056315	9539933	227113

Subject_ID	Total Reads	Clean Reads	Unique Mapped Reads (GTDB Species)	Mapped Gene (IGC)
V350089121_L04_90	35087934	32798745	13731969	282930
V350089121_L04_91	58496179	55728734	16526456	397779
V350089121_L04_92	33248307	31818072	16950235	732348
V350089121_L04_93	32602692	31131193	11202837	406270
V350089121_L04_94	32013634	30865314	15401846	556462
V350089121_L04_95	33979309	32181823	15340570	775686
V350089121_L04_96	34334869	32735817	14921701	613553
V350089121_L04_97	35393387	34369257	20856254	277160
V350089121_L04_98	35093167	33235178	18436724	734423
V350089121_L04_99	34317331	32383958	14322450	435225

Supplementary Table S5. The microbial species enriched or depleted in CRC and healthy control samples that have passed the deconfounding test.

Custom ID	GTDB + Genome Assembly Species	Deconfounding status	Ds	p value	q value
sp1953955	Prevotella_intermedia.GCF_001953955_1	OK_nc	0.76	2.92E-22	1.52E-25
sp147675	Peptostreptococcus_stomatis.GCF_000147675_1	OK_nc	0.74	3.62E-21	3.77E-24
sp55555000181695	Malassezia_globosa_CBS_7966.GCF_000181695_1	OK_nc	0.72	1.71E-20	2.67E-23
sp235465	Fusobacterium_sp000235465.GCF_000235465_1	OK_nc	0.64	5.21E-16	1.08E-18
sp758885	Peptostreptococcus_sp000758885.GCF_000758885_1	OK_nc	0.63	1.74E-15	4.54E-18
sp900637905	Parvimonas_micra.GCF_900637905_1	OK_nc	0.62	5.41E-15	1.69E-17
sp1457555	Fusobacterium_polymorphum.GCF_001457555_1	OK_nc	0.59	7.89E-14	2.88E-16
sp14217355	Fusobacterium_hwasookii.GCF_014217355_1	OK_nc	0.59	8.40E-14	3.50E-16
sp381525	Peptostreptococcus_anaerobius.GCF_000381525_1	OK_nc	0.58	1.59E-13	7.54E-16
sp2573625	Fusobacterium_nucleatum.GCF_002573625_1	OK_nc	0.58	1.59E-13	8.63E-16
sp493815	Fusobacterium_nucleatum.GCF_000493815_1	OK_nc	0.58	1.59E-13	9.11E-16
sp174815	Porphyromonas_endodontalis.GCF_000174815_1	OK_nc	0.58	3.18E-13	2.00E-15
sp3859915	Fusobacterium_canifelinum.GCF_003859915_1	OK_nc	0.57	3.18E-13	2.29E-15
sp7325	Fusobacterium_nucleatum.GCA_000007325_1	OK_nc	0.57	3.18E-13	2.32E-15
sp8633215	Fusobacterium_nucleatum.GCF_008633215_1	OK_nc	0.57	6.18E-13	4.83E-15
sp182945	Fusobacterium_vincentii.GCA_000182945_1	OK_nc	0.56	8.65E-13	7.21E-15
sp163895	Filifactor_alocis.GCF_000163895_2	OK_nc	0.56	8.79E-13	7.78E-15
sp214475	Parvimonas_sp000214475.GCF_000214475_1	OK_nc	0.56	1.35E-12	1.33E-14
sp223315	Parvimonas_sp000223315.GCF_000223315_1	OK_nc	0.56	1.35E-12	1.28E-14
sp4006635	Fusobacterium.GCF_004006635_1	OK_nc	0.55	1.71E-12	1.78E-14
sp900548415	Porphyromonas_sp900548415.GCA_900548415_1	OK_nc	0.54	6.18E-12	6.76E-14
sp160475	Fusobacterium_periodonticum.GCF_000160475_1	OK_nc	0.54	8.62E-12	9.88E-14
sp220235	Prevotella_nigrescens.GCF_000220235_1	OK_nc	0.53	2.21E-11	2.65E-13
sp158275	Fusobacterium_animalis.GCF_000158275_2	OK_nc	0.52	5.03E-11	6.29E-13
sp900476045	Gemella_morbillorum.GCF_900476045_1	OK_nc	0.50	4.97E-10	6.47E-12

Custom ID	GTDB + Genome Assembly Species	Deconfounding status	Ds	p value	q value
sp900759325	Peptostreptococcus_sp900759325.GCA_900759325_1	OK_nc	0.48	3.91E-09	5.29E-11
sp2763915	Fusobacterium_periodonticum.GCF_002763915_1	OK_nc	0.47	4.19E-09	5.90E-11
sp425005	Bulleidia_moorei.GCF_000425005_1	OK_nc	0.47	5.13E-09	7.49E-11
sp1553085	Parvimonas_sp001553085.GCF_001553085_1	OK_nc	0.47	5.43E-09	8.19E-11
sp900538385	Porphyromonas_sp900538385.GCA_900538385_1	OK_nc	0.46	6.58E-09	1.03E-10
sp372405	Porphyromonas.GCF_000372405_1	OK_sd	0.45	2.83E-08	4.72E-10
sp163935	Fusobacterium_periodonticum.GCF_000163935_1	OK_nc	0.45	4.66E-08	8.26E-10
sp250875	Sutterella_parvirubra.GCF_000250875_1	OK_nc	0.44	8.45E-08	1.54E-09
sp613945	Prevotella_falsenii.GCF_000613945_1	OK_nc	0.42	4.13E-07	7.95E-09
sp1717505	Allisonella_pneumosintes.GCF_001717505_1	OK_nc	0.42	4.91E-07	9.71E-09
sp2761955	Fusobacterium_pseudoperiodonticum.GCF_002761955_1	OK_nc	0.41	5.78E-07	1.18E-08
sp900539765	Porphyromonas_sp900539765.GCA_900539765_1	OK_nc	0.41	2.40E-07	4.49E-09
sp1881565	Eisenbergiella_tayi.GCF_001881565_1	OK_nc	0.41	8.77E-07	1.83E-08
sp174775	Porphyromonas_uenonis.GCF_000174775_1	OK_nc	0.40	1.20E-06	2.56E-08
sp435055	PeH17_sp000435055.GCA_000435055_1	OK_nc	0.40	1.48E-06	3.23E-08
sp525775	Mogibacterium_timidum.GCF_000525775_1	OK_nc	0.40	1.64E-06	3.68E-08
sp220255	Prevotella_pallens.GCF_000220255_1	OK_nc	0.40	1.87E-06	4.27E-08
sp900539155	Porphyromonas_sp900539155.GCA_900539155_1	OK_sd	0.39	2.29E-06	5.49E-08
sp614025	Prevotella_aurantiaca.GCF_000614025_1	OK_nc	0.39	3.87E-06	9.48E-08
sp482365	Porphyromonas_uenonis.GCF_000482365_1	OK_nc	0.38	4.89E-06	1.22E-07
sp188175	Phascolarctobacterium.GCF_000188175_1	OK_nc	0.37	1.03E-05	2.72E-07
sp3977605	Prevotella_koreensis.GCF_003977605_1	OK_nc	0.37	1.12E-05	3.03E-07
sp900635025	Morganella_morganii.GCA_900635025_1	OK_nc	0.37	5.29E-06	1.38E-07
sp159995	Alloprevotella_tannerae.GCF_000159995_1	OK_nc	0.36	1.86E-05	5.51E-07
sp3538135	CAG-177_sp003538135.GCA_003538135_1	OK_sd	0.36	1.86E-05	5.50E-07
sp2998925	Mogibacterium_diversum.GCF_002998925_1	OK_nc	0.36	2.79E-05	9.01E-07
sp212375	Porphyromonas_asaccharolytica.GCF_000212375_1	OK_sd	0.36	2.69E-05	8.54E-07
sp900554555	Bulleidia_sp900554555.GCA_900554555_1	OK_nc	0.35	3.32E-05	1.09E-06
sp900187105	Eikenella_corrodens.GCF_900187105_1	OK_nc	0.35	1.73E-05	4.96E-07
sp900543475	UBA5394_sp003150565.GCA_900543475_1	OK_nc	0.35	3.80E-05	1.27E-06
sp3096415	Victivallis_vadensis.GCF_003096415_1	OK_nc	0.35	2.48E-05	7.75E-07
sp435555	CAG-83_sp000435555.GCA_000435555_1	OK_nc	0.35	4.79E-05	1.65E-06
sp4557245	SFEL01_sp004557245.GCA_004557245_1	OK_sd	0.34	6.36E-05	2.32E-06
sp437635	CAG-521_sp000437635.GCA_000437635_1	OK_nc	0.34	5.99E-05	2.15E-06
sp2438685	CAG-177_sp002438685.GCA_002438685_1	OK_sd	0.34	5.87E-05	2.08E-06
sp318095	F0040_sp000318095.GCF_000318095_2	OK_nc	0.34	9.26E-05	3.47E-06
sp155955	Mitsuokella_multacida.GCF_000155955_1	OK_nc	0.34	8.37E-05	3.10E-06
sp3514385	CAG-177_sp003514385.GCA_003514385_1	OK_sd	0.33	1.21E-04	4.72E-06
sp900317525	ER4_sp900317525.GCA_900317525_1	OK_sd	0.33	1.65E-04	6.68E-06
sp900120395	Porphyromonas.GCF_900120395_1	OK_nc	0.32	1.37E-04	5.43E-06
sp900540495	UMGS1241_sp900540495.GCA_900540495_1	OK_nc	0.32	1.89E-04	7.77E-06
sp239695	Actinomyces_graevenitzii.GCF_000239695_1	OK_nc	0.32	2.27E-04	9.68E-06
sp377625	Prevotella_veroralis.GCF_000377625_1	OK_nc	0.32	2.33E-04	1.03E-05

Custom ID	GTDB + Genome Assembly Species	Deconfounding status	Ds	p value	q value
sp20225	Akkermansia_muciniphila.GCF_000020225_1	OK_nc	0.32	2.51E-04	1.14E-05
sp509105	Muricomes_sp000509105.GCF_000509105_1	OK_nc	0.32	2.51E-04	1.14E-05
sp900541915	Phascolarctobacterium.GCA_900541915_1	OK_sd	0.32	1.65E-04	6.71E-06
sp900142645	Eubacterium_callanderi.GCF_900142645_1	OK_nc	0.32	3.03E-04	1.43E-05
sp900110295	Peptostreptococcus_russellii.GCF_900110295_1	OK_nc	0.31	1.11E-04	4.20E-06
sp613445	Prevotella_shahii.GCF_000613445_1	OK_nc	0.31	3.03E-04	1.44E-05
sp4558145	Fournierella_sp004558145.GCA_004558145_1	OK_sd	0.31	3.99E-04	2.00E-05
sp900546675	Porphyromonas_sp900546675.GCA_900546675_1	OK_sd	0.31	2.87E-04	1.33E-05
sp156375	Desulfovibrio_piger.GCF_000156375_1	OK_nc	0.31	4.66E-04	2.40E-05
sp10505	Porphyromonas_gingivalis.GCF_000010505_1	OK_nc	0.31	4.72E-04	2.46E-05
sp4556155	SFMI01_sp004556155.GCA_004556155_1	OK_sd	0.30	5.24E-04	2.78E-05
sp4134775	Borkfalkia_ceftriaxoniphila.GCF_004134775_1	OK_nc	0.30	1.12E-04	4.33E-06
sp467895	Prevotella_sp000467895.GCF_000467895_1	OK_nc	0.30	5.80E-04	3.16E-05
sp900763675	UBA738_sp900763675.GCA_900763675_1	OK_nc	0.30	5.80E-04	3.17E-05
sp900554935	Porphyromonas.GCA_900554935_1	OK_nc	0.30	3.50E-04	1.70E-05
sp432135	CAG-170_sp000432135.GCA_000432135_1	OK_sd	0.30	6.42E-04	3.54E-05
sp382385	Prevotella_maculosa.GCF_000382385_1	OK_nc	0.30	7.16E-04	4.03E-05
sp3010495	Megasphaera_elsdenii.GCF_003010495_1	OK_nc	0.30	5.24E-04	2.76E-05
sp1262015	Prevotella_fusca.GCF_001262015_1	OK_nc	0.30	8.10E-04	4.60E-05
sp190535	Odoribacter_splanchnicus.GCF_000190535_1	OK_sd	0.30	8.27E-04	4.74E-05
sp435195	Negativibacillus_sp000435195.GCA_000435195_1	OK_sd	0.29	8.50E-04	4.96E-05
sp1552775	Porphyromonas_sp001552775.GCF_001552775_1	OK_nc	0.29	8.31E-04	4.81E-05
sp900095835	F0040_sp900095835.GCF_900095835_1	OK_nc	0.29	9.21E-04	5.52E-05
sp2404795	CAG-170_sp002404795.GCA_002404795_1	OK_sd	0.29	1.13E-03	7.20E-05
sp2451755	CAG-177_sp002451755.GCA_002451755_1	OK_sd	0.29	1.08E-03	6.68E-05
sp900548625	CAG-170_sp900548625.GCA_900548625_1	OK_sd	0.29	1.22E-03	7.87E-05
sp392875	Enterococcus_faecalis.GCF_000392875_1	OK_nc	0.29	9.41E-04	5.74E-05
sp1916855	CAG-83_sp001916855.GCA_001916855_1	OK_sd	0.29	1.32E-03	8.63E-05
sp231275	Alistipes.GCF_000231275_1	OK_sd	0.28	1.34E-03	8.96E-05
sp160675	Granulicatella_adiacens.GCF_000160675_1	OK_nc	0.28	1.36E-03	9.19E-05
sp4553625	UBA5446_sp004553625.GCA_004553625_1	OK_nc	0.28	1.37E-03	9.36E-05
sp296385	Lachnoanaerobaculum_sp000296385.GCF_000296385_1	OK_sd	0.28	1.42E-03	9.86E-05
sp969835	Parabacteroides_goldsteinii.GCF_000969835_1	OK_sd	0.28	1.45E-03	1.01E-04
sp900087055	Plesiomonas_shigelloides.GCF_900087055_1	OK_nc	0.28	2.30E-04	1.01E-05
sp2160955	Gemmiger.GCF_002160955_1	OK_nc	0.28	1.50E-03	1.06E-04
sp1697145	Streptococcus_anginosus.GCF_001697145_1	OK_nc	0.28	1.72E-03	1.23E-04
sp208405	Selenomonas_sputigena.GCF_000208405_1	OK_nc	0.28	1.69E-03	1.20E-04
sp2439735	CAG-238_sp002439735.GCA_002439735_1	OK_sd	0.28	2.03E-03	1.47E-04
sp900554275	CAG-83_sp900554275.GCA_900554275_1	OK_nc	0.27	2.14E-03	1.58E-04
sp3340345	Paraeggerthella_hongkongensis.GCF_003340345_1	OK_nc	0.27	2.20E-03	1.65E-04
sp900636475	Streptococcus_anginosus.GCF_900636475_1	OK_nc	0.27	2.20E-03	1.65E-04
sp900547745	CAG-83_sp900547745.GCA_900547745_1	OK_sd	0.27	2.27E-03	1.71E-04
sp900754805	CAG-238_sp900754805.GCF_900754805_1	OK_sd	0.27	2.78E-03	2.13E-04

Custom ID	GTDB + Genome Assembly Species	Deconfounding status	Ds	p value	q value
sp436735	CAG-170_sp000436735.GCA_000436735_1	OK_sd	0.27	2.98E-03	2.29E-04
sp900548425	CAG-302_sp900548425.GCA_900548425_1	OK_sd	0.27	1.09E-03	6.83E-05
sp900752115	Hydrogeniiclostridium_sp900752115.GCF_900752115_1	OK_nc	0.26	3.60E-03	2.90E-04
sp427365	Rikenella_microfusus.GCF_000427365_1	OK_nc	0.26	3.62E-03	2.94E-04
sp900549395	CAG-83_sp900549395.GCA_900549395_1	OK_sd	0.26	3.54E-03	2.82E-04
sp902373545	Pauljensenia_sp902373545.GCA_902373545_1	OK_sd	0.26	3.66E-03	2.99E-04
sp14385435	F0040_sp003639005.GCF_014385435_1	OK_nc	0.26	3.72E-03	3.06E-04
sp900552845	Angelakisella_sp900552845.GCA_900552845_1	OK_sd	0.26	3.96E-03	3.31E-04
sp633515	Morganella_morganii.GCF_000633515_1	OK_nc	0.26	1.38E-03	9.49E-05
sp1917175	51-20_sp001917175.GCA_001917175_1	OK_nc	0.26	2.10E-03	1.53E-04
sp8016795	Prevotella_brunnea.GCF_008016795_1	OK_sd	0.26	4.57E-03	3.89E-04
sp2939185	Enterobacter_sichuanensis.GCF_002939185_1	OK_nc	0.26	4.57E-03	3.91E-04
sp900762555	Duodenibacillus_sp900762555.GCA_900762555_1	OK_nc	0.26	4.75E-03	4.08E-04
sp434635	CAG-110_sp000434635.GCA_000434635_1	OK_sd	0.26	4.80E-03	4.19E-04
sp4557855	Angelakisella_sp004557855.GCA_004557855_1	OK_sd	0.26	4.80E-03	4.22E-04
sp466485	Clostridium.GCF_000466485_1	OK_nc	0.26	4.77E-03	4.12E-04
sp3432035	Lacrimispora_indicum.GCF_003432035_1	OK_nc	0.26	4.80E-03	4.22E-04
sp374505	Alistipes_onderdonkii.GCF_000374505_1	OK_sd	0.26	5.05E-03	4.49E-04
sp14647335	Butyricimonas_faecihominis.GCA_014647335_1	OK_sd	0.25	5.21E-03	4.69E-04
sp599605	Prevotella_sp000599605.GCF_000599605_1	OK_nc	0.25	5.22E-03	4.78E-04
sp900548615	CAG-83_sp900548615.GCA_900548615_1	OK_sd	0.25	5.31E-03	4.90E-04
sp900754495	UMGS1293_sp900754495.GCF_900754495_1	OK_nc	0.25	5.37E-03	4.98E-04
sp3865035	Anaerotignum_faecicola.GCF_003865035_1	OK_sd	0.25	5.56E-03	5.24E-04
sp479005	Prevotella_sp000479005.GCF_000479005_1	OK_nc	0.25	5.38E-03	5.04E-04
sp3522945	UBA738_sp003522945.GCA_003522945_1	OK_sd	0.25	5.63E-03	5.41E-04
sp8801935	Comamonas_kerstersii.GCF_008801935_1	OK_nc	0.25	1.34E-03	8.95E-05
sp900546615	UBA5446_sp900546615.GCA_900546615_1	OK_nc	0.25	5.63E-03	5.45E-04
sp900543485	UBA1191_sp900543485.GCA_900543485_1	OK_nc	0.25	5.58E-03	5.32E-04
sp900540415	Prevotella_sp900540415.GCA_900540415_1	OK_nc	0.25	5.58E-03	5.32E-04
sp2329575	CAG-521_sp002329575.GCA_002329575_1	OK_nc	0.25	3.72E-03	3.08E-04
sp900768135	ER4_sp900768135.GCA_900768135_1	OK_sd	0.25	5.87E-03	5.79E-04
sp14287895	Flavonifractor_sp900549795.GCF_014287895_1	OK_sd	0.25	5.87E-03	5.84E-04
sp4154955	Intestinimonas_butyriciproducens.GCF_004154955_1	OK_nc	0.25	5.87E-03	5.81E-04
sp2998355	Victivallis_sp002998355.GCF_002998355_1	OK_nc	0.25	5.63E-03	5.45E-04
sp900543285	Fournierella_sp900543285.GCA_900543285_1	OK_nc	0.25	6.01E-03	6.07E-04
sp4556165	PeH17_sp004556165.GCA_004556165_1	OK_sd	0.25	5.87E-03	5.79E-04
sp210715	Fretibacterium_fastidiosum.GCA_000210715_1	OK_nc	0.25	6.06E-03	6.17E-04
sp14195585	Alloprevotella_rava.GCF_014195585_1	OK_nc	0.25	6.09E-03	6.32E-04
sp900552375	ER4_sp900552375.GCA_900552375_1	OK_nc	0.25	6.06E-03	6.21E-04
sp900552145	Collinsella_sp900552145.GCA_900552145_1	OK_sd	0.25	6.06E-03	6.22E-04
sp14306095	UBA1191_sp900549125.GCA_014306095_1	OK_nc	0.25	6.24E-03	6.60E-04
sp2438575	CAG-83_sp002438575.GCA_002438575_1	OK_nc	0.25	6.12E-03	6.40E-04
sp613345	Prevotella_disiens.GCF_000613345_1	OK_nc	0.25	6.59E-03	7.11E-04

Custom ID	GTDB + Genome Assembly Species	Deconfounding status	Ds	p value	q value
sp4555205	CAG-83_sp004555205.GCA_004555205_1	OK_sd	0.25	6.59E-03	7.14E-04
sp900547385	Angelakisella_sp900547385.GCA_900547385_1	OK_nc	0.25	6.59E-03	7.10E-04
sp900550805	UMGS1338_sp900550805.GCA_900550805_1	OK_nc	0.25	6.12E-03	6.39E-04
sp900760125	UBA1409_sp900760125.GCF_900760125_1	OK_sd	0.25	6.72E-03	7.35E-04
sp900759385	UBA5446_sp900759385.GCA_900759385_1	OK_nc	0.24	6.85E-03	7.52E-04
sp4556455	SFGY01_sp004556455.GCA_004556455_1	OK_sd	0.24	6.99E-03	7.72E-04
sp7679425	Streptomyces_incarnatus.GCF_007679425_1	OK_nc	0.24	7.12E-03	7.94E-04
sp900556015	CAG-83_sp900556015.GCA_900556015_1	OK_sd	0.24	7.68E-03	8.66E-04
sp900759575	Acutalibacter_sp900759575.GCF_900759575_1	OK_nc	0.24	7.68E-03	8.61E-04
sp900550905	Victivallis_sp900550905.GCA_900550905_1	OK_nc	0.24	5.65E-03	5.50E-04
sp900095825	Acidaminococcus_massiliensis.GCF_900095825_1	OK_sd	0.24	7.68E-03	8.68E-04
sp900540885	Prevotellamassilia_sp900540885.GCA_900540885_1	OK_nc	0.24	7.81E-03	8.90E-04
sp4557655	CAG-83_sp004557655.GCA_004557655_1	OK_sd	0.24	7.80E-03	8.86E-04
sp900552775	UMGS1540_sp900552775.GCA_900552775_1	OK_nc	0.24	8.08E-03	9.38E-04
sp900554615	CAG-302_sp900554615.GCA_900554615_1	OK_sd	0.24	3.11E-03	2.42E-04
sp900148495	Negativibacillus_massiliensis.GCF_900148495_1	OK_sd	0.24	8.15E-03	9.51E-04
sp435975	CAG-83_sp000435975.GCA_000435975_1	OK_nc	0.24	8.26E-03	9.72E-04
sp1940855	Phil1_sp001940855.GCA_001940855_1	OK_sd	0.24	8.36E-03	9.88E-04
sp900542005	UMGS416_sp900542005.GCA_900542005_1	OK_sd	0.24	8.23E-03	9.64E-04
sp900184965	Akkermansia_muciniphila.GCF_900184965_1	OK_nc	0.24	7.87E-03	9.06E-04
sp900542245	CAG-238_sp900542245.GCA_900542245_1	OK_sd	0.24	8.46E-03	1.00E-03
sp900552475	CAG-83_sp900552475.GCA_900552475_1	OK_nc	0.24	8.75E-03	1.06E-03
sp900545585	CAG-83_sp900545585.GCA_900545585_1	OK_sd	0.24	8.66E-03	1.04E-03
sp900547315	UBA1777_sp900547315.GCA_900547315_1	OK_sd	0.24	8.73E-03	1.05E-03
sp2437735	ER4_sp002437735.GCA_002437735_1	OK_sd	0.24	8.66E-03	1.04E-03
sp900551415	CAG-238_sp900551415.GCA_900551415_1	OK_sd	0.24	8.91E-03	1.08E-03
sp4557565	Sodaliphilus_sp004557565.GCA_004557565_1	OK_sd	0.24	9.46E-03	1.17E-03
sp900550165	ER4_sp900550165.GCA_900550165_1	OK_sd	0.24	9.19E-03	1.12E-03
sp900555735	CAG-83_sp900555735.GCA_900555735_1	OK_sd	0.24	9.60E-03	1.20E-03
sp900113995	D5_sp900113995.GCF_900113995_1	OK_sd	0.24	9.61E-03	1.21E-03
sp900545495	CAG-83_sp900545495.GCA_900545495_1	OK_sd	0.24	9.60E-03	1.20E-03
sp900545215	UBA3388_sp900545215.GCA_900545215_1	OK_sd	0.24	9.21E-03	1.13E-03
sp25565	Enterobacter_cloacae.GCF_000025565_1	OK_nc	0.23	9.84E-03	1.24E-03
sp3019695	Fusobacterium.GCF_003019695_1	OK_nc	0.23	5.22E-03	4.73E-04
sp900546295	ER4_sp900546295.GCA_900546295_1	OK_sd	0.23	9.89E-03	1.25E-03
sp518545	Prevotella_seregens.GCF_000518545_1	OK_nc	0.23	1.02E-02	1.30E-03
sp1729805	Enterobacter_roggenkampii.GCF_001729805_1	OK_nc	0.23	1.01E-02	1.28E-03
sp900539755	Alistipes.GCA_900539755_1	OK_nc	0.23	1.05E-02	1.35E-03
sp786575	Tidjanibacter_inops.GCA_000786575_1	OK_nc	0.23	1.06E-02	1.37E-03
sp900546315	UMGS874_sp900546315.GCA_900546315_1	OK_sd	0.23	1.05E-02	1.35E-03
sp900549045	UBA1829_sp900549045.GCA_900549045_1	OK_nc	0.23	1.06E-02	1.37E-03
sp434215	UBA11471_sp000434215.GCA_000434215_1	OK_nc	0.23	1.10E-02	1.44E-03
sp900555605	UMGS1851_sp900555605.GCA_900555605_1	OK_nc	0.23	1.09E-02	1.42E-03

Custom ID	GTDB + Genome Assembly Species	Deconfounding status	Ds	p value	q value
sp900542395	SFFH01_sp900542395.GCA_900542395_1	OK_sd	0.23	1.09E-02	1.43E-03
sp900553545	CAG-170_sp900553545.GCA_900553545_1	OK_sd	0.23	1.17E-02	1.55E-03
sp4556545	Firm-11_sp004556545.GCA_004556545_1	OK_sd	0.23	1.16E-02	1.53E-03
sp900155735	Intestinibacillus_massiliensis.GCF_900155735_1	OK_nc	0.23	1.30E-02	1.75E-03
sp13333285	Prevotella_sp013333285.GCA_013333285_2	OK_nc	0.23	1.20E-02	1.60E-03
sp4556345	Evtepia_sp004556345.GCA_004556345_1	OK_sd	0.23	1.36E-02	1.85E-03
sp900626285	Flavonifractor_massiliensis.GCF_900626285_1	OK_nc	0.23	1.36E-02	1.85E-03
sp900752445	UBA5446_sp900752445.GCA_900752445_1	OK_nc	0.23	1.38E-02	1.89E-03
sp902168225	Sediminibacterium_sp902168225.GCA_902168225_1	OK_nc	0.23	1.38E-02	1.89E-03
sp900548125	SFFH01_sp900548125.GCA_900548125_1	OK_nc	0.22	1.51E-02	2.13E-03
sp154465	Alistipes_putredinis.GCF_000154465_1	OK_sd	0.22	1.52E-02	2.17E-03
sp900549955	UMGS1241_sp900549955.GCA_900549955_1	OK_nc	0.22	1.50E-02	2.11E-03
sp1275135	Prevotella_rara.GCF_001275135_1	OK_nc	0.22	1.58E-02	2.27E-03
sp900549885	UBA9475_sp900549885.GCA_900549885_1	OK_nc	0.22	1.58E-02	2.28E-03
sp3539495	CAG-83_sp003539495.GCA_003539495_1	OK_sd	0.22	1.59E-02	2.32E-03
sp900544405	CAG-110_sp900544405.GCA_900544405_1	OK_nc	0.22	1.60E-02	2.36E-03
sp1940805	Methanocorpusculum_sp001940805.GCA_001940805_1	OK_nc	0.22	9.41E-04	5.71E-05
sp434055	CAG-488_sp000434055.GCA_000434055_1	OK_nc	0.22	1.60E-02	2.33E-03
sp437795	CAG-279_sp000437795.GCA_000437795_1	OK_sd	0.22	1.61E-02	2.39E-03
sp2900365	Escherichia_marmotae.GCF_002900365_1	OK_nc	0.22	1.61E-02	2.39E-03
sp900547305	UBA737_sp900547305.GCA_900547305_1	OK_sd	0.22	1.60E-02	2.35E-03
sp900543295	UBA866_sp900543295.GCA_900543295_1	OK_nc	0.22	1.64E-02	2.45E-03
sp184945	Prevotella_buccae.GCF_000184945_1	OK_nc	0.22	1.65E-02	2.52E-03
sp900544885	Phascolarctobacterium.GCA_900544885_1	OK_nc	0.22	1.65E-02	2.52E-03
sp900754755	HGM11588_sp900754755.GCF_900754755_1	OK_sd	0.22	1.50E-02	2.12E-03
sp900757415	CAG-83_sp900757415.GCA_900757415_1	OK_sd	0.22	1.72E-02	2.69E-03
sp900543215	UBA737_sp900543215.GCA_900543215_1	OK_sd	0.22	1.71E-02	2.61E-03
sp321205	Alistipes.GCF_000321205_1	OK_nc	0.22	1.72E-02	2.64E-03
sp297775	Sutterella_wadsworthensis.GCF_000297775_1	OK_nc	0.22	1.72E-02	2.65E-03
sp900545525	Prevotella_sp900545525.GCA_900545525_1	OK_nc	0.22	1.72E-02	2.69E-03
sp900626145	Intestinimonas_timonensis.GCF_900626145_1	OK_nc	0.22	1.72E-02	2.69E-03
sp437375	CAG-841_sp000437375.GCA_000437375_1	OK_sd	0.22	1.53E-02	2.18E-03
sp3487665	CAG-83_sp003487665.GCA_003487665_1	OK_sd	0.22	1.77E-02	2.83E-03
sp900543805	Sutterella_sp900543805.GCA_900543805_1	OK_nc	0.22	1.77E-02	2.80E-03
sp900545275	Sutterella_sp900545275.GCA_900545275_1	OK_nc	0.22	1.77E-02	2.82E-03
sp900542445	SFFH01_sp900542445.GCA_900542445_1	OK_sd	0.22	1.74E-02	2.75E-03
sp2404605	UBA738_sp002404605.GCA_002404605_1	OK_sd	0.22	1.80E-02	2.89E-03
sp2222615	Bacteroides_caccae.GCF_002222615_2	OK_nc	0.22	1.81E-02	2.95E-03
sp4556925	SFFI01_sp004556925.GCA_004556925_1	OK_nc	0.22	1.73E-02	2.73E-03
sp900538905	Duodenibacillus_sp900538905.GCA_900538905_1	OK_nc	0.22	1.80E-02	2.88E-03
sp900556145	ER4_sp900556145.GCA_900556145_1	OK_sd	0.22	1.81E-02	2.95E-03
sp14384705	NSJ-40_sp014384705.GCA_014384705_1	OK_nc	0.22	1.81E-02	2.93E-03
sp900751035	CAG-170_sp900751035.GCA_900751035_1	OK_sd	0.22	1.86E-02	3.07E-03

Custom ID	GTDB + Genome Assembly Species	Deconfounding status	Ds	p value	q value
sp900554455	Collinsella_sp900554455.GCA_900554455_1	OK_sd	0.22	1.83E-02	3.00E-03
sp900548155	Alistipes_sp900548155.GCA_900548155_1	OK_sd	0.22	1.83E-02	3.00E-03
sp735365	Kluyvera_ascorbata.GCF_000735365_1	OK_nc	0.22	1.81E-02	2.95E-03
sp900555855	UBA4644_sp900555855.GCA_900555855_1	OK_sd	0.22	1.85E-02	3.04E-03
sp14287275	Pygmaiobacter_sp014287275.GCF_014287275_1	OK_sd	0.21	1.88E-02	3.11E-03
sp3343845	UMGS902_sp003343845.GCA_003343845_1	OK_sd	0.21	1.62E-02	2.41E-03
sp4309735	Desulfovibrio_legallii.GCF_004309735_1	OK_nc	0.21	1.58E-02	2.28E-03
sp900553265	UMGS1603_sp900553265.GCA_900553265_1	OK_sd	0.21	2.00E-02	3.38E-03
sp900542255	Barnesiella_sp900542255.GCA_900542255_1	OK_nc	0.21	2.00E-02	3.37E-03
sp900546885	Ruthenibacterium_sp900546885.GCA_900546885_1	OK_nc	0.21	2.15E-02	3.71E-03
sp900549755	UBA737_sp900549755.GCA_900549755_1	OK_sd	0.21	2.07E-02	3.51E-03
sp900550585	CAG-83_sp900550585.GCA_900550585_1	OK_sd	0.21	2.18E-02	3.78E-03
sp900763705	ER4_sp900763705.GCA_900763705_1	OK_nc	0.21	2.26E-02	3.95E-03
sp519105	Butyricimonas_virosa.GCF_000519105_1	OK_sd	0.21	2.24E-02	3.90E-03
sp3522105	ER4_sp003522105.GCA_003522105_1	OK_sd	0.21	2.36E-02	4.17E-03
sp14218705	Kluyvera_sichuanensis.GCF_014218705_1	OK_nc	0.21	2.29E-02	4.01E-03
sp900551995	CAG-83_sp900551995.GCA_900551995_1	OK_sd	0.21	2.36E-02	4.19E-03
sp900553525	UMGS1623_sp900553525.GCA_900553525_1	OK_sd	0.21	2.36E-02	4.15E-03
sp900763685	Dysosmobacter_sp900763685.GCA_900763685_1	OK_nc	0.21	2.38E-02	4.24E-03
sp900551355	CAG-83_sp900551355.GCA_900551355_1	OK_sd	0.21	2.30E-02	4.04E-03
sp900319575	Desulfovibrio_sp900319575.GCA_900319575_1	OK_nc	0.21	1.45E-02	2.02E-03
sp438075	CAG-460_sp000438075.GCA_000438075_1	OK_sd	0.21	6.09E-03	6.32E-04
sp902388735	CAG-83_sp902388735.GCA_902388735_1	OK_sd	0.21	2.47E-02	4.42E-03
sp900078395	Anaerotruncus_rubiinfantis.GCF_900078395_1	OK_nc	0.21	2.47E-02	4.43E-03
sp153925	Mediterraneibacter_torques.GCF_000153925_1	OK_nc	0.21	2.48E-02	4.47E-03
sp208585	Escherichia_sp000208585.GCF_000208585_1	OK_nc	0.21	2.59E-02	4.73E-03
sp6542665	Alistipes_communis.GCF_006542665_1	OK_sd	0.21	2.62E-02	4.82E-03
sp1940825	UBA9732_sp001940825.GCA_001940825_1	OK_sd	0.21	2.55E-02	4.63E-03
sp5845105	GCA-900066905_sp900066905.GCF_005845105_1	OK_nc	0.21	2.58E-02	4.70E-03
sp3477405	AM07-15_sp003477405.GCA_003477405_1	OK_nc	0.20	2.73E-02	5.08E-03
sp900754775	HGM11575_sp900754775.GCF_900754775_1	OK_sd	0.20	2.63E-02	4.85E-03
sp26225	Escherichia_fergusonii.GCF_000026225_1	OK_nc	0.20	2.75E-02	5.15E-03
sp2361215	CAG-485_sp002361215.GCA_002361215_1	OK_nc	0.20	2.75E-02	5.14E-03
sp900756715	CAG-83_sp900756715.GCA_900756715_1	OK_sd	0.20	2.76E-02	5.20E-03
sp900544945	CAG-110_sp900544945.GCA_900544945_1	OK_sd	0.20	2.75E-02	5.13E-03
sp900313295	CAG-83_sp900313295.GCA_900313295_1	OK_sd	0.20	2.77E-02	5.21E-03
sp900761125	UMGS1537_sp900552695.GCF_900761125_1	OK_nc	0.20	2.65E-02	4.90E-03
sp12728015	Thermoclostridium.GCA_012728015_1	OK_nc	0.20	2.08E-02	3.57E-03
sp900546075	CAG-110_sp900546075.GCA_900546075_1	OK_sd	0.20	2.78E-02	5.27E-03
sp4558205	Pyramidobacter_piscolens.GCA_004558205_1	OK_nc	0.20	2.84E-02	5.53E-03
sp2405565	CAG-841_sp002405565.GCA_002405565_1	OK_nc	0.20	2.36E-02	4.20E-03
sp4554585	Evtepia_sp004554585.GCA_004554585_1	OK_nc	0.20	2.84E-02	5.50E-03
sp900548905	Eisenbergiella_sp900548905.GCA_900548905_1	OK_nc	0.20	2.84E-02	5.52E-03

Custom ID	GTDB + Genome Assembly Species	Deconfounding status	Ds	p value	q value
sp900544295	UBA5446_sp900544295.GCA_900544295_1	OK_nc	0.20	2.84E-02	5.55E-03
sp2950215	Escherichia_flexneri.GCF_002950215_1	OK_nc	0.20	2.84E-02	5.53E-03
sp435615	CAG-238_sp000435615.GCA_000435615_1	OK_sd	0.20	2.85E-02	5.59E-03
sp1916715	F23-B02_sp001916715.GCA_001916715_1	OK_nc	0.20	2.84E-02	5.55E-03
sp16525	Methanobrevibacter.GCF_000016525_1	OK_nc	0.20	9.51E-03	1.18E-03
sp900128465	Pauljensenia_bouchesdurhonensis.GCF_900128465_1	OK_sd	0.20	2.89E-02	5.73E-03
sp2437575	CAG-170_sp002437575.GCA_002437575_1	OK_sd	0.20	2.86E-02	5.63E-03
sp900556525	Ruminiclostridium.GCA_900556525_1	OK_nc	0.20	2.95E-02	5.87E-03
sp900543625	CAG-103_sp900543625.GCA_900543625_1	OK_sd	0.20	2.96E-02	5.93E-03
sp900544755	Collinsella_sp900544755.GCA_900544755_1	OK_sd	0.20	2.87E-02	5.69E-03
sp900555665	CAG-170_sp900555665.GCA_900555665_1	OK_sd	0.20	2.87E-02	5.68E-03
sp3467125	Caecibacter_sp003467125.GCF_003467125_1	OK_nc	0.20	2.99E-02	5.99E-03
sp762405	Prevotella_timonensis.GCF_000762405_1	OK_nc	0.20	3.03E-02	6.17E-03
sp11881725	Escherichia_coli.GCF_011881725_1	OK_nc	0.20	2.99E-02	6.01E-03
sp3149245	Muricomes_oroticus.GCF_003149245_1	OK_nc	0.20	2.99E-02	6.04E-03
sp9696265	Desulfovibrio_sp900540515.GCF_009696265_1	OK_nc	0.20	3.05E-02	6.26E-03
sp2160015	Anaerofilum_sp002160015.GCF_002160015_1	OK_nc	0.20	3.12E-02	6.46E-03
sp2161215	Flavonifractor_sp002161215.GCF_002161215_1	OK_nc	0.20	3.09E-02	6.39E-03
sp765235	ER4_sp000765235.GCF_000765235_1	OK_sd	0.20	3.06E-02	6.30E-03
sp55555000226395	Penicillium_rubens_Wisconsin_54-1255.GCF_000226395_1	OK_nc	0.20	3.16E-02	6.60E-03
sp900546415	CAG-110_sp900546415.GCA_900546415_1	OK_sd	0.20	3.18E-02	6.66E-03
sp1808555	Porphyromonas.GCF_001808555_1	OK_nc	0.20	3.01E-02	6.11E-03
sp900545935	UMGS911_sp900545935.GCA_900545935_1	OK_sd	0.20	3.05E-02	6.23E-03
sp3043945	Prevotella_sp003043945.GCF_003043945_1	OK_nc	0.20	3.22E-02	6.75E-03
sp900553255	UMGS1591_sp900553255.GCA_900553255_1	OK_nc	0.20	3.15E-02	6.56E-03
sp14384785	UBA9506_sp003506415.GCA_014384785_1	OK_nc	0.20	3.25E-02	6.90E-03
sp902373525	Bilophila_sp902373525.GCA_902373525_1	OK_nc	0.20	3.23E-02	6.82E-03
sp436695	Prevotella_sp000436695.GCA_000436695_1	OK_sd	0.20	3.26E-02	6.93E-03
sp900545515	CAG-110_sp000435995.GCA_900545515_1	OK_nc	0.20	3.26E-02	6.94E-03
sp4557075	F23-B02_sp004557075.GCA_004557075_1	OK_nc	0.20	3.23E-02	6.81E-03
sp4803915	Duncaniella_dubosii.GCF_004803915_1	OK_nc	0.20	3.27E-02	6.97E-03
sp900551265	UMGS1384_sp900551265.GCA_900551265_1	OK_nc	0.20	3.25E-02	6.88E-03
sp4555735	Acidaminococcus_fermentans.GCA_004555735_1	OK_nc	0.20	3.33E-02	7.17E-03
sp900763885	UMGS1696_sp900763885.GCF_900763885_1	OK_sd	0.20	3.27E-02	7.02E-03
sp900552015	ER4_sp900552015.GCA_900552015_1	OK_sd	0.20	3.38E-02	7.33E-03
sp900762525	Bacteroides_sp900762525.GCA_900762525_1	OK_nc	0.20	3.37E-02	7.30E-03
sp900757905	UMGS882_sp900757905.GCF_900757905_1	OK_sd	0.20	3.30E-02	7.10E-03
sp900754535	UCG-010_sp900754535.GCF_900754535_1	OK_nc	0.19	3.46E-02	7.55E-03
sp900765975	Schaedlerella_sp900765975.GCF_900765975_1	OK_nc	0.19	3.60E-02	7.96E-03
sp900556635	CAG-170_sp900556635.GCA_900556635_1	OK_sd	0.19	3.60E-02	7.96E-03
sp3478995	Lawsonibacter_sp003478995.GCF_003478995_1	OK_nc	0.19	3.60E-02	7.96E-03
sp900545925	CAG-170_sp900545925.GCA_900545925_1	OK_sd	0.19	3.69E-02	8.21E-03

Custom ID	GTDB + Genome Assembly Species	Deconfounding status	Ds	p value	q value
sp165065	Clostridium.GCF_000165065_1	OK_nc	0.19	3.61E-02	7.99E-03
sp900549645	UBA1777_sp900549645.GCA_900549645_1	OK_sd	0.19	3.60E-02	7.94E-03
sp4560375	UBA9475_sp004560375.GCA_004560375_1	OK_sd	0.19	3.69E-02	8.24E-03
sp2949675	Escherichia_dysenteriae.GCF_002949675_1	OK_nc	0.19	3.83E-02	8.63E-03
sp242435	Dialister_succinatiphilus.GCF_000242435_1	OK_sd	0.19	3.74E-02	8.40E-03
sp379965	Prevotella_nanceiensis.GCF_000379965_1	OK_nc	0.19	3.69E-02	8.24E-03
sp3150195	CAG-533_sp003150195.GCA_003150195_1	OK_sd	0.19	3.83E-02	8.61E-03
sp752675	Olsenella.GCF_000752675_2	OK_nc	0.19	2.99E-02	6.03E-03
sp14287675	Pseudoflavonifractor_sp014287675.GCF_014287675_1	OK_sd	0.19	3.92E-02	8.92E-03
sp340375	Eggerthia_catenaformis.GCF_000340375_1	OK_nc	0.19	3.94E-02	9.00E-03
sp900543085	UBA5446_sp900543085.GCA_900543085_1	OK_nc	0.19	3.92E-02	8.90E-03
sp2298695	CAG-170_sp002298695.GCA_002298695_1	OK_sd	0.19	3.92E-02	8.88E-03
sp900554625	CAG-110_sp900554625.GCA_900554625_1	OK_sd	0.19	3.85E-02	8.71E-03
sp900552545	Duodenibacillus_sp900552545.GCA_900552545_1	OK_nc	0.19	3.94E-02	8.99E-03
sp900637655	Prevotella_oris.GCF_900637655_1	OK_nc	0.19	4.04E-02	9.37E-03
sp900291465	CAG-462_sp900291465.GCF_900291465_1	OK_nc	0.19	3.99E-02	9.17E-03
sp3697165	Escherichia_coli.GCF_003697165_2	OK_nc	0.19	4.17E-02	9.78E-03
sp434235	Alistipes_sp000434235.GCA_000434235_1	OK_sd	0.19	4.13E-02	9.63E-03
sp434895	Phascolarctobacterium.GCA_000434895_1	OK_nc	0.19	4.13E-02	9.63E-03
sp900757485	HGM13222_sp900757485.GCF_900757485_1	OK_sd	0.19	4.06E-02	9.43E-03
sp4558525	Phil1_sp004558525.GCA_004558525_1	OK_nc	0.19	4.03E-02	9.32E-03
sp3150615	UBA1820_sp003150615.GCA_003150615_1	OK_nc	0.19	4.24E-02	9.99E-03
sp900541415	Odoribacter_sp900541415.GCA_900541415_1	OK_nc	0.19	4.29E-02	1.01E-02
sp4558825	SFDB01_sp004558825.GCA_004558825_1	OK_sd	0.19	4.00E-02	9.20E-03
sp432435	CAG-180_sp000432435.GCA_000432435_1	OK_nc	0.19	4.35E-02	1.03E-02
sp1553605	Desulfovibrio_fairfieldensis.GCF_001553605_1	OK_nc	0.19	4.39E-02	1.05E-02
sp902781655	CAG-791_sp902781655.GCA_902781655_1	OK_sd	0.19	4.36E-02	1.04E-02
sp900113155	Enterocloster_clostridioformis.GCF_900113155_1	OK_sd	0.19	4.41E-02	1.06E-02
sp55555000903215	Escherichia_phage_TL-2011b.GCF_000903215_1	OK_nc	0.19	4.50E-02	1.09E-02
sp434195	CAG-245_sp000434195.GCA_000434195_1	OK_sd	0.19	3.73E-02	8.35E-03
sp4554205	UBA9502_sp004554205.GCA_004554205_1	OK_sd	0.19	4.47E-02	1.07E-02
sp900551245	Victivallis_sp900551245.GCA_900551245_1	OK_nc	0.18	4.59E-02	1.12E-02
sp759755	Citrobacter_werkmanii.GCF_000759755_1	OK_nc	0.18	4.80E-02	1.18E-02
sp2437245	CAG-180_sp002437245.GCA_002437245_1	OK_sd	0.18	4.62E-02	1.13E-02
sp437015	CAG-390_sp000437015.GCA_000437015_1	OK_sd	0.18	4.71E-02	1.16E-02
sp4558115	Dysosmobacter_sp004558115.GCA_004558115_1	OK_sd	0.18	4.86E-02	1.21E-02
sp12843875	Desulfovibrio_sp900556755.GCF_012843875_1	OK_sd	0.18	4.83E-02	1.19E-02
sp55555000866205	Escherichia_phage_phiV10.GCF_000866205_1	OK_nc	0.18	4.58E-02	1.11E-02
sp900767715	HGM11372_sp900767715.GCF_900767715_1	OK_nc	0.18	4.24E-02	9.98E-03
sp900545155	Akkermansia_sp900545155.GCA_900545155_1	OK_nc	0.18	4.36E-02	1.04E-02
sp900753295	CAG-390_sp900753295.GCF_900753295_1	OK_nc	0.18	4.95E-02	1.24E-02
sp900553105	CAG-521_sp900553105.GCA_900553105_1	OK_nc	0.18	2.81E-02	5.36E-03
sp900554485	UBA10281_sp900554485.GCA_900554485_1	OK_nc	0.17	2.54E-02	4.60E-03

Custom ID	GTDB + Genome Assembly Species	Deconfounding status	Ds	p value	q value
sp431835	Succinivibrio_sp000431835.GCA_000431835_1	OK_nc	-0.15	2.79E-02	5.29E-03
sp438255	CAG-269_sp000438255.GCA_000438255_1	OK_nc	-0.18	3.61E-02	8.01E-03
sp900544435	Faecalibacillus_sp900544435.GCA_900544435_1	OK_nc	-0.18	5.00E-02	1.25E-02
sp55555000182805	Sordaria_macrospora_k-hell.GCF_000182805_2	OK_nc	-0.18	4.83E-02	1.20E-02
sp785515	Streptococcus_salivarius.GCF_000785515_1	OK_sd	-0.18	4.95E-02	1.24E-02
sp1405555	Fusicatenibacter_saccharivorans.GCF_001405555_1	OK_nc	-0.18	4.89E-02	1.22E-02
sp902362375	Bacteroides_sp902362375.GCF_902362375_1	OK_sd	-0.18	4.84E-02	1.20E-02
sp1543345	Turicibacter_sp001543345.GCF_001543345_1	OK_nc	-0.18	4.80E-02	1.18E-02
sp14287815	Clostridium_sp001916075.GCF_014287815_1	OK_nc	-0.18	4.50E-02	1.09E-02
sp154425	Coprococcus_eutactus.GCF_000154425_1	OK_nc	-0.18	4.60E-02	1.12E-02
sp900544795	Phascolarctobacterium_sp900544795.GCA_900544795_1	OK_nc	-0.19	4.50E-02	1.09E-02
sp900550975	Faecalimonas_sp900550975.GCA_900550975_1	OK_nc	-0.19	4.54E-02	1.10E-02
sp20605	Agathobacter_rectalis.GCF_000020605_1	OK_nc	-0.19	4.41E-02	1.06E-02
sp146185	Lachnospira_eligens.GCF_000146185_1	OK_nc	-0.19	4.36E-02	1.04E-02
sp225345	Roseburia_hominis.GCF_000225345_1	OK_nc	-0.19	4.34E-02	1.03E-02
sp900769635	UMGS2068_sp900769635.GCF_900769635_1	OK_nc	-0.19	4.13E-02	9.67E-03
sp238795	Haemophilus.GCF_000238795_1	OK_nc	-0.19	3.99E-02	9.17E-03
sp435595	CAG-882_sp000435595.GCA_000435595_1	OK_sd	-0.19	4.01E-02	9.26E-03
sp4562005	Roseburia_sp004562005.GCA_004562005_1	OK_nc	-0.19	3.51E-02	7.67E-03
sp1461035	Acetivibrio.GCF_001461035_1	OK_nc	-0.19	3.45E-02	7.50E-03
sp900555625	UBA9502_sp900555625.GCA_900555625_1	OK_nc	-0.20	3.35E-02	7.25E-03
sp3478445	TF01-11_sp003524945.GCF_003478445_1	OK_nc	-0.20	3.27E-02	7.02E-03
sp900549355	Collinsella_sp900549355.GCA_900549355_1	OK_nc	-0.20	3.15E-02	6.55E-03
sp900770165	HGM12814_sp900770165.GCF_900770165_1	OK_nc	-0.20	3.09E-02	6.39E-03
sp900549235	Enterocloster_sp900549235.GCA_900549235_1	OK_nc	-0.20	3.00E-02	6.07E-03
sp3473545	Enterocloster_sp000431375.GCF_003473545_1	OK_nc	-0.20	3.03E-02	6.18E-03
sp900546435	AM51-8_sp900546435.GCA_900546435_1	OK_nc	-0.20	2.96E-02	5.90E-03
sp900552445	Veillonella_sp900552445.GCA_900552445_1	OK_nc	-0.20	2.85E-02	5.59E-03
sp2222595	Blautia_hansenii.GCF_002222595_2	OK_nc	-0.20	2.84E-02	5.45E-03
sp173975	Anaerobutyricum_hallii.GCF_000173975_1	OK_nc	-0.20	2.84E-02	5.43E-03
sp3460505	Eubacterium.GCF_003460505_1	OK_nc	-0.20	2.84E-02	5.46E-03
sp332875	Anaerostipes_hadrus.GCF_000332875_2	OK_nc	-0.20	2.78E-02	5.25E-03
sp900549995	UMGS1251_sp900549995.GCA_900549995_1	OK_nc	-0.20	2.67E-02	4.96E-03
sp4346095	Faecalimonas_umbilicata.GCF_004346095_1	OK_nc	-0.21	2.62E-02	4.82E-03
sp1182045	Mesosutterella_massiliensis.GCF_001182045_1	OK_nc	-0.21	2.41E-02	4.30E-03
sp900550845	Agathobacter_sp900550845.GCA_900550845_1	OK_nc	-0.21	2.53E-02	4.57E-03
sp1458595	Clostridium_neonatale.GCF_001458595_1	OK_nc	-0.21	2.05E-02	3.46E-03
sp900552665	Roseburia_sp900552665.GCA_900552665_1	OK_nc	-0.21	2.08E-02	3.59E-03
sp3481985	UBA9502_sp003506385.GCF_003481985_1	OK_nc	-0.21	2.08E-02	3.57E-03
sp3464165	Lachnospira_sp003451515.GCF_003464165_1	OK_nc	-0.21	2.08E-02	3.54E-03
sp723465	Ruminococcus.GCF_000723465_1	OK_nc	-0.21	2.08E-02	3.56E-03
sp900542495	Roseburia_sp900542495.GCA_900542495_1	OK_nc	-0.21	1.96E-02	3.27E-03
sp3477605	Eubacterium.GCF_003477605_1	OK_nc	-0.21	1.99E-02	3.34E-03

Custom ID	GTDB + Genome Assembly Species	Deconfounding status	Ds	p value	q value
sp900546625	Agathobacter_sp900546625.GCA_900546625_1	OK_nc	-0.21	1.90E-02	3.17E-03
sp900555025	Blautia_sp900555025.GCA_900555025_1	OK_nc	-0.21	1.90E-02	3.15E-03
sp6742065	Clostridium_butyricum.GCF_006742065_1	OK_nc	-0.22	1.77E-02	2.82E-03
sp156015	Butyrivibrio.GCF_000156015_1	OK_nc	-0.22	1.72E-02	2.70E-03
sp9680455	Lachnospira_eligens.GCF_009680455_1	OK_nc	-0.22	1.72E-02	2.65E-03
sp14334015	Bacteroides_sp003463205.GCF_014334015_1	OK_sd	-0.22	1.71E-02	2.61E-03
sp900757025	HGM11808_sp900757025.GCF_900757025_1	OK_nc	-0.22	1.65E-02	2.50E-03
sp433635	Ruminococcus.GCA_000433635_1	OK_nc	-0.22	1.65E-02	2.48E-03
sp900549895	Agathobacter_sp900549895.GCA_900549895_1	OK_nc	-0.22	1.64E-02	2.46E-03
sp900753905	Blautia_sp900753905.GCA_900753905_1	OK_nc	-0.22	1.60E-02	2.35E-03
sp900543325	Clostridium_sp900543325.GCA_900543325_1	OK_nc	-0.22	1.54E-02	2.20E-03
sp900317585	Agathobacter_sp900317585.GCA_900317585_1	OK_nc	-0.22	1.50E-02	2.11E-03
sp900759665	TF01-11_sp900759665.GCF_900759665_1	OK_nc	-0.22	1.44E-02	1.99E-03
sp3458705	Lachnospira_sp003537285.GCF_003458705_1	OK_nc	-0.22	1.45E-02	2.01E-03
sp900550545	Agathobacter_sp900550545.GCA_900550545_1	OK_nc	-0.22	1.48E-02	2.07E-03
sp902363515	Blautia_sp000432195.GCF_902363515_1	OK_nc	-0.23	1.31E-02	1.77E-03
sp3402575	Mesosutterella_multiformis.GCF_003402575_1	OK_nc	-0.23	1.30E-02	1.76E-03
sp3480255	Faecalibacillus_sp003480255.GCF_003480255_1	OK_nc	-0.23	1.26E-02	1.68E-03
sp900539375	Clostridium_sp900539375.GCA_900539375_1	OK_nc	-0.23	1.17E-02	1.56E-03
sp3293635	Faecalibacterium_prausnitzii.GCF_003293635_1	OK_nc	-0.24	9.13E-03	1.11E-03
sp13302405	Blautia.GCF_013302405_1	OK_nc	-0.24	8.08E-03	9.36E-04
sp1406815	Agathobacter_faecis.GCF_001406815_1	OK_nc	-0.24	7.86E-03	9.01E-04
sp2474415	Agathobacter_sp002474415.GCA_002474415_1	OK_nc	-0.24	7.02E-03	7.79E-04
sp1940225	14-2_sp001940225.GCF_001940225_1	OK_nc	-0.25	6.72E-03	7.35E-04
sp3482105	Coprococcus_sp000433075.GCF_003482105_1	OK_nc	-0.25	6.40E-03	6.83E-04
sp902786365	CAG-603_sp902786365.GCA_902786365_1	OK_nc	-0.25	6.24E-03	6.63E-04
sp900545305	CAG-274_sp900545305.GCA_900545305_1	OK_nc	-0.25	6.24E-03	6.57E-04
sp900766915	HGM12587_sp900766915.GCF_900766915_1	OK_nc	-0.25	5.89E-03	5.92E-04
sp8661915	Blautia.GCA_008661915_1	OK_nc	-0.25	5.87E-03	5.87E-04
sp900547255	Lachnospira_sp900547255.GCA_900547255_1	OK_nc	-0.25	5.38E-03	5.02E-04
sp437215	CAG-269_sp000437215.GCA_000437215_1	OK_sd	-0.25	2.61E-03	1.99E-04
sp902793335	CAG-590_sp902793335.GCA_902793335_1	OK_nc	-0.25	5.22E-03	4.79E-04
sp902363825	CAG-45_sp000438375.GCF_902363825_1	OK_nc	-0.25	5.12E-03	4.59E-04
sp900770535	UBA9502_sp900770535.GCF_900770535_1	OK_nc	-0.26	4.96E-03	4.39E-04
sp3478335	TF01-11_sp003529475.GCF_003478335_1	OK_nc	-0.26	4.16E-03	3.51E-04
sp900557055	Agathobacter_sp900557055.GCA_900557055_1	OK_nc	-0.26	3.96E-03	3.32E-04
sp3470905	Roseburia_sp003470905.GCF_003470905_1	OK_nc	-0.26	3.54E-03	2.84E-04
sp900543865	Butyrivibrio.GCA_900543865_1	OK_nc	-0.27	3.32E-03	2.63E-04
sp436475	Lachnospira_sp000436475.GCA_000436475_1	OK_nc	-0.27	3.32E-03	2.63E-04
sp437755	CAG-303_sp000437755.GCA_000437755_1	OK_nc	-0.27	3.17E-03	2.47E-04
sp902363675	Agathobacter_sp000434275.GCF_902363675_1	OK_nc	-0.27	2.13E-03	1.56E-04
sp12519695	14-2_sp012519695.GCA_012519695_1	OK_nc	-0.28	2.03E-03	1.46E-04
sp3478505	UBA9502_sp003478505.GCF_003478505_1	OK_nc	-0.28	1.36E-03	9.19E-05

Custom ID	GTDB + Genome Assembly Species	Deconfounding status	Ds	p value	q value
sp3486385	CAG-882_sp003486385.GCA_003486385_1	OK_sd	-0.29	1.24E-03	8.05E-05
sp900550235	Faecalimonas_sp900550235.GCA_900550235_1	OK_nc	-0.29	1.12E-03	7.08E-05
sp902362455	CAG-603_sp900066105.GCF_902362455_1	OK_nc	-0.29	1.16E-03	7.41E-05
sp9695765	VUNI01_sp009695765.GCF_009695765_1	OK_nc	-0.29	9.68E-04	5.95E-05
sp1940165	Roseburia_sp001940165.GCF_001940165_1	OK_nc	-0.29	9.09E-04	5.40E-05
sp900552085	Agathobacter_sp900552085.GCA_900552085_1	OK_nc	-0.29	8.59E-04	5.06E-05
sp437735	Lachnospira_sp000437735.GCA_000437735_1	OK_nc	-0.30	7.12E-04	3.97E-05
sp14384965	TF01-11_sp003149875.GCA_014384965_1	OK_nc	-0.30	5.26E-04	2.82E-05
sp1517625	Enterocloster_sp001517625.GCF_001517625_2	OK_nc	-0.31	4.48E-04	2.29E-05
sp900106845	Romboutsia_timonensis.GCF_900106845_1	OK_nc	-0.31	4.26E-04	2.15E-05
sp900015215	Romboutsia_ilealis.GCF_900015215_1	OK_nc	-0.31	3.99E-04	1.98E-05
sp14287635	1XD42-69_sp014287635.GCF_014287635_1	OK_nc	-0.31	3.70E-04	1.81E-05
sp3481585	TF01-11_sp001916135.GCF_003481585_1	OK_nc	-0.31	3.17E-04	1.52E-05
sp900537995	Roseburia_intestinalis.GCF_900537995_1	OK_nc	-0.32	2.87E-04	1.32E-05
sp900552795	Lachnospira_sp900552795.GCA_900552795_1	OK_nc	-0.32	2.28E-04	9.86E-06
sp900551945	Lachnospira_sp900551945.GCA_900551945_1	OK_nc	-0.32	1.96E-04	8.28E-06
sp900547695	Agathobacter_sp900547695.GCA_900547695_1	OK_nc	-0.32	1.96E-04	8.17E-06
sp155855	Bacteroides.GCF_000155855_1	OK_nc	-0.35	5.87E-05	2.06E-06
sp900554965	Anaerobutyricum_sp900554965.GCA_900554965_1	OK_nc	-0.35	4.55E-05	1.54E-06
sp3460745	CAG-41_sp900066215.GCF_003460745_1	OK_nc	-0.36	2.21E-05	6.80E-07
sp160755	Proteus_mirabilis.GCF_000160755_1	OK_nc	-0.36	5.03E-06	1.28E-07
sp2435585	Lachnospira_sp002435585.GCA_002435585_1	OK_nc	-0.36	2.17E-05	6.57E-07
sp900545725	Lachnospira_sp900545725.GCA_900545725_1	OK_nc	-0.37	1.67E-05	4.70E-07
sp3483745	Roseburia_sp003483745.GCA_003483745_1	OK_nc	-0.37	1.33E-05	3.67E-07
sp1414325	TF01-11_sp001414325.GCF_001414325_1	OK_nc	-0.40	2.22E-06	5.20E-08
sp436755	TF01-11_sp000436755.GCA_000436755_1	OK_nc	-0.45	3.29E-08	5.66E-10
sp436535	Lachnospira_sp000436535.GCA_000436535_1	OK_nc	-0.46	1.96E-08	3.17E-10

Supplementary Table S6. Deconfounding analysis and pairwise comparisons of species KO relative abundances in CRC versus healthy controls.

KO_Id	Annotation	Deconfounding Status	Ds	p value	q value
K07233	NA	OK_nc	0.812	2.03E-25	4.70E-29
K05011	CLCN2	OK_nc	0.49	2.99E-08	1.38E-11
K01035	atoA	OK_nc	0.43	4.60E-06	3.20E-09
K01034	atoD	OK_nc	0.42	6.06E-06	6.99E-09
K19268	glmE	OK_nc	0.4	2.19E-05	3.56E-08
K01846	glmS	OK_nc	0.384	5.81E-05	1.21E-07
K18012	kdd	OK_nc	0.376	6.89E-05	2.23E-07
K01969	E6-4-1-4B	OK_nc	0.373	8.15E-05	2.83E-07

KO_Id	Annotation	Deconfounding Status	Ds	p value	q value
K01844	kamD	OK_nc	0.372	8.15E-05	3.02E-07
K04835	mal	OK_nc	0.371	8.28E-05	3.26E-07
K12527	ygfK	OK_nc	0.369	8.60E-05	3.73E-07
K00132	E1-2-1-10	OK_nc	0.369	8.60E-05	3.79E-07
K01101	E3-1-3-41	OK_nc	0.366	8.95E-05	4.52E-07
K18014	kal	OK_nc	0.365	8.95E-05	5.01E-07
K00282	gcvPA	OK_nc	0.364	8.95E-05	5.42E-07
K18011	kamE	OK_nc	0.363	8.95E-05	5.56E-07
K10960	chlP	OK_nc	0.363	8.95E-05	5.60E-07
K14534	abfD	OK_nc	0.359	0.000108552	7.62E-07
K02453	gspD	OK_nc	0.358	0.000108552	7.80E-07
K00989	rph	OK_nc	0.356	0.000117635	9.48E-07
K00283	gcvPB	OK_nc	0.356	0.000117635	9.55E-07
K02990	RP-S6	OK_nc	0.355	0.000117635	1.00E-06
K00863	DAK	OK_nc	0.355	0.000117635	9.85E-07
K06015	NA	OK_nc	0.344	0.000211292	2.12E-06
K10672	NA	OK_nc	0.343	0.000211292	2.30E-06
K10671	NA	OK_nc	0.343	0.000211292	2.30E-06
K03605	hyaD	OK_nc	0.343	0.000211292	2.27E-06
K01464	DPYS	OK_nc	0.343	0.000211292	2.22E-06
K03522	fixB	OK_sd	0.341	0.000231944	2.63E-06
K18013	kce	OK_nc	0.336	0.000288663	3.54E-06
K18979	queG	OK_nc	0.335	0.000310677	3.96E-06
K02935	RP-L7	OK_nc	0.334	0.000320254	4.17E-06
K03833	selB	OK_nc	0.333	0.000332898	4.55E-06
K10670	NA	OK_nc	0.331	0.00035341	5.08E-06
K06178	rluB	OK_nc	0.331	0.00035341	5.04E-06
K01667	tnaA	OK_nc	0.323	0.000500626	8.58E-06
K09883	cobT	OK_nc	0.32	0.000575032	1.04E-05
K12529	ygfM	OK_nc	0.319	0.000583985	1.07E-05
K02952	RP-S13	OK_nc	0.317	0.000644121	1.24E-05
K00060	tdh	OK_nc	0.313	0.000755765	1.56E-05
K16329	psuG	OK_nc	0.311	0.00085092	1.81E-05
K12267	NA	OK_nc	0.31	0.000907077	2.00E-05
K00325	pntB	OK_nc	0.31	0.000891965	1.94E-05
K03672	trxC	OK_nc	0.308	0.000947908	2.14E-05
K07770	cssR	OK_nc	0.307	0.000990855	2.27E-05
K01478	arcA	OK_sd	0.307	0.001007972	2.34E-05
K01483	allA	OK_nc	0.305	0.001093886	2.59E-05
K01042	selA	OK_nc	0.305	0.001097999	2.62E-05
K02613	paaE	OK_nc	0.303	0.001201088	2.98E-05
K03183	ubiE	OK_nc	0.302	0.00123059	3.08E-05
K05979	comB	OK_nc	0.301	0.001309134	3.34E-05

KO_Id	Annotation	Deconfounding Status	Ds	p value	q value
K00390	cysH	OK_nc	0.301	0.001320508	3.43E-05
K02967	RP-S2	OK_nc	0.3	0.001353121	3.54E-05
K02848	waaP	OK_nc	0.299	0.00138367	3.69E-05
K16203	dppA1	OK_nc	0.298	0.001426275	3.97E-05
K06608	iolR	OK_nc	0.298	0.001462004	4.10E-05
K11810	SLC16A12	OK_nc	0.297	0.001491877	4.26E-05
K01159	ruvC	OK_nc	0.297	0.001491877	4.32E-05
K13870	SLC7A12_13	OK_nc	0.296	0.001568153	4.60E-05
K07644	cusS	OK_nc	0.295	0.001605778	4.77E-05
K04019	eutA	OK_nc	0.295	0.001623204	4.93E-05
K02916	RP-L35	OK_nc	0.295	0.001605778	4.80E-05
K04032	eutT	OK_nc	0.294	0.001695576	5.19E-05
K02437	gcvH	OK_nc	0.294	0.001695576	5.23E-05
K09975	NA	OK_nc	0.293	0.001714566	5.36E-05
K00087	ygeS	OK_nc	0.293	0.001714566	5.36E-05
K16926	htsT	OK_nc	0.292	0.001826551	5.80E-05
K00324	pntA	OK_nc	0.291	0.001919308	6.23E-05
K07084	yuiF	OK_nc	0.29	0.001928197	6.39E-05
K00639	kbl	OK_nc	0.29	0.001928197	6.39E-05
K14491	ARR-B	OK_nc	0.289	0.002067571	6.94E-05
K09960	NA	OK_nc	0.289	0.002067571	6.95E-05
K02919	RP-L36	OK_nc	0.287	0.002258622	7.75E-05
K00134	GAPDH	OK_nc	0.286	0.002375164	8.25E-05
K03735	eutB	OK_nc	0.285	0.002465599	8.74E-05
K00803	AGPS	OK_nc	0.285	0.002420208	8.47E-05
K00068	srlD	OK_nc	0.285	0.002465599	8.69E-05
K01479	hutG	OK_nc	0.284	0.002480848	8.85E-05
K00674	dapD	OK_nc	0.283	0.002709651	9.79E-05
K16261	YAT	OK_nc	0.282	0.002726411	0.000101072
K02518	infA	OK_nc	0.282	0.002727195	0.000102996
K00355	NQO1	OK_nc	0.282	0.002726349	0.000100438
K18856	vanC	OK_nc	0.281	0.002780704	0.00010695
K09961	NA	OK_nc	0.281	0.002780064	0.000106281
K02879	RP-L17	OK_nc	0.281	0.002834217	0.000109665
K01697	CBS	OK_nc	0.281	0.00277952	0.000105617
K00076	hdhA	OK_nc	0.278	0.003275183	0.000129763
K00009	mtlD	OK_nc	0.278	0.003276397	0.00013057
K03736	eutC	OK_nc	0.277	0.003362891	0.000136354
K02939	RP-L9	OK_nc	0.277	0.003364525	0.0001372
K01040	gctB	OK_nc	0.277	0.003277715	0.000131382
K00823	puuE	OK_nc	0.277	0.003340624	0.000134678
K18189	TACO1	OK_nc	0.275	0.003582351	0.000147743
K13599	ntrX	OK_nc	0.274	0.003725668	0.000157106

KO_Id	Annotation	Deconfounding Status	Ds	p value	q value
K12516	bigA	OK_nc	0.274	0.003770498	0.000161006
K06928	NTPCR	OK_nc	0.274	0.003721116	0.00015519
K04112	bcrC	OK_nc	0.274	0.003723339	0.000156146
K01482	DDAH	OK_nc	0.274	0.003770498	0.000162491
K07748	NSDHL	OK_nc	0.273	0.003861104	0.000168637
K03179	ubiA	OK_nc	0.273	0.003891283	0.000172205
K01039	gctA	OK_nc	0.273	0.003819809	0.000165501
K13866	SLC7A4	OK_nc	0.271	0.004205065	0.000190962
K02954	RP-S14	OK_nc	0.271	0.004175668	0.00018866
K04078	groES	OK_nc	0.27	0.004290594	0.000196834
K15256	cmoA	OK_nc	0.269	0.004455536	0.000210356
K12960	mtaD	OK_nc	0.269	0.004455536	0.000211628
K08155	SLC18A1_2	OK_nc	0.269	0.004455536	0.000210987
K03098	APOD	OK_nc	0.269	0.004460699	0.000212906
K03577	acrR	OK_nc	0.268	0.004624859	0.000226099
K02519	infB	OK_nc	0.268	0.004619074	0.000224746
K01916	nadE	OK_nc	0.268	0.004630745	0.00022746
K01250	rihA	OK_nc	0.268	0.004498318	0.000216786
K16353	SDK	OK_nc	0.267	0.00438739	0.000204971
K03979	obgE	OK_nc	0.267	0.004636732	0.000228829
K07112	NA	OK_nc	0.266	0.004847898	0.000241496
K05396	dcyD	OK_nc	0.266	0.004912709	0.000245863
K03666	hfq	OK_nc	0.266	0.004992096	0.000253306
K02455	gspF	OK_nc	0.266	0.004985194	0.000251801
K01630	garL	OK_nc	0.266	0.004847898	0.000241496
K00927	PGK	OK_nc	0.266	0.004948788	0.000248815
K00381	cysI	OK_nc	0.265	0.00516454	0.000265646
K18285	mqnE	OK_nc	0.264	0.005187464	0.000269765
K17992	NA	OK_nc	0.264	0.005336073	0.000281887
K02986	RP-S4	OK_nc	0.264	0.005187464	0.000270431
K00681	ggt	OK_nc	0.264	0.005187464	0.000270431
K10124	bglH	OK_nc	0.263	0.005501749	0.000291914
K13252	NA	OK_nc	0.262	0.005680608	0.000307984
K08682	acpH	OK_nc	0.262	0.005689812	0.000309802
K02864	RP-L10	OK_nc	0.262	0.005680608	0.000306177
K04076	lonB	OK_nc	0.261	0.005913318	0.000324712
K01777	prdF	OK_nc	0.261	0.005800544	0.000317175
K08221	yitG	OK_nc	0.26	0.006028179	0.000332416
K03800	lplA	OK_nc	0.26	0.006165845	0.000344293
K03761	kgtP	OK_nc	0.26	0.006073671	0.000336332
K03742	pncC	OK_nc	0.26	0.006119558	0.000340291
K06077	NA	OK_nc	0.259	0.006370118	0.000358651
K03040	rpoA	OK_nc	0.259	0.006370118	0.000358651

KO_Id	Annotation	Deconfounding Status	Ds	p value	q value
K01619	deoC	OK_nc	0.259	0.006493553	0.000367105
K16291	erfK	OK_nc	0.258	0.006527607	0.000371402
K02838	frr	OK_nc	0.258	0.006589034	0.000380137
K01118	NA	OK_nc	0.258	0.00657729	0.000377935
K00556	trmH	OK_sd	0.258	0.006527607	0.000373568
K03382	atzB	OK_nc	0.257	0.006807343	0.000395886
K01058	pldA	OK_nc	0.257	0.006812427	0.000398185
K17879	NUDT7	OK_nc	0.256	0.007140376	0.000421871
K06878	NA	OK_nc	0.256	0.007085998	0.000417017
K03758	arcD	OK_nc	0.256	0.007153615	0.000424311
K06948	yqeH	OK_nc	0.255	0.007460744	0.000449442
K05939	aas	OK_nc	0.255	0.007390075	0.000441761
K00176	korD	OK_nc	0.255	0.007460744	0.000449442
K09131	NA	OK_nc	0.254	0.007722684	0.000475958
K16090	fiu	OK_nc	0.253	0.008019813	0.000501062
K09123	lhpI	OK_nc	0.253	0.008000599	0.000496793
K02988	RP-S5	OK_nc	0.253	0.007826874	0.000484193
K15428	DUG1	OK_nc	0.252	0.008141537	0.000523045
K11784	mqnC	OK_nc	0.252	0.008019813	0.000501703
K09769	ymdB	OK_nc	0.252	0.008058134	0.000509701
K03670	NA	OK_nc	0.252	0.0080255	0.000503918
K02871	RP-L13	OK_nc	0.252	0.008107806	0.000518479
K02600	nusA	OK_nc	0.252	0.008041823	0.000506806
K01893	NARS	OK_nc	0.252	0.008107806	0.000518479
K01644	citE	OK_nc	0.252	0.008141537	0.000524409
K18119	sucD	OK_nc	0.251	0.008218335	0.000535068
K16137	nemR	OK_nc	0.251	0.008175759	0.000530401
K02106	atoE	OK_nc	0.251	0.008304155	0.000542579
K16138	uidR	OK_nc	0.25	0.008554373	0.000562892
K13479	ygeT	OK_nc	0.25	0.008597472	0.00056772
K09807	NA	OK_nc	0.25	0.008554373	0.000561335
K03667	hslU	OK_nc	0.25	0.008664786	0.000574173
K10001	gltI	OK_nc	0.249	0.00906374	0.000607472
K09972	aapP	OK_nc	0.249	0.00906374	0.000605755
K02931	RP-L5	OK_nc	0.249	0.009033097	0.000600672
K17865	croR	OK_nc	0.248	0.00906374	0.000617807
K08093	hxlA	OK_nc	0.248	0.009213608	0.00063897
K07488	NA	OK_nc	0.248	0.009084519	0.000623035
K07469	NA	OK_nc	0.248	0.009213608	0.000642562
K01646	citD	OK_nc	0.248	0.009213608	0.000642562
K01451	hipO	OK_nc	0.248	0.00906374	0.000617807
K00253	IVD	OK_nc	0.248	0.009151102	0.000631842
K03289	nupG	OK_nc	0.247	0.009329349	0.000657118

KO_Id	Annotation	Deconfounding Status	Ds	p value	q value
K03043	rpoB	OK_nc	0.247	0.009509081	0.000671981
K12811	DDX46	OK_nc	0.246	0.009682957	0.000690905
K12137	NA	OK_nc	0.246	0.009682957	0.000690999
K02530	lacR	OK_nc	0.246	0.009979603	0.00071448
K00962	pnp	OK_nc	0.246	0.009682957	0.000687156
K11939	rhtA	OK_nc	0.245	0.010058659	0.000722471
K12950	NA	OK_nc	0.244	0.010476722	0.000757353
K07719	glnL	OK_nc	0.244	0.010501405	0.000761571
K02890	RP-L22	OK_nc	0.244	0.010580141	0.000772184
K02888	RP-L21	OK_nc	0.244	0.010580141	0.000772184
K01753	dsdA	OK_nc	0.244	0.01068923	0.000785099
K10942	flrB	OK_nc	0.243	0.010945542	0.000811532
K02836	prfB	OK_nc	0.243	0.010964049	0.000820525
K02510	hpaI	OK_nc	0.243	0.010964049	0.000816017
K12975	eptB	OK_nc	0.242	0.011255055	0.000852735
K10545	xylG	OK_nc	0.242	0.011112217	0.000834189
K04021	eutE	OK_nc	0.242	0.011369051	0.000869274
K02221	yggT	OK_nc	0.242	0.011227725	0.000848063
K01026	pct	OK_nc	0.242	0.011344638	0.00086215
K00951	relA	OK_nc	0.242	0.011369051	0.000866894
K16169	pbuX	OK_nc	0.241	0.011703936	0.000905726
K15519	dck	OK_sd	0.241	0.011753358	0.000915448
K11179	tusE	OK_nc	0.241	0.011491145	0.000881272
K02948	RP-S11	OK_nc	0.241	0.011519471	0.000886113
K11743	mdtJ	OK_nc	0.24	0.011940493	0.000946159
K09913	ppnP	OK_nc	0.24	0.011753358	0.000922981
K07305	NA	OK_nc	0.24	0.011753358	0.000923167
K03820	NA	OK_nc	0.24	0.011753358	0.000920699
K00605	gcvT	OK_nc	0.24	0.011815792	0.000930808
K00574	NA	OK_nc	0.24	0.011940493	0.000946165
K19237	kgp	OK_nc	0.239	0.012055018	0.00097497
K13895	yejE	OK_nc	0.239	0.012183185	0.000993624
K13049	PM20D1	OK_nc	0.239	0.012055018	0.000977585
K09914	NA	OK_nc	0.239	0.012055018	0.000972282
K09128	acnX2	OK_nc	0.239	0.012086022	0.000982899
K02451	gspB	OK_nc	0.239	0.012026704	0.000964143
K07480	NA	OK_nc	0.238	0.012308743	0.001020975
K03687	GRPE	OK_nc	0.238	0.012308743	0.001015461
K03046	rpoC	OK_nc	0.238	0.012341234	0.00102653
K02863	RP-L1	OK_nc	0.238	0.012373805	0.001032106
K02454	gspE	OK_nc	0.238	0.012308743	0.001015461
K02452	gspC	OK_nc	0.238	0.012308743	0.001020951
K02362	entD	OK_nc	0.238	0.012308743	0.001020894

KO_Id	Annotation	Deconfounding Status	Ds	p value	q value
K00177	korC	OK_nc	0.238	0.01240653	0.00103771
K14007	SEC24	OK_nc	0.237	0.012950009	0.001113173
K06942	ychF	OK_nc	0.237	0.012950009	0.001113173
K06350	NA	OK_nc	0.237	0.012709023	0.001077735
K18478	NA	OK_nc	0.236	0.012950115	0.001119183
K03980	murJ	OK_nc	0.236	0.013233841	0.001162101
K03796	NA	OK_nc	0.236	0.013233083	0.001158968
K03076	secY	OK_nc	0.236	0.012950564	0.001125223
K01589	purK	OK_nc	0.236	0.013161918	0.001149686
K00800	aroA	OK_sd	0.236	0.013235216	0.001168354
K00058	serA	OK_nc	0.236	0.012950115	0.001119183
K09703	NA	OK_nc	0.235	0.013308102	0.001180955
K02887	RP-L20	OK_nc	0.235	0.013383976	0.001200092
K02457	gspH	OK_nc	0.235	0.013383976	0.00119678
K00879	fucK	OK_nc	0.235	0.013421145	0.001206535
K00140	mmsA	OK_nc	0.235	0.013383976	0.001193682
K17231	IYD	OK_nc	0.234	0.013935545	0.001259236
K04097	HPGDS	OK_nc	0.234	0.013799738	0.001243767
K06895	lysE	OK_nc	0.233	0.014778815	0.001342283
K11103	dctA	OK_nc	0.232	0.015088187	0.001415319
K03724	lhr	OK_nc	0.232	0.015030819	0.001378355
K03521	NA	OK_nc	0.232	0.015030819	0.001393032
K03078	ulaD	OK_nc	0.232	0.015088187	0.001419044
K02992	RP-S7	OK_nc	0.232	0.015030819	0.001393032
K02878	RP-L16	OK_nc	0.232	0.015088187	0.001415319
K02460	gspK	OK_nc	0.232	0.014938957	0.001363751
K00135	gabD	OK_nc	0.232	0.015072902	0.001400425
K09815	znuA	OK_nc	0.231	0.015405459	0.001476366
K07160	pxpA	OK_nc	0.231	0.015088187	0.00142282
K02881	RP-L18	OK_nc	0.231	0.015405459	0.001476366
K01608	gcl	OK_nc	0.231	0.015251269	0.001441733
K01596	E4-1-1-32	OK_nc	0.231	0.015254214	0.001445545
K00034	gdh	OK_nc	0.231	0.015378442	0.001460881
K13894	yejB	OK_nc	0.23	0.015405459	0.001491927
K11785	mqnD	OK_nc	0.23	0.015405459	0.001483144
K07821	torY	OK_nc	0.23	0.015494348	0.001507791
K05796	NA	OK_nc	0.23	0.015494348	0.001507791
K02884	RP-L19	OK_nc	0.23	0.015405459	0.001492002
K01628	fucA	OK_nc	0.23	0.015583968	0.001523734
K00123	fdoG	OK_nc	0.23	0.015405459	0.001492002
K16346	xanQ	OK_nc	0.229	0.016177862	0.001593047
K11782	mqnA	OK_nc	0.229	0.016082525	0.001579933
K07164	NA	OK_nc	0.229	0.016193077	0.001605801

KO_Id	Annotation	Deconfounding Status	Ds	p value	q value
K02994	RP-S8	OK_nc	0.229	0.016044681	0.001572498
K00333	nuoD	OK_nc	0.229	0.016193077	0.001605755
K18346	vanW	OK_nc	0.228	0.016592814	0.001656975
K16140	uidC	OK_nc	0.228	0.016592814	0.001655668
K11717	sufS	OK_nc	0.228	0.016583328	0.001648343
K02520	infC	OK_nc	0.228	0.016641059	0.001665648
K01251	E3-3-1-1	OK_nc	0.228	0.016651042	0.001674363
K00073	allD	OK_nc	0.228	0.016797449	0.001700761
K16370	pfkB	OK_nc	0.227	0.017072817	0.001736554
K13629	dsdX	OK_nc	0.227	0.017505193	0.001800812
K02956	RP-S15	OK_nc	0.227	0.017173069	0.001754709
K02357	tsf	OK_nc	0.227	0.017072817	0.001736554
K00954	E2-7-7-3A	OK_nc	0.227	0.017122844	0.00174561
K17550	PPP1R7	OK_nc	0.226	0.017948083	0.0018838
K13892	gsiA	OK_nc	0.226	0.017923179	0.001872185
K06971	NA	OK_nc	0.226	0.017740091	0.001829087
K02431	NA	OK_nc	0.226	0.017804744	0.001848129
K00888	PI4KA	OK_nc	0.226	0.017856771	0.001857667
K00257	NA	OK_nc	0.226	0.017923179	0.001877034
K13641	iclR	OK_nc	0.225	0.018314017	0.001946099
K12146	hyfR	OK_nc	0.225	0.018314017	0.001956154
K08963	mtnA	OK_nc	0.225	0.018314017	0.001956154
K07048	NA	OK_nc	0.225	0.018314017	0.001946099
K06168	miaB	OK_nc	0.225	0.018368754	0.001966257
K02926	RP-L4	OK_nc	0.225	0.018314017	0.001956154
K02314	dnaB	OK_nc	0.225	0.018176705	0.001916219
K00104	glcD	OK_nc	0.225	0.018314017	0.001946099
K18866	vanXY	OK_nc	0.224	0.018919332	0.002051202
K14977	ylbA	OK_nc	0.224	0.018629783	0.002007148
K07722	nikR	OK_nc	0.224	0.018919332	0.002059358
K05916	NA	OK_nc	0.224	0.018629783	0.002007148
K02459	gspJ	OK_nc	0.224	0.018919332	0.002064279
K01664	pabA	OK_nc	0.224	0.018919332	0.002059358
K01113	phoD	OK_nc	0.224	0.018614287	0.001996852
K00334	nuoE	OK_nc	0.224	0.018878575	0.002038326
K00216	entA	OK_nc	0.224	0.018919332	0.002064644
K11742	mdtI	OK_nc	0.223	0.019220915	0.002102009
K05016	CLCN7	OK_nc	0.223	0.019296195	0.002123654
K02968	RP-S20	OK_nc	0.223	0.019453665	0.002145492
K01436	yhaA	OK_nc	0.223	0.01923818	0.002112812
K18575	TMEM86B	OK_nc	0.222	0.01986716	0.002244969
K15827	NA	OK_nc	0.222	0.019781536	0.002222902
K15460	yfiC	OK_sd	0.222	0.019627015	0.002200991

KO_Id	Annotation	Deconfounding Status	Ds	p value	q value
K13893	yejA	OK_nc	0.222	0.019797007	0.002229227
K09949	lpxI	OK_nc	0.222	0.019627015	0.002189787
K08319	NA	OK_nc	0.222	0.019627015	0.002192532
K03169	topB	OK_nc	0.222	0.01986716	0.002246333
K02874	RP-L14	OK_nc	0.222	0.019627015	0.002178635
K00835	avtA	OK_nc	0.222	0.019887087	0.002257799
K09798	NA	OK_nc	0.221	0.020110895	0.00229253
K02825	pyrR	OK_nc	0.221	0.020110895	0.00229253
K02428	NA	OK_nc	0.221	0.020544684	0.0023515
K02335	polA	OK_nc	0.221	0.020172395	0.002304215
K15527	NA	OK_nc	0.22	0.021163255	0.002451721
K13421	UMPS	OK_nc	0.22	0.021008829	0.002424092
K08589	rgpA_B	OK_nc	0.22	0.021290148	0.002476638
K08219	UMF2	OK_nc	0.22	0.021290148	0.002486088
K03772	fkpA	OK_nc	0.22	0.021008829	0.002424096
K02895	RP-L24	OK_nc	0.22	0.021290148	0.002486153
K02031	ddpD	OK_nc	0.22	0.021073085	0.002436392
K01885	EARS	OK_nc	0.22	0.021290148	0.002486153
K07638	envZ	OK_nc	0.219	0.021662072	0.00254966
K07397	NA	OK_nc	0.219	0.02148705	0.002524081
K06714	rocR	OK_nc	0.219	0.021421177	0.00251138
K02496	hemX	OK_nc	0.219	0.021838416	0.002575476
K02035	ABC-PE-S	OK_nc	0.218	0.022108569	0.002627826
K01816	hyi	OK_nc	0.218	0.022495214	0.002694631
K00185	dmsC	OK_nc	0.218	0.022369799	0.002674424
K08736	MSH3	OK_nc	0.217	0.022785117	0.002739916
K08225	entS	OK_nc	0.217	0.022818259	0.002749188
K03733	xerC	OK_nc	0.217	0.023094092	0.002790766
K01792	E5-1-3-15	OK_nc	0.217	0.022785117	0.002735455
K01692	paaF	OK_nc	0.217	0.023145911	0.002804752
K01369	LGMN	OK_nc	0.217	0.023094092	0.002793122
K18855	NA	OK_nc	0.216	0.023905642	0.00291897
K12339	cysM	OK_nc	0.216	0.023758751	0.002890024
K11924	mntR	OK_nc	0.216	0.02393415	0.002933542
K10974	codB	OK_nc	0.216	0.024127942	0.002962884
K09989	NA	OK_nc	0.216	0.02393415	0.002933527
K13918	NA	OK_nc	0.215	0.024717403	0.00305245
K11933	NA	OK_nc	0.215	0.024641592	0.003037379
K11740	NA	OK_nc	0.215	0.024732393	0.003067571
K09946	NA	OK_nc	0.215	0.024732393	0.003082953
K07309	ynfE	OK_nc	0.215	0.024840184	0.003113656
K02462	gspM	OK_nc	0.215	0.024641592	0.003033746
K01011	TST	OK_nc	0.215	0.024732393	0.003082953

KO_Id	Annotation	Deconfounding Status	Ds	p value	q value
K00851	idnK	OK_nc	0.215	0.024763211	0.00309827
K16841	hpxA	OK_nc	0.214	0.025498839	0.003255297
K13014	arnD	OK_nc	0.214	0.025400337	0.003230951
K10752	RBBP4	OK_nc	0.214	0.0251932	0.003187092
K06198	NA	OK_nc	0.214	0.025307602	0.003207428
K03684	rnd	OK_nc	0.214	0.025150789	0.003175899
K02965	RP-S19	OK_nc	0.214	0.025150789	0.003175899
K01869	LARS	OK_nc	0.214	0.02538661	0.003223323
K01419	hslV	OK_nc	0.214	0.025150789	0.003175899
K13926	NA	OK_nc	0.213	0.026109033	0.003369493
K09020	rutB	OK_nc	0.213	0.025658417	0.003287559
K07343	NA	OK_nc	0.213	0.026190704	0.003386101
K03578	NA	OK_nc	0.213	0.025738622	0.003303799
K03304	tehA	OK_nc	0.213	0.026027613	0.003352955
K00239	sdhA	OK_nc	0.213	0.026272657	0.003402784
K11938	NA	OK_nc	0.212	0.026776241	0.003486619
K06351	NA	OK_nc	0.212	0.02669816	0.003470266
K04026	NA	OK_nc	0.212	0.026354895	0.003419541
K02363	entE	OK_nc	0.212	0.026986161	0.003538964
K01271	pepQ	OK_nc	0.212	0.026949549	0.003521674
K00883	dgoK	OK_nc	0.212	0.026949549	0.003521674
K13730	inlA	OK_nc	0.211	0.027432198	0.003680084
K13695	nlpC	OK_nc	0.211	0.027432198	0.003680084
K11003	hlyD	OK_nc	0.211	0.027402331	0.003641492
K09778	lpxJ	OK_nc	0.211	0.027402331	0.003644332
K06925	tsaE	OK_nc	0.211	0.027402331	0.003626575
K03836	tnaB	OK_nc	0.211	0.027432198	0.003679346
K03551	ruvB	OK_nc	0.211	0.027402331	0.003608897
K03311	NA	OK_nc	0.211	0.027432198	0.003680084
K03168	topA	OK_nc	0.211	0.027402331	0.003644332
K02886	RP-L2	OK_nc	0.211	0.027402331	0.003644332
K02565	nagC	OK_nc	0.211	0.027432198	0.003662168
K00591	COQ3	OK_nc	0.211	0.027402331	0.003644332
K00336	nuoG	OK_nc	0.211	0.027402331	0.003608897
K16012	cydC	OK_nc	0.21	0.027740588	0.00373431
K10011	arnA	OK_nc	0.21	0.027956327	0.003789261
K09954	NA	OK_nc	0.21	0.027740588	0.003732252
K08239	GNPTAB	OK_sd	0.21	0.027996815	0.003807723
K07639	rstB	OK_nc	0.21	0.028084904	0.00382621
K06883	NA	OK_nc	0.21	0.027996815	0.00380758
K06861	lptB	OK_nc	0.21	0.02840134	0.003882482
K06348	NA	OK_nc	0.21	0.02840134	0.003882482
K03748	NA	OK_nc	0.21	0.027780426	0.003752546

KO_Id	Annotation	Deconfounding Status	Ds	p value	q value
K14347	SLC10A7	OK_nc	0.209	0.028762678	0.003958458
K14053	ompG	OK_nc	0.209	0.029319475	0.004052838
K07803	zraP	OK_nc	0.209	0.028762678	0.003958534
K07786	emrY	OK_nc	0.209	0.028991131	0.003996692
K02032	ddpF	OK_nc	0.209	0.028491251	0.003901374
K01752	E4-3-1-17	OK_nc	0.209	0.028581467	0.00392035
K07109	NA	OK_sd	0.208	0.029958192	0.004164716
K03799	htpX	OK_nc	0.208	0.029980599	0.004174778
K02381	NA	OK_nc	0.208	0.030216132	0.004235579
K01972	E6-5-1-2	OK_nc	0.208	0.030120702	0.004215223
K00857	tdk	OK_nc	0.208	0.029554787	0.00409494
K00335	nuoF	OK_nc	0.208	0.029791814	0.004134684
K16234	hutT	OK_nc	0.207	0.030308091	0.004256024
K13574	hcxB	OK_nc	0.207	0.030721541	0.004349134
K11208	NA	OK_nc	0.207	0.030308091	0.004273131
K07590	RP-L7A	OK_nc	0.207	0.030308091	0.004276559
K00590	E2-1-1-113	OK_nc	0.207	0.030308091	0.004276559
K17752	rsbT	OK_nc	0.206	0.031533858	0.004551806
K16328	psuK	OK_nc	0.206	0.031338033	0.004465452
K15584	nikA	OK_nc	0.206	0.031684317	0.004595547
K12962	arnE	OK_nc	0.206	0.031533858	0.0045518
K10680	nemA	OK_nc	0.206	0.031533858	0.004540877
K09800	tamB	OK_nc	0.206	0.031435272	0.004508441
K03635	MOCS2B	OK_nc	0.206	0.031583654	0.004573629
K03146	THI4	OK_nc	0.206	0.03136185	0.004476112
K03119	tauD	OK_nc	0.206	0.031557429	0.00456252
K18331	NA	OK_nc	0.205	0.03198865	0.004661874
K17215	K17215	OK_nc	0.205	0.032141716	0.004706572
K10539	araG	OK_nc	0.205	0.032296306	0.004751658
K10212	K10212	OK_nc	0.205	0.03198865	0.004663438
K02621	parC	OK_nc	0.205	0.032296306	0.004751658
K16695	wzxC	OK_nc	0.204	0.033392138	0.004959305
K15832	NA	OK_nc	0.204	0.032918542	0.004866087
K09979	NA	OK_sd	0.204	0.032866255	0.004850743
K07746	parD1_3_4	OK_nc	0.204	0.033480847	0.004987995
K07492	NA	OK_nc	0.204	0.033286711	0.004935935
K07482	NA	OK_nc	0.204	0.033286711	0.004935935
K02348	NA	OK_nc	0.204	0.033480847	0.004983003
K18815	aac6-I	OK_nc	0.203	0.033929096	0.005078335
K15836	fhlA	OK_nc	0.203	0.034093423	0.005126652
K11733	lysP	OK_nc	0.203	0.033929096	0.005078359
K09472	puuC	OK_nc	0.203	0.034320982	0.005224487
K08697	NA	OK_nc	0.203	0.034320982	0.005199754

KO_Id	Annotation	Deconfounding Status	Ds	p value	q value
K05878	dhaK	OK_nc	0.203	0.034320982	0.005224487
K03475	ulaA	OK_nc	0.203	0.034320982	0.005224487
K02033	ABC-PE-P	OK_nc	0.203	0.034320982	0.005224487
K01337	E3-4-21-50	OK_nc	0.203	0.034118316	0.005138301
K00632	fadA	OK_nc	0.203	0.034320982	0.005199871
K18898	mdtE	OK_nc	0.202	0.035365787	0.005424502
K16457	CEP76	OK_nc	0.202	0.035646117	0.005475759
K11165	NA	OK_nc	0.202	0.035251568	0.005390648
K07806	arnB	OK_nc	0.202	0.035033284	0.005349151
K04027	NA	OK_nc	0.202	0.035257762	0.005399764
K03274	gmhD	OK_nc	0.202	0.035033284	0.005349151
K18140	envR	OK_nc	0.201	0.036317665	0.005629406
K15024	K15024	OK_nc	0.201	0.035764881	0.005502289
K07755	AS3MT	OK_nc	0.201	0.036224477	0.005606562
K02466	NA	OK_nc	0.201	0.036518656	0.005685945
K01881	PARS	OK_nc	0.201	0.036048064	0.005554208
K00184	dmsB	OK_nc	0.201	0.036518656	0.005685945
K17732	PMPCB	OK_nc	0.2	0.037464105	0.005902593
K15503	ANKRD44	OK_nc	0.2	0.03752889	0.005930191
K03293	NA	OK_nc	0.2	0.03752889	0.005930191
K01624	FBA	OK_nc	0.2	0.036870359	0.005766333
K00113	glpC	OK_nc	0.2	0.036988348	0.005793356
K08172	shiA	OK_nc	0.199	0.03858089	0.006212605
K06997	NA	OK_nc	0.199	0.03813366	0.006069932
K06180	rluD	OK_nc	0.199	0.038445461	0.006155193
K06048	gshA	OK_nc	0.199	0.038252262	0.006097673
K03335	iolE	OK_nc	0.199	0.037945989	0.006013683
K02996	RP-S9	OK_nc	0.199	0.03813366	0.006069932
K02562	mtlR	OK_nc	0.199	0.03858089	0.006212395
K01281	pepX	OK_nc	0.199	0.038568651	0.006183852
K18899	mdtF	OK_nc	0.198	0.038870395	0.006358318
K12525	metL	OK_nc	0.198	0.038607281	0.006270416
K10537	araF	OK_nc	0.198	0.039081881	0.006447243
K09927	NA	OK_nc	0.198	0.039081881	0.006417487
K09251	patA	OK_nc	0.198	0.038621424	0.006299695
K08276	eco	OK_nc	0.198	0.038740753	0.006328135
K07274	mipA	OK_nc	0.198	0.038621424	0.006299695
K05875	tar	OK_nc	0.198	0.039081881	0.006447215
K04046	yegD	OK_nc	0.198	0.038607281	0.00625602
K01577	oxc	OK_nc	0.198	0.038607281	0.00625602
K01486	ade	OK_nc	0.198	0.038607281	0.006241532
K17792	TIM54	OK_nc	0.197	0.03923456	0.00648152
K15974	emrR	OK_nc	0.197	0.040119596	0.006720654

KO_Id	Annotation	Deconfounding Status	Ds	p value	q value
K07566	tsaC	OK_nc	0.197	0.0401016	0.006689794
K03841	FBP	OK_nc	0.197	0.039605707	0.006597892
K03782	katG	OK_nc	0.197	0.040193568	0.006751693
K03737	por	OK_nc	0.197	0.040119596	0.006720683
K03177	truB	OK_nc	0.197	0.039478245	0.006567512
K02781	srlB	OK_nc	0.197	0.039461258	0.006537257
K02034	ABC-PE-P1	OK_nc	0.197	0.039334399	0.006507127
K00880	lyxK	OK_nc	0.197	0.039478245	0.006567512
K00384	trxB	OK_nc	0.197	0.039478245	0.006567512
K19332	MKS1	OK_nc	0.196	0.040528508	0.006845524
K17214	K17214	OK_nc	0.196	0.040908575	0.007004504
K12297	rlmKL	OK_nc	0.196	0.040985659	0.007036694
K10804	tesA	OK_nc	0.196	0.040887326	0.006972445
K09937	NA	OK_nc	0.196	0.040528508	0.006842868
K07336	NA	OK_nc	0.196	0.040528508	0.006828604
K05882	NA	OK_nc	0.196	0.040908575	0.007004504
K04113	bcrB	OK_nc	0.196	0.040790762	0.006908723
K04063	NA	OK_nc	0.196	0.040908575	0.006988384
K02584	nifA	OK_nc	0.196	0.040887326	0.006972445
K00946	thiL	OK_nc	0.196	0.040887326	0.006972445
K00231	PPOX	OK_nc	0.196	0.040985659	0.007036694
K18142	acrF	OK_nc	0.195	0.041440779	0.007134036
K16951	asrB	OK_nc	0.195	0.041923915	0.007265776
K16209	lacS	OK_nc	0.195	0.041575094	0.007166791
K15722	NA	OK_nc	0.195	0.04159441	0.007179758
K00657	speG	OK_nc	0.195	0.041923915	0.007265776
K00313	NA	OK_nc	0.195	0.04111844	0.007069018
K11687	NA	OK_nc	0.194	0.043047028	0.007570133
K10972	allS	OK_nc	0.194	0.042485551	0.0073828
K07114	yfbK	OK_nc	0.194	0.042769606	0.007501527
K06149	NA	OK_nc	0.194	0.042525896	0.007399664
K05876	trg	OK_nc	0.194	0.04263154	0.007465717
K04749	rsbV	OK_nc	0.194	0.042908101	0.00753576
K03229	yscU	OK_nc	0.194	0.043337123	0.007651272
K03089	rpoH	OK_nc	0.194	0.042606769	0.007433479
K02806	ptsN	OK_nc	0.194	0.042606769	0.007433479
K00986	NA	OK_nc	0.194	0.04332618	0.007639301
K00929	buk	OK_nc	0.194	0.04263154	0.007467434
K11066	E3-5-1-28D	OK_nc	0.193	0.044430845	0.00795761
K08159	sotB	OK_nc	0.193	0.043947154	0.007779339
K06159	yojI	OK_nc	0.193	0.043974093	0.007796722
K03762	proP	OK_nc	0.193	0.044287482	0.007921672
K03602	xseB	OK_nc	0.193	0.044144516	0.007885872

KO_Id	Annotation	Deconfounding Status	Ds	p value	q value
K03048	rpoE	OK_nc	0.193	0.044059211	0.007850216
K02626	pdaD	OK_nc	0.193	0.043974093	0.007814027
K01866	YARS	OK_nc	0.193	0.044059211	0.007850216
K01407	ptrA	OK_nc	0.193	0.043974093	0.007814673
K17624	NA	OK_nc	0.192	0.045215358	0.008213355
K16868	NA	OK_nc	0.192	0.044516139	0.00799371
K15778	pmm-pgm	OK_nc	0.192	0.045126907	0.008176377
K11735	gabP	OK_nc	0.192	0.044516139	0.007993545
K09988	lyxA	OK_nc	0.192	0.0445466	0.008029948
K09934	NA	OK_nc	0.192	0.044516139	0.008003829
K09013	sufC	OK_nc	0.192	0.045451053	0.008287761
K06127	COQ5	OK_nc	0.192	0.045359298	0.008250011
K02867	RP-L11	OK_nc	0.192	0.0445466	0.008029948
K01825	fadB	OK_nc	0.192	0.045081205	0.008157651
K00112	glpB	OK_nc	0.192	0.045215358	0.008213355
K15834	NA	OK_nc	0.191	0.04666702	0.0086068
K14287	ybdL	OK_nc	0.191	0.045592801	0.008324172
K08317	NA	OK_nc	0.191	0.045778494	0.008400502
K07315	rsbU_P	OK_nc	0.191	0.045688249	0.00836277
K06910	PEBP	OK_nc	0.191	0.04664241	0.008591454
K04028	NA	OK_nc	0.191	0.045688249	0.00836277
K02445	glpT	OK_nc	0.191	0.04664241	0.008591454
K00641	metX	OK_nc	0.191	0.045778494	0.008398623
K00057	gpsA	OK_nc	0.191	0.046550515	0.008552956
K18890	mdlB	OK_nc	0.19	0.047469609	0.008864807
K15737	csiD	OK_nc	0.19	0.047352408	0.008805886
K14575	AFG2	OK_nc	0.19	0.04747344	0.008876521
K13654	mcbR	OK_nc	0.19	0.047352408	0.008810005
K13635	cbl	OK_nc	0.19	0.047308143	0.008746691
K08350	fdnI	OK_nc	0.19	0.047417143	0.008844022
K04035	E1-14-13-81	OK_nc	0.19	0.047352408	0.008805665
K03282	mscL	OK_nc	0.19	0.047875831	0.008985038
K02933	RP-L6	OK_nc	0.19	0.047352408	0.008786277
K02475	K02475	OK_nc	0.19	0.047308143	0.008746964
K01890	FARSB	OK_nc	0.19	0.047875831	0.008985038
K01810	GPI	OK_nc	0.19	0.047352408	0.008786277
K00939	adk	OK_nc	0.19	0.047377827	0.008825712
K15828	NA	OK_nc	0.189	0.04878939	0.009269532
K10017	hisP	OK_nc	0.189	0.04846609	0.00916319
K10015	hisM	OK_nc	0.189	0.047971748	0.009024186
K10003	gltJ	OK_nc	0.189	0.048634544	0.009227743
K09338	HD-ZIP	OK_nc	0.189	0.048536797	0.009187804
K07661	rstA	OK_nc	0.189	0.049102821	0.009351835

KO_Id	Annotation	Deconfounding Status	Ds	p value	q value
K05368	fre	OK_nc	0.189	0.049148228	0.009372995
K04093	pheA1	OK_nc	0.189	0.047981818	0.00903828
K01146	NA	OK_nc	0.189	0.048417466	0.009142779
K16302	CNNM	OK_nc	0.188	0.04940745	0.009478538
K11201	frvA	OK_nc	0.188	0.049148228	0.009394645
K10002	gltK	OK_nc	0.188	0.049507817	0.009520734
K03786	aroQ	OK_nc	0.188	0.049307469	0.009436508
K02782	srlE	OK_nc	0.188	0.049930686	0.00964833
K01775	alr	OK_nc	0.188	0.049148228	0.009394645
K00825	AADAT	OK_nc	0.188	0.04960857	0.009563098
K02377	TSTA3	OK_nc	-0.188	0.04960857	0.009563098
K01730	ogl	OK_nc	-0.188	0.049507817	0.009520734
K01202	GALC	OK_nc	-0.188	0.04940745	0.009478444
K00661	NA	OK_nc	-0.188	0.049930686	0.00964833
K19075	cst2	OK_nc	-0.189	0.049045285	0.009329513
K15771	ganP	OK_nc	-0.189	0.047971748	0.009025269
K06113	NA	OK_nc	-0.189	0.048068045	0.00906566
K01582	E4-1-1-18	OK_nc	-0.189	0.048634544	0.009228844
K02529	lacI	OK_nc	-0.19	0.047875831	0.008985038
K10206	E2-6-1-83	OK_nc	-0.192	0.044691007	0.008066333
K07814	NA	OK_nc	-0.192	0.045451053	0.008287761
K02662	pilM	OK_nc	-0.192	0.045038827	0.008139547
K13924	cheBR	OK_nc	-0.193	0.044144516	0.007885872
K00587	ICMT	OK_nc	-0.193	0.043606889	0.007709003
K03519	NA	OK_nc	-0.194	0.04263154	0.007467434
K01187	malZ	OK_nc	-0.194	0.043186387	0.007604647
K15772	ganQ	OK_nc	-0.195	0.042252116	0.007332445
K09474	phoN	OK_nc	-0.195	0.041923915	0.007265776
K18581	NA	OK_nc	-0.196	0.040659429	0.006877058
K11063	tcdAB	OK_nc	-0.196	0.040887326	0.006972445
K03779	ttdA	OK_nc	-0.196	0.040887326	0.006940518
K00754	NA	OK_nc	-0.196	0.040323362	0.006782845
K17360	ACOT7	OK_nc	-0.197	0.040119596	0.006720683
K02477	K02477	OK_nc	-0.197	0.040193568	0.0067517
K18785	NA	OK_nc	-0.198	0.038607281	0.006270552
K11257	NA	OK_nc	-0.198	0.039081881	0.006432342
K09153	NA	OK_nc	-0.198	0.038621424	0.006299695
K01811	NA	OK_nc	-0.198	0.039081881	0.006447243
K00059	fabG	OK_nc	-0.198	0.038995728	0.006387854
K00046	NA	OK_nc	-0.198	0.038607281	0.006270552
K15971	tcmP	OK_nc	-0.199	0.038322661	0.00612652
K09707	NA	OK_nc	-0.199	0.03813366	0.006069932
K07706	agrC	OK_nc	-0.199	0.038322661	0.006126653

KO_Id	Annotation	Deconfounding Status	Ds	p value	q value
K06871	NA	OK_nc	-0.199	0.03858089	0.006212632
K12452	ascC	OK_nc	-0.2	0.037106716	0.005820493
K05349	bglX	OK_nc	-0.2	0.037649073	0.005957905
K02508	hpaA	OK_nc	-0.2	0.037225464	0.005847744
K01785	galM	OK_nc	-0.2	0.036870359	0.005766333
K01740	metY	OK_nc	-0.2	0.036807359	0.005739424
K01686	uxuA	OK_nc	-0.2	0.037344594	0.005875111
K13052	divIC	OK_nc	-0.201	0.036456505	0.005659374
K05601	hcp	OK_nc	-0.201	0.036224477	0.005606569
K00937	ppk1	OK_nc	-0.201	0.036224477	0.005606569
K17881	aadB	OK_nc	-0.203	0.033984862	0.005102454
K01992	ABC-2-P	OK_nc	-0.203	0.033929096	0.005078359
K00936	pdtaS	OK_nc	-0.203	0.033984862	0.005102454
K00284	GLU	OK_nc	-0.203	0.034320982	0.005199871
K15770	cycB	OK_nc	-0.205	0.032039458	0.004684175
K11244	WSC	OK_nc	-0.205	0.03198865	0.004669335
K10193	togM	OK_nc	-0.205	0.032709438	0.004820019
K00100	bdhAB	OK_nc	-0.205	0.032244312	0.004729066
K19081	braS	OK_nc	-0.206	0.031435272	0.004508441
K18650	NA	OK_nc	-0.206	0.03123896	0.004444097
K11072	potA	OK_nc	-0.206	0.03123896	0.004444097
K02110	ATPF0C	OK_nc	-0.206	0.031785314	0.004617561
K01815	kduI	OK_nc	-0.206	0.031435272	0.004508441
K00700	GBE1	OK_nc	-0.206	0.031533858	0.004551806
K17675	SUPV3L1	OK_nc	-0.207	0.030550894	0.004317897
K06182	rluF	OK_nc	-0.207	0.031041798	0.004401664
K18579	NA	OK_nc	-0.208	0.030120702	0.004215223
K02078	acpP	OK_nc	-0.208	0.030120702	0.004215223
K00784	rnz	OK_nc	-0.209	0.029319475	0.004055544
K11069	potD	OK_nc	-0.21	0.027780426	0.003752546
K07024	SPP	OK_nc	-0.21	0.027868227	0.003770863
K01963	accD	OK_nc	-0.212	0.026986161	0.003538964
K11444	wspR	OK_nc	-0.213	0.025946538	0.003336499
K01681	ACO	OK_nc	-0.213	0.025658417	0.003287559
K19090	cas5t	OK_nc	-0.214	0.025019727	0.003141958
K05012	CLCN3_4_5	OK_nc	-0.214	0.025419466	0.003239274
K06989	nadX	OK_nc	-0.215	0.024732393	0.003082953
K02438	glgX	OK_nc	-0.215	0.024732393	0.003067706
K01812	uxaC	OK_nc	-0.215	0.024763211	0.00309827
K11071	potB	OK_nc	-0.216	0.023567838	0.002861341
K01696	trpB	OK_nc	-0.216	0.023832066	0.002904464
K10706	SETX	OK_nc	-0.218	0.022176719	0.002641064
K01711	gmd	OK_nc	-0.218	0.022245115	0.002654364

KO_Id	Annotation	Deconfounding Status	Ds	p value	q value
K01193	INV	OK_nc	-0.218	0.022108569	0.002627826
K19309	bcrA	OK_nc	-0.219	0.021421177	0.00251138
K04034	bchE	OK_nc	-0.219	0.021973004	0.00260153
K00972	UAP1	OK_nc	-0.219	0.021845839	0.002581413
K02502	hisZ	OK_nc	-0.22	0.020817465	0.002387545
K00978	rfbF	OK_nc	-0.22	0.021008829	0.002424096
K10041	peb1C	OK_nc	-0.222	0.019887087	0.002257802
K06987	NA	OK_nc	-0.222	0.019627015	0.002178635
K05992	amyM	OK_nc	-0.222	0.019627015	0.002200991
K03534	NA	OK_nc	-0.222	0.019627015	0.002200991
K06923	NA	OK_nc	-0.223	0.019612336	0.002167536
K00031	IDH1	OK_nc	-0.223	0.01923818	0.002112812
K14158	SLC5A1	OK_nc	-0.225	0.017982842	0.001891615
K07407	E3-2-1-22B	OK_nc	-0.226	0.017804744	0.001848129
K04115	bcrD	OK_nc	-0.226	0.017923179	0.001877034
K02302	cysG	OK_nc	-0.226	0.017792231	0.001838586
K06972	PITRM1	OK_nc	-0.227	0.017313196	0.001773038
K02114	ATPF1E	OK_nc	-0.227	0.017364044	0.001782269
K05946	tagA	OK_nc	-0.228	0.016797449	0.001700761
K02488	pleD	OK_nc	-0.228	0.016651042	0.001674363
K01817	trpF	OK_nc	-0.228	0.016786955	0.00169192
K16212	NA	OK_nc	-0.229	0.016184137	0.001597415
K18968	adrA	OK_nc	-0.23	0.015405459	0.001492002
K01567	NA	OK_nc	-0.23	0.015583968	0.001523734
K02004	ABC-CD-P	OK_nc	-0.231	0.015088187	0.00142282
K00041	uxaB	OK_nc	-0.231	0.015405459	0.001476366
K17680	PEO1	OK_nc	-0.232	0.015088187	0.001415319
K12604	CNOT1	OK_nc	-0.232	0.015030819	0.001393032
K11624	ydfI	OK_nc	-0.232	0.015030819	0.00139279
K10040	peb1B	OK_nc	-0.232	0.014898506	0.001356606
K02483	K02483	OK_nc	-0.232	0.015030819	0.001393032
K00018	hprA	OK_nc	-0.234	0.013974248	0.001265971
K06311	yndE	OK_nc	-0.235	0.013383976	0.001199878
K17046	DEK	OK_nc	-0.236	0.013235216	0.001168353
K02321	POLA2	OK_nc	-0.236	0.013235832	0.001171475
K01854	glf	OK_nc	-0.236	0.012950564	0.001125223
K01488	add	OK_nc	-0.236	0.013161918	0.001149686
K19265	NA	OK_nc	-0.237	0.012950009	0.001107192
K18675	chbP	OK_nc	-0.237	0.012881215	0.001095321
K10120	msmE	OK_nc	-0.237	0.012641561	0.001066156
K01657	trpE	OK_nc	-0.237	0.012915653	0.001101242
K00651	metA	OK_nc	-0.237	0.012709023	0.001077735
K06401	NA	OK_nc	-0.238	0.012504617	0.001048812

KO_Id	Annotation	Deconfounding Status	Ds	p value	q value
K03610	minC	OK_nc	-0.238	0.012540085	0.001054692
K01195	uidA	OK_nc	-0.239	0.012055018	0.000977585
K01190	lacZ	OK_nc	-0.239	0.012281686	0.001004503
K18197	NA	OK_nc	-0.24	0.012026704	0.000961756
K10194	togN	OK_nc	-0.24	0.012026704	0.000961756
K01695	trpA	OK_nc	-0.24	0.011753358	0.000920699
K00690	E2-4-1-7	OK_nc	-0.24	0.011970746	0.000951336
K11991	tadA	OK_nc	-0.241	0.011547947	0.000890979
K14051	gmr	OK_nc	-0.242	0.011139128	0.00083879
K00765	hisG	OK_nc	-0.243	0.010964049	0.000820525
K00573	NA	OK_nc	-0.243	0.010799659	0.000798214
K07813	agrB	OK_nc	-0.244	0.010663955	0.000780772
K02113	ATPF1D	OK_nc	-0.244	0.010714647	0.000789448
K01962	accA	OK_nc	-0.245	0.010365939	0.000746943
K13631	soxS	OK_nc	-0.247	0.00925591	0.000649801
K17768	TOM70	OK_nc	-0.248	0.00906374	0.00061951
K17472	cymR	OK_nc	-0.248	0.009234702	0.000646172
K07011	NA	OK_nc	-0.248	0.009130526	0.000628305
K00874	kdgK	OK_nc	-0.248	0.00906374	0.000617807
K03716	NA	OK_nc	-0.249	0.00906374	0.000610899
K03436	fruR2	OK_nc	-0.249	0.00906374	0.000614344
K02108	ATPF0A	OK_nc	-0.251	0.008175759	0.000530401
K11755	hisIE	OK_nc	-0.252	0.008107806	0.000518479
K11718	HUGT	OK_nc	-0.254	0.007605008	0.000464598
K02421	fliR	OK_nc	-0.254	0.007605008	0.000465181
K01687	ilvD	OK_nc	-0.254	0.007619838	0.000467854
K01209	abfA	OK_nc	-0.254	0.007605008	0.000465181
K01051	E3-1-1-11	OK_nc	-0.254	0.007605008	0.000465181
K09690	wzm	OK_nc	-0.255	0.007376288	0.000439228
K02385	flbD	OK_nc	-0.257	0.006812427	0.000399338
K17808	ZIM17	OK_nc	-0.258	0.006672581	0.000386503
K00394	aprA	OK_nc	-0.258	0.006527607	0.000373568
K19167	abiQ	OK_nc	-0.262	0.005611626	0.000299044
K15532	NA	OK_nc	-0.262	0.005680608	0.000307985
K02400	flhA	OK_nc	-0.262	0.005680608	0.000304379
K09691	wzt	OK_nc	-0.264	0.005234075	0.000275286
K04751	glnB	OK_nc	-0.264	0.005234075	0.000275286
K01953	asnB	OK_nc	-0.265	0.004999101	0.00025482
K00729	ALG5	OK_nc	-0.265	0.005006209	0.000256342
K11682	SWC3	OK_nc	-0.268	0.004542576	0.000219972
K10192	togB	OK_nc	-0.269	0.00438739	0.000205341
K00385	asrC	OK_nc	-0.269	0.004465964	0.000214192
K02408	fliE	OK_nc	-0.27	0.004294943	0.000198029

KO_Id	Annotation	Deconfounding Status	Ds	p value	q value
K00052	leuB	OK_nc	-0.27	0.004378067	0.000202876
K02409	fliF	OK_nc	-0.271	0.004175668	0.00018866
K01371	CTSK	OK_nc	-0.271	0.004221924	0.000192706
K06882	NA	OK_nc	-0.272	0.004043283	0.000180805
K00766	trpD	OK_nc	-0.272	0.004015176	0.000178618
K02401	flhB	OK_nc	-0.273	0.003861104	0.00016908
K01563	dhaA	OK_nc	-0.273	0.003891283	0.000172205
K02419	fliP	OK_nc	-0.274	0.003770498	0.000161995
K01653	E2-2-1-6S	OK_nc	-0.274	0.003770498	0.000161006
K07705	lytT	OK_nc	-0.275	0.003719002	0.00015424
K01647	CS	OK_nc	-0.277	0.003366261	0.000138051
K18475	fliB	OK_nc	-0.278	0.003275183	0.000129763
K02411	fliH	OK_nc	-0.278	0.003212656	0.000125797
K02414	fliK	OK_nc	-0.28	0.00296188	0.000115291
K14777	DDX47	OK_nc	-0.282	0.002727195	0.000102351
K03413	cheY	OK_nc	-0.282	0.002727195	0.000102996
K01129	dgt	OK_nc	-0.282	0.002726349	0.000100438
K00575	cheR	OK_nc	-0.282	0.002726349	0.000100438
K00817	hisC	OK_nc	-0.283	0.002676027	9.61E-05
K03412	cheB	OK_nc	-0.286	0.002345875	8.10E-05
K03327	TC-MATE	OK_nc	-0.287	0.002188436	7.45E-05
K02372	fabZ	OK_nc	-0.287	0.002188436	7.45E-05
K01442	cbh	OK_nc	-0.29	0.001928197	6.39E-05
K02415	fliL	OK_nc	-0.291	0.001848918	5.91E-05
K01703	leuC	OK_nc	-0.291	0.001883769	6.07E-05
K02412	fliI	OK_nc	-0.292	0.001769696	5.58E-05
K03706	codY	OK_nc	-0.295	0.001623204	4.89E-05
K02048	cysP	OK_nc	-0.296	0.001568153	4.61E-05
K02416	fliM	OK_nc	-0.297	0.001479014	4.18E-05
K00053	ilvC	OK_nc	-0.297	0.001491877	4.32E-05
K02420	fliQ	OK_nc	-0.298	0.001426275	3.94E-05
K17737	dhcR	OK_nc	-0.299	0.00142194	3.89E-05
K02556	motA	OK_nc	-0.299	0.00142194	3.86E-05
K01731	pelW	OK_nc	-0.299	0.001399307	3.76E-05
K00013	hisD	OK_nc	-0.299	0.00138367	3.69E-05
K02413	fliJ	OK_nc	-0.301	0.001320508	3.40E-05
K02410	fliG	OK_nc	-0.301	0.001309134	3.34E-05
K01814	hisA	OK_nc	-0.303	0.001180347	2.90E-05
K02406	fliC	OK_nc	-0.304	0.0011678	2.84E-05
K02388	flgC	OK_nc	-0.304	0.001147714	2.77E-05
K13815	rpfG	OK_nc	-0.306	0.001056983	2.47E-05
K01666	mhpE	OK_nc	-0.308	0.000947908	2.15E-05
K02390	flgE	OK_nc	-0.309	0.000947908	2.12E-05

KO_Id	Annotation	Deconfounding Status	Ds	p value	q value
K12992	rfbN	OK_nc	-0.31	0.000877172	1.89E-05
K01704	leuD	OK_nc	-0.311	0.000848523	1.79E-05
K16707	NA	OK_nc	-0.312	0.000806377	1.68E-05
K15889	PCME	OK_nc	-0.314	0.000752012	1.52E-05
K06310	NA	OK_nc	-0.314	0.000753773	1.54E-05
K03409	cheX	OK_nc	-0.316	0.000660496	1.29E-05
K02387	flgB	OK_nc	-0.316	0.000680709	1.36E-05
K00395	aprB	OK_nc	-0.316	0.000660496	1.30E-05
K03154	thiS	OK_nc	-0.318	0.000599381	1.14E-05
K06603	flaG	OK_nc	-0.319	0.000599381	1.13E-05
K02417	fliN	OK_nc	-0.319	0.000584863	1.08E-05
K02404	flhF	OK_nc	-0.32	0.000568239	1.01E-05
K01652	E2-2-1-6L	OK_nc	-0.32	0.000568239	1.00E-05
K04562	flhG	OK_nc	-0.322	0.00052654	9.15E-06
K02389	flgD	OK_nc	-0.324	0.000459287	7.77E-06
K01096	pgpB	OK_nc	-0.324	0.000459287	7.77E-06
K03410	cheC	OK_nc	-0.325	0.000459287	7.71E-06
K01198	xynB	OK_nc	-0.325	0.000455584	7.39E-06
K13626	fliW	OK_nc	-0.326	0.000433408	6.83E-06
K02397	flgL	OK_nc	-0.326	0.000442735	7.08E-06
K09770	NA	OK_nc	-0.327	0.000433402	6.73E-06
K02418	fliO	OK_nc	-0.327	0.000424618	6.49E-06
K03408	cheW	OK_nc	-0.33	0.000365502	5.42E-06
K01586	lysA	OK_nc	-0.33	0.00037047	5.58E-06
K02407	fliD	OK_nc	-0.331	0.00035341	5.04E-06
K02398	flgM	OK_nc	-0.331	0.000357695	5.22E-06
K02422	fliS	OK_nc	-0.333	0.000324094	4.36E-06
K02392	flgG	OK_nc	-0.334	0.000320254	4.23E-06
K03415	cheV	OK_nc	-0.336	0.000296143	3.71E-06
K16511	NA	OK_nc	-0.338	0.000269216	3.24E-06
K03411	cheD	OK_nc	-0.339	0.000252966	2.99E-06
K03320	amt	OK_nc	-0.34	0.000248599	2.88E-06
K02396	flgK	OK_nc	-0.342	0.000218055	2.43E-06
K10844	ERCC2	OK_nc	-0.345	0.0002003	1.95E-06
K06320	NA	OK_nc	-0.347	0.000178991	1.70E-06
K02045	cysA	OK_nc	-0.348	0.000178991	1.67E-06
K09766	NA	OK_nc	-0.349	0.000170713	1.54E-06
K03406	mcp	OK_nc	-0.352	0.000143308	1.26E-06
K04061	flhB2	OK_nc	-0.355	0.000117635	1.01E-06
K09749	NA	OK_nc	-0.357	0.000112831	8.37E-07
K06331	NA	OK_nc	-0.359	0.000108552	7.27E-07
K02500	hisF	OK_nc	-0.359	0.000108552	7.44E-07
K03563	csrA	OK_nc	-0.363	8.95E-05	5.47E-07

KO_Id	Annotation	Deconfounding Status	Ds	p value	q value
K02047	cysW	OK_nc	-0.366	8.95E-05	4.63E-07
K02046	cysU	OK_nc	-0.367	8.95E-05	4.24E-07
K06404	NA	OK_nc	-0.377	6.89E-05	2.09E-07
K02501	hisH	OK_nc	-0.378	6.65E-05	1.85E-07
K06403	NA	OK_nc	-0.38	6.21E-05	1.58E-07
K07216	NA	OK_nc	-0.381	6.21E-05	1.50E-07
K07502	NA	OK_nc	-0.387	5.26E-05	9.75E-08
K13114	PNN	OK_nc	-0.404	1.85E-05	2.57E-08
K02278	cpaA	OK_nc	-0.42	6.06E-06	7.02E-09

Supplementary Table S7. Sensitivity and specificity for the detection of CRC in the discovery and validation cohorts. The POD score cutoff was set at 0.642.

	Discovery		Validation	
	**Stage I/II (95%CI)**	**Stage III/IV (95%CI)**	**Stage I/II (95%CI)**	**Stage III/IV (95%CI)**
**Sensitivity**	66.7% (50.0%-83.3%)	90.9% (81.8%-97.0%)	87.5% (75.0%-100%)	95.7% (87.0%-100%)
**Specificity**	90.4% (82.7%-96.2%)		88.6% (79.5%-95.5%)	

Supplementary Table S8. General information of ten metagenomic studies.

Study name	Ethnicity	Sample	Age	DNA extraction	Sequencing platform	Metagenome No. (CRC vs control)	Sequence alignment program	Microbial sequence database	Modelling method	AUROC value (discovery and validation)	Reference
ZellerG_2014	French	Stool	(25-87)	Gnome	Illumina HiSeq (PE100)	114 (53 vs 61)	MOCAT	mOTU profile	LASSO	0.84 and 0.85	[1]
FengQ_2015	Austrians	Stool	(43-86)	MoBio	Illumina HiSeq	107 (46 vs 61)	in-house pipeline	IMG database	RF	0.96 and NA	[2]
YuJ_2015	Chinese	Stool	(34-89)	Qiagen	Illumina HiSeq	128 (74 vs 54)	MOCAT	mOTU profile	RF	0.96 and NA	[3]
VogtmannE_2016	Americans	Stool	(31-89)	Gnome	Illumina HiSeq (PE100)	104 (52 vs 52)	MOCAT	mOTU profile	NA	NA	[4]
ThomasAM_2018	Italians	Stool	(57-84)	Qiagen	Illumina HiSeq	53 (29 vs 24)	MOCAT	mOTU profile	LASSO	NA	[5]
WirbelJ_2018	German	Stool	(28-87)	Gnome	Illumina HiSeq	125 (60 vs 65)	MOCAT	mOTU profile	LASSO	0.88 and 0.83	[6]
ThomasAM_2019	Japanese	Stool	(32-78)	NA	Illumina HiSeq	80 (40 vs 40)	MOCAT	mOTU profile	LASSO	NA	[5]
GuptaA_2019	Indians	Stool	(22-75)	Qiagen	Illumina NextSeq	60 (30 vs 30)	NA	NA	NA	NA	[7]
YachidaS_2019	Japanese	Stool	(21-79)	NA	Illumina HiSeq	509 (258 vs 251)	BLAST	LTP of the SILVA database	LASSO/RF	0.83 and NA	[8]
KLM_2022	Chinese	Stool	(41-79)	Magen	MGISEQ-2000（PE150）	284 (188 vs 96)	BWA mem	GTDB	LASSO	0.94 and 0.91	Current study

IMG: Integrated Microbial Genome

.

Supplementary Table S9. Bacterial species enriched or depleted across ten metagenomic studies.

species	genus	I2	qtest	pval	beta	beta_lb	beta_ub
Faecalibacterium_prausnitzii	Faecalibacterium	43.818	15.505	5.48E-06	-0.039692	-0.056807	-0.022577
Eubacterium_eligens	Eubacterium	14.739	11.339	1.18E-06	-0.024945	-0.035008	-0.014883
Roseburia_intestinalis	Roseburia	15.114	11.279	1.59E-08	-0.021509	-0.028969	-0.014050
Bifidobacterium_adolescentis	Bifidobacterium	68.507	21.121	2.24E-02	-0.020191	-0.037518	-0.002864
Roseburia_hominis	Roseburia	0.000	4.080	4.89E-03	-0.006090	-0.010332	-0.001848
Eubacterium_ventriosum	Eubacterium	56.525	21.196	1.34E-02	-0.005363	-0.009614	-0.001113
Parasutterella_excrementihominis	Parasutterella	14.144	6.141	1.22E-02	-0.004732	-0.008432	-0.001031
Adlercreutzia_equolifaciens	Adlercreutzia	28.447	13.550	7.15E-03	-0.003370	-0.005826	-0.000915
Streptococcus_thermophilus	Streptococcus	13.823	12.159	4.26E-02	-0.001843	-0.003624	-0.000062
Streptococcus_peroris	Streptococcus	0.000	2.104	1.46E-02	0.000061	0.000012	0.000110
Alloprevotella_rava	Alloprevotella	0.000	0.913	2.04E-02	0.000111	0.000017	0.000204
Eggerthia_catenaformis	Eggerthia	0.000	1.868	1.45E-02	0.000134	0.000026	0.000241
Lachnoanaerobaculum_saburreum	Lachnoanaerobaculum	19.270	6.348	1.04E-02	0.000153	0.000036	0.000270
Treponema_medium	Treponema	0.000	2.151	1.49E-02	0.000166	0.000032	0.000300
Oribacterium_sinus	Oribacterium	0.000	5.806	4.61E-02	0.000185	0.000003	0.000366
Haemophilus_influenzae	Haemophilus	5.368	2.276	4.98E-02	0.000214	0.000000	0.000427
Rothia_dentocariosa	Rothia	8.255	7.945	4.11E-02	0.000236	0.000010	0.000462
Stomatobaculum_longum	Stomatobaculum	0.000	3.456	4.69E-02	0.000241	0.000003	0.000478
Lactobacillus_gasseri	Lactobacillus	0.000	8.476	2.30E-02	0.000241	0.000033	0.000448
Selenomonas_infelix	Selenomonas	0.000	0.630	3.95E-02	0.000258	0.000012	0.000503
Parascardovia_denticolens	Parascardovia	0.000	3.663	5.41E-03	0.000264	0.000078	0.000451
Granulicatella_adiacens	Granulicatella	0.000	7.345	2.81E-04	0.000368	0.000170	0.000567
Aeromonas_hydrophila	Aeromonas	35.269	3.351	1.14E-02	0.000398	0.000090	0.000707
Actinomyces_cardiffensis	Actinomyces	0.000	6.404	4.80E-03	0.000403	0.000123	0.000684
Campylobacter_ureolyticus	Campylobacter	48.621	16.185	2.18E-03	0.000426	0.000153	0.000698
Atopobium_rimae	Atopobium	19.792	9.165	7.26E-03	0.000473	0.000128	0.000819
Fretibacterium_fastidiosum	Fretibacterium	28.778	16.457	2.12E-04	0.000494	0.000233	0.000756
Slackia_exigua	Slackia	39.844	14.032	8.23E-03	0.000505	0.000130	0.000879
Treponema_maltophilum	Treponema	27.108	2.782	1.13E-02	0.000531	0.000120	0.000942
Aggregatibacter_segnis	Aggregatibacter	0.000	4.590	6.72E-05	0.000531	0.000270	0.000793
Dialister_micraerophilus	Dialister	60.311	12.450	3.46E-02	0.000545	0.000040	0.001050
Eubacterium_limosum	Eubacterium	53.987	13.826	5.26E-03	0.000630	0.000188	0.001073
Fusobacterium_periodonticum	Fusobacterium	28.179	5.797	5.68E-03	0.000638	0.000186	0.001090
Lactobacillus_salivarius	Lactobacillus	0.000	5.503	3.25E-02	0.000640	0.000053	0.001227
Selenomonas_sputigena	Selenomonas	28.421	6.764	3.04E-05	0.000660	0.000350	0.000970
Filifactor_alocis	Filifactor	42.615	13.783	6.90E-03	0.000669	0.000184	0.001154
Atopobium_parvulum	Atopobium	0.000	4.827	1.04E-06	0.000697	0.000417	0.000976
Prevotella_oris	Prevotella	0.000	2.958	7.73E-03	0.000803	0.000212	0.001393
Actinomyces_odontolyticus	Actinomyces	29.953	11.434	1.81E-02	0.000856	0.000146	0.001566
Streptococcus_pasteurianus	Streptococcus	0.000	10.036	5.61E-03	0.000874	0.000256	0.001493
Eikenella_corrodens	Eikenella	91.576	27.384	3.08E-02	0.000879	0.000082	0.001677
Enterococcus_faecalis	Enterococcus	33.522	13.255	3.89E-02	0.001067	0.000055	0.002080

species	genus	I2	qtest	pval	beta	beta_lb	beta_ub
Actinomyces_graevenitzii	Actinomyces	63.339	13.255	3.59E-03	0.001077	0.000352	0.001801
Gemella_haemolysans	Gemella	74.009	21.303	2.28E-02	0.001095	0.000152	0.002038
Peptoniphilus_harei	Peptoniphilus	65.465	11.640	1.39E-02	0.001184	0.000241	0.002128
Streptococcus_vestibularis	Streptococcus	0.322	5.017	5.65E-03	0.001372	0.000400	0.002343
Prevotella_nigrescens	Prevotella	0.000	8.520	1.93E-03	0.001388	0.000511	0.002265
Butyricimonas_synergistica	Butyricimonas	0.000	5.664	2.61E-02	0.001454	0.000173	0.002735
Catenibacterium_mitsuokai	Catenibacterium	0.969	3.589	4.62E-02	0.001480	0.000025	0.002934
Anaerococcus_vaginalis	Anaerococcus	69.369	29.173	4.21E-03	0.001570	0.000495	0.002645
Holdemania_filiformis	Holdemania	24.118	11.547	2.89E-02	0.001836	0.000189	0.003483
Prevotella_sp_oral_taxon_473	Alloprevotella	47.754	7.202	2.96E-02	0.002253	0.000224	0.004283
Anaerotruncus_colihominis	Anaerotruncus	67.105	21.698	2.53E-02	0.002410	0.000298	0.004522
Bilophila_wadsworthia	Bilophila	6.370	9.243	5.27E-03	0.002603	0.000774	0.004431
Bacteroides_nordii	Bacteroides	61.632	17.143	3.41E-03	0.002839	0.000939	0.004739
Porphyromonas_uenonis	Porphyromonas	98.190	35.660	1.99E-02	0.003165	0.000501	0.005828
Alloprevotella_tannerae	Alloprevotella	43.206	7.225	4.32E-03	0.003196	0.001001	0.005391
Peptostreptococcus_anaerobius	Peptostreptococcus	75.588	25.968	5.20E-04	0.003260	0.001419	0.005101
Porphyromonas_endodontalis	Porphyromonas	72.135	20.603	1.68E-02	0.003554	0.000641	0.006467
Coprococcus_catus	Coprococcus	27.146	6.513	2.47E-03	0.003785	0.001334	0.006236
Methanobrevibacter_smithii	Methanobrevibacter	0.000	12.925	4.71E-04	0.004105	0.001804	0.006407
Dorea_formicigenerans	Dorea	36.052	14.604	4.75E-02	0.004429	0.000048	0.008809
Alistipes_indistinctus	Alistipes	22.320	10.210	1.60E-03	0.004590	0.001739	0.007440
Parabacteroides_goldsteinii	Parabacteroides	59.316	20.728	2.94E-02	0.004712	0.000473	0.008950
Bacteroides_salyersiae	Bacteroides	24.239	9.644	1.50E-02	0.006506	0.001261	0.011752
Solobacterium_moorei	Solobacterium	68.179	25.835	2.50E-07	0.006529	0.004048	0.009010
Alistipes_shahii	Alistipes	68.615	24.114	4.18E-02	0.007011	0.000260	0.013761
Gemella_morbillorum	Gemella	79.702	44.631	2.97E-07	0.007261	0.004484	0.010037
Porphyromonas_asaccharolytica	Porphyromonas	96.677	40.404	2.13E-03	0.007546	0.002731	0.012362
Fusobacterium_nucleatum	Fusobacterium	82.361	46.141	2.54E-06	0.007587	0.004427	0.010748
Peptostreptococcus_stomatis	Peptostreptococcus	86.675	70.105	1.48E-05	0.008670	0.004748	0.012593
Alistipes_finegoldii	Alistipes	0.000	5.414	7.55E-05	0.009170	0.004630	0.013711
Parvimonas_micra	Parvimonas	76.353	21.811	2.05E-08	0.011228	0.007303	0.015152
Prevotella_intermedia	Prevotella	86.242	33.445	1.55E-03	0.011511	0.004381	0.018640
Flavonifractor_plautii	Flavonifractor	77.441	40.476	1.19E-02	0.011694	0.002582	0.020807
Ruminococcus_gnavus	Blautia	28.288	12.179	6.00E-03	0.013199	0.003784	0.022614
Bacteroides_caccae	Bacteroides	0.000	8.050	1.34E-04	0.014944	0.007274	0.022615
Bacteroides_thetaiotaomicron	Bacteroides	60.622	22.475	1.71E-02	0.015021	0.002671	0.027372
Odoribacter_splanchnicus	Odoribacter	84.271	38.607	7.33E-03	0.016471	0.004431	0.028512
Bacteroides_fragilis	Bacteroides	33.875	12.508	1.85E-03	0.020250	0.007499	0.033001
Akkermansia_muciniphila	Akkermansia	60.195	22.943	3.65E-03	0.024990	0.008141	0.041840
Escherichia_coli	Escherichia	73.109	26.213	1.72E-02	0.030256	0.005357	0.055156

Reference

1. Zeller G, Tap J, Voigt AY, et al. Potential of fecal microbiota for early-stage detection of colorectal cancer. Mol Sys Biol. 2014;10(11):766.
2. Feng Q, Liang S, Jia H, et al. Gut microbiome development along the colorectal adenoma-carcinoma sequence. Nat Commun. 2015;6:6528.
3. Yu J, Feng Q, Wong SH, et al. Metagenomic analysis of faecal microbiome as a tool towards targeted non-invasive biomarkers for colorectal cancer. Gut 2017;66(1):70-78.
4. Vogtmann E, Hua X, Zeller G, et al. Colorectal Cancer and the Human Gut Microbiome: Reproducibility with Whole-Genome Shotgun Sequencing. PLoS One 2016;11(5):e0155362.
5. Thomas AM, Manghi P, Asnicar F, et al. Metagenomic analysis of colorectal cancer datasets identifies cross-cohort microbial diagnostic signatures and a link with choline degradation. Nat Med. 2019; 25(4):667-678.
6. Wirbel J, Pyl PT, Kartal E, et al. Meta-analysis of fecal metagenomes reveals global microbial signatures that are specific for colorectal cancer. Nat Med. 2019;25(4):679-689.
7. Gupta A, et al. 2019 at https://www.ncbi.nlm.nih.gov/bioproject/PRJNA397112 (Indian Institute of Science Education and Research, Bhopal).
8. Yachida S, Mizutani S, Shiroma H, et al. Metagenomic and metabolomic analyses reveal distinct stage-specific phenotypes of the gut microbiota in colorectal cancer. Nat Med. 2019;25(6):968-976
